# Supplementary material for: Intestinal Fork Head Regulates Nutrient Absorption and Promotes Longevity
Source: Cell Rep. 2017 Oct 17;21(3):641–53. doi: 10.1016/j.celrep.2017.09.042 (PMC5656751; doi:10.1016/j.celrep.2017.09.042)
Supplement: Document S1. Supplemental Experimental Procedures, Figures S1–S7, and Tables S1–S8 [file mmc1.pdf]

Cell Reports, Volume *21*

Supplemental Information

Intestinal Fork Head Regulates Nutrient Absorption and Promotes Longevity

Ekin Bolukbasi, Mobina Khericha, Jennifer C. Regan, Dobril K. Ivanov, Jennifer Adcott, Miranda C. Dyson, Tobias Nespital, Janet M. Thornton, Nazif Alic, and Linda Partridge

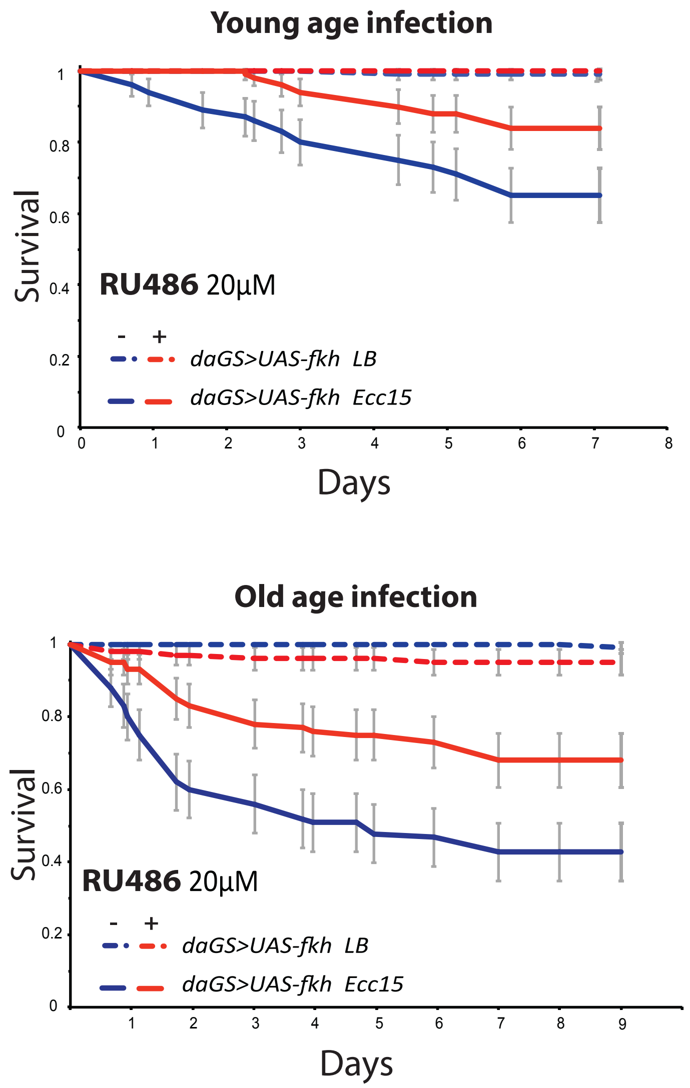

Figure S1: Over-expression of *fkh* improves immune response to *Ecc15* . Related to Figure 1. Survival curves of flies ubiquitously overexpressing *UAS-fkh* transgene under the control of *daGS* driver following systemic *Ecc15* infection or sham infection with LB only at 1 week of age (upper panel) or 7 weeks of age (lower panel). No significant difference in survival was observed at 20 µM RU486 in Sham infected flies at young (p= 0.317) and old age (p=0.093). Significant increase in survival following *Ecc15* infection was observed at 20 µM RU486 at young (p= 0.00014) and old age (p=0.0001).

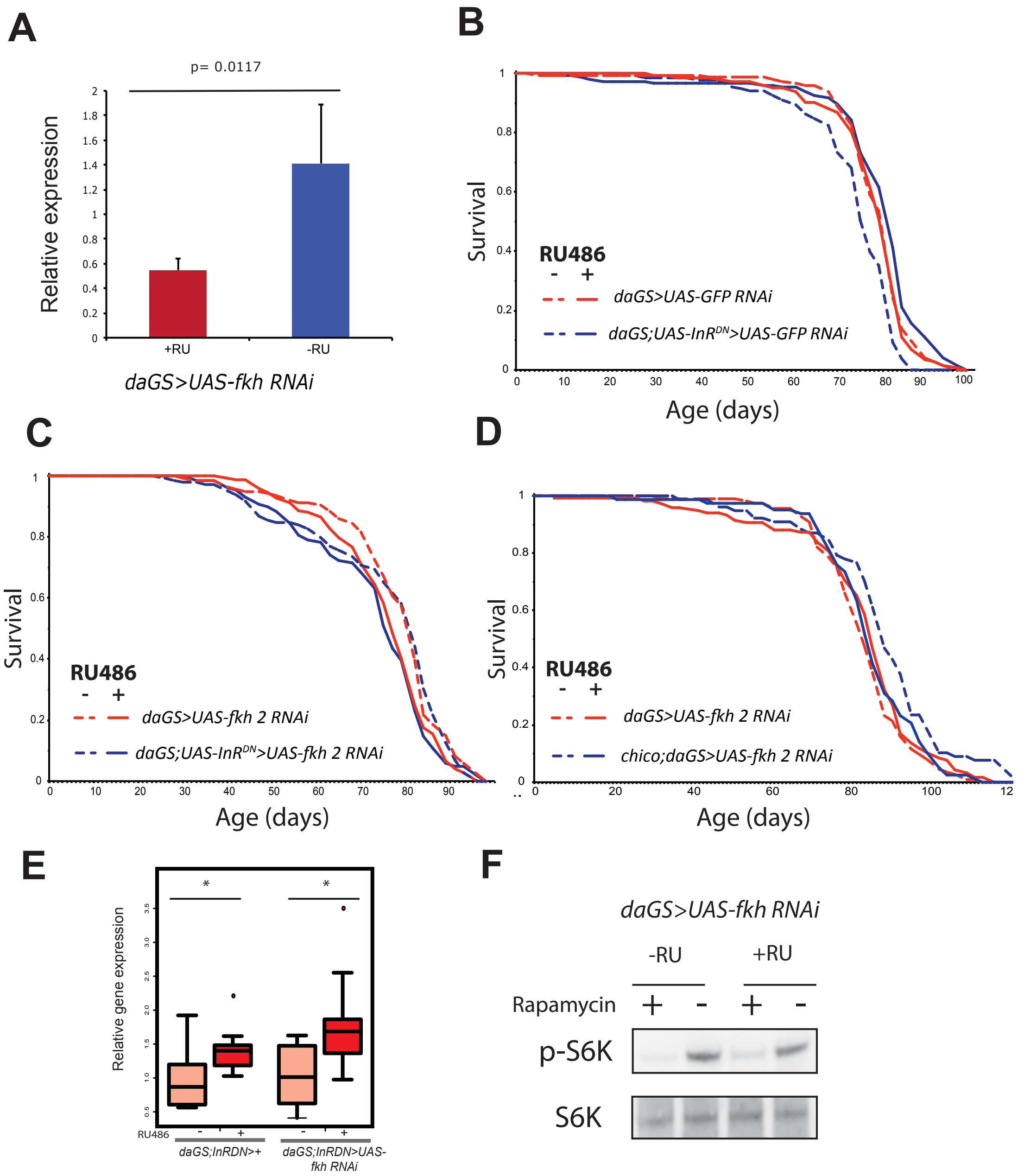

Figure S2: Over-expression of *fkh RNAi 2* transgene abolishes lifespan extension observed upon reduced IIS activity and IIS and rapamycin still exert downstream biochemical effects upon *fkh* RNAi. Related to Figure 3. (A) *fkh* mRNA was quantified relative to *actin5C* by qPCR in whole flies of genotype *daGS>UAS-fkh RNAi* induced or not with 200 µM RU486. Induction of *fkh* RNAi resulted in a significant decrease in *fkh* expression levels (Student's t-test, p< 0.05, n=5, error bars represent SD). (B) *daGS;UAS-InR*^{DN}*>UAS-GFP RNAi* females show increased lifespan in the presence of RU486 (p= 7.9×10^{-11}). Survival of *daGS >UAS-GFP RNAi* females was not different between –RU486 and +RU468 conditions (p= 0.78).). See also Table S6A (C) *daGS>UAS-fkh RNAi 2* and *daGS;UAS-InR*^{DN}*>UAS-fkh RNAi 2* flies showed significantly decreased lifespan in the presence of RU486 (p= 0.0075 and p= 0.0019 respectively). Reduction of IIS via simultaneous over-expression of *UAS-InR*^{DN} and *fkh RNAi 2* did not result in lifespan extension since no significant difference in survival was detected between *daGS>UAS-fkh RNAi 2* +RU486 and *daGS;UAS-InR*^{DN}*>UAS-fkh RNAi 2* +RU486 flies (p= 0.62).). See also Table S6B. (D) *chico* mutation extended lifespan in *daGS>UAS-fkh RNAi 2* flies in the absence of RU486 (p= 0.00031), but not in its presence (p=0.802). See also Table S6C. (E) *4EBP* mRNA was quantified relative to *tubulin* by qPCR in whole flies induced or not with 200 µM RU486. Reduction of IIS resulted in a significant increase in *4EBP* levels in both wild type and fkh RNAi background (Student's t-test, p< 0.05, n=9-10). (F) Immunoblotting of whole fly extracts using phospho-specific and total dS6K antibodies reveals decreased pS6K levels upon rapamycin treatment in flies induced or not with 200 µM RU486

A

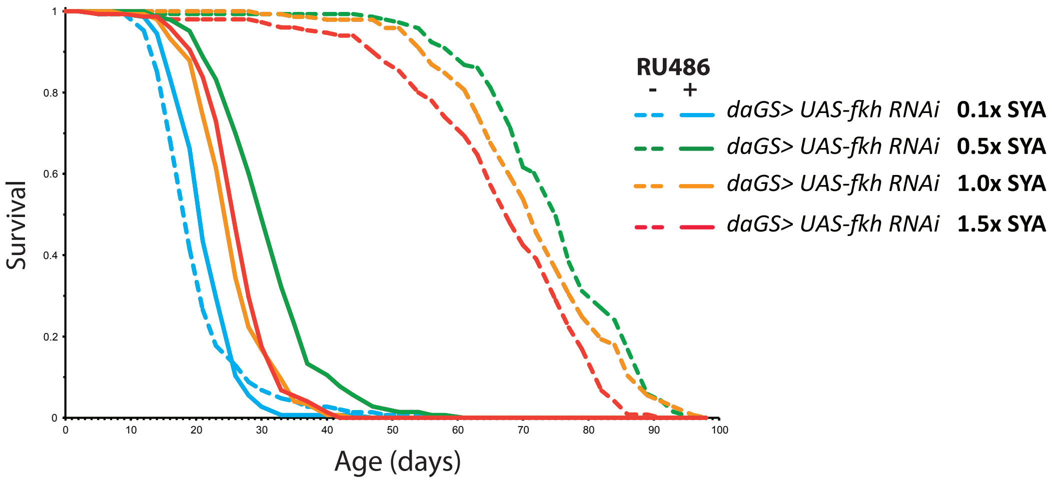

B

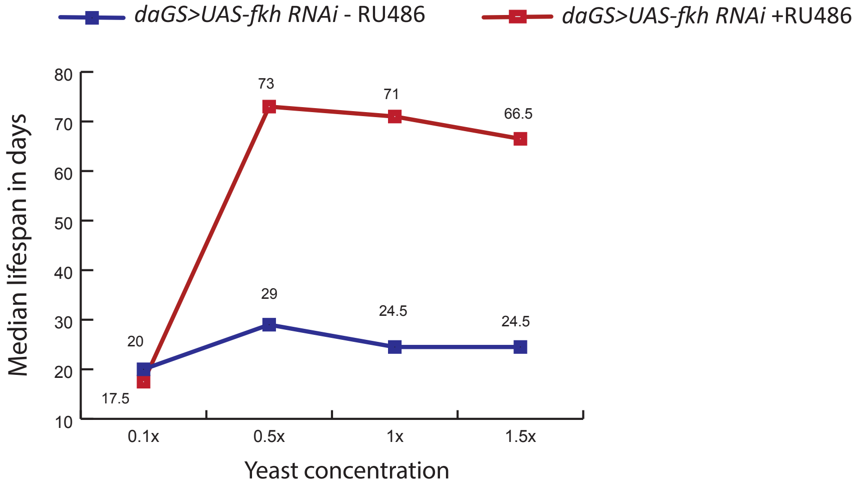

Figure S3: FKH does not mediate the response of lifespan to DR. Related to Figure 3. (A) Survival curves and (B) median lifespan values of *daGS> UAS-fkh RNAi* female flies in the presence or absence of RU486 and across different yeast concentrations (0.1x, 0.5x, 1.0x, and 1.5x yeast) on SYA food. Log-rank test revealed a significant difference in survival of *daGS>UAS-fkh RNAi* +RU486 flies at 0.5x yeast SYA food compared to *daGS>UAS-fkh RNAi* +RU486 flies at 1.0x and 1.5x yeast SYA food concentration ($p= 1.36 \times 10^{-10}$ and $p= 1.58 \times 10^{-9}$, respectively). For – RU486 conditions, at 0.1xyeast n= 147 deaths /9 censors , at 0.5xyeast n= 141 deaths /10 censors, at 1.0xyeast n= 145 deaths /3 censors, at 1.5xyeast n= 148 deaths /7 censors.). For +RU486 conditions, at 0.1xyeast n= 145 deaths /4 censors , at 0.5xyeast n= 143 deaths /8 censors, at 1.0xyeast n= 148 deaths /5 censors, at 1.5xyeast n= 148 deaths /4 censors.

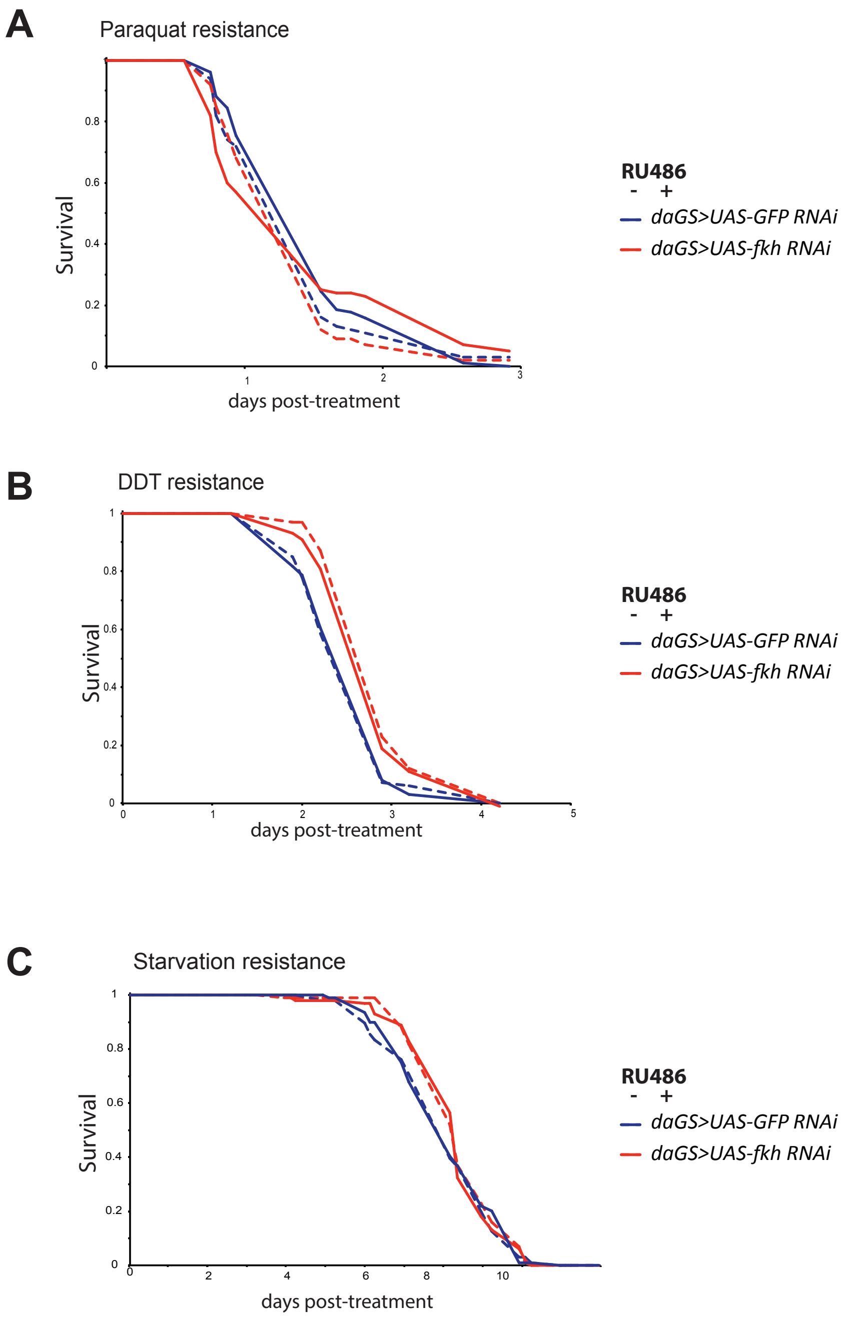

Figure S4: Over-expression of *GFP RNAi* or *fkh RNAi* alone does not affect stress resistance. Related to Figure 4. (A-C) Survival curves in response to paraquat treatment, DDT treatment and amino acid starvation of female flies induced to ubiquitously express either *UAS-GFP-RNAi* or *UAS-fkh-RNAi* transgenes (*daGS >UAS-GFP RNAi* or *daGS >UAS-fkh RNAi)* by using RU486 compared with uninduced controls. No significant difference in lifespan was observed between + and - RU486 conditions for *daGS >GFP RNAi* and for *daGS >fkh RNAi* upon (A) paraquat treatment (p= 0.072 and p=0.73 respectively), (B) DDT feeding (p= 0.93 and p=0.44 respectively) and (C) amino acid starvation (p= 0.78 and p=0.48 respectively). See also Table S7.

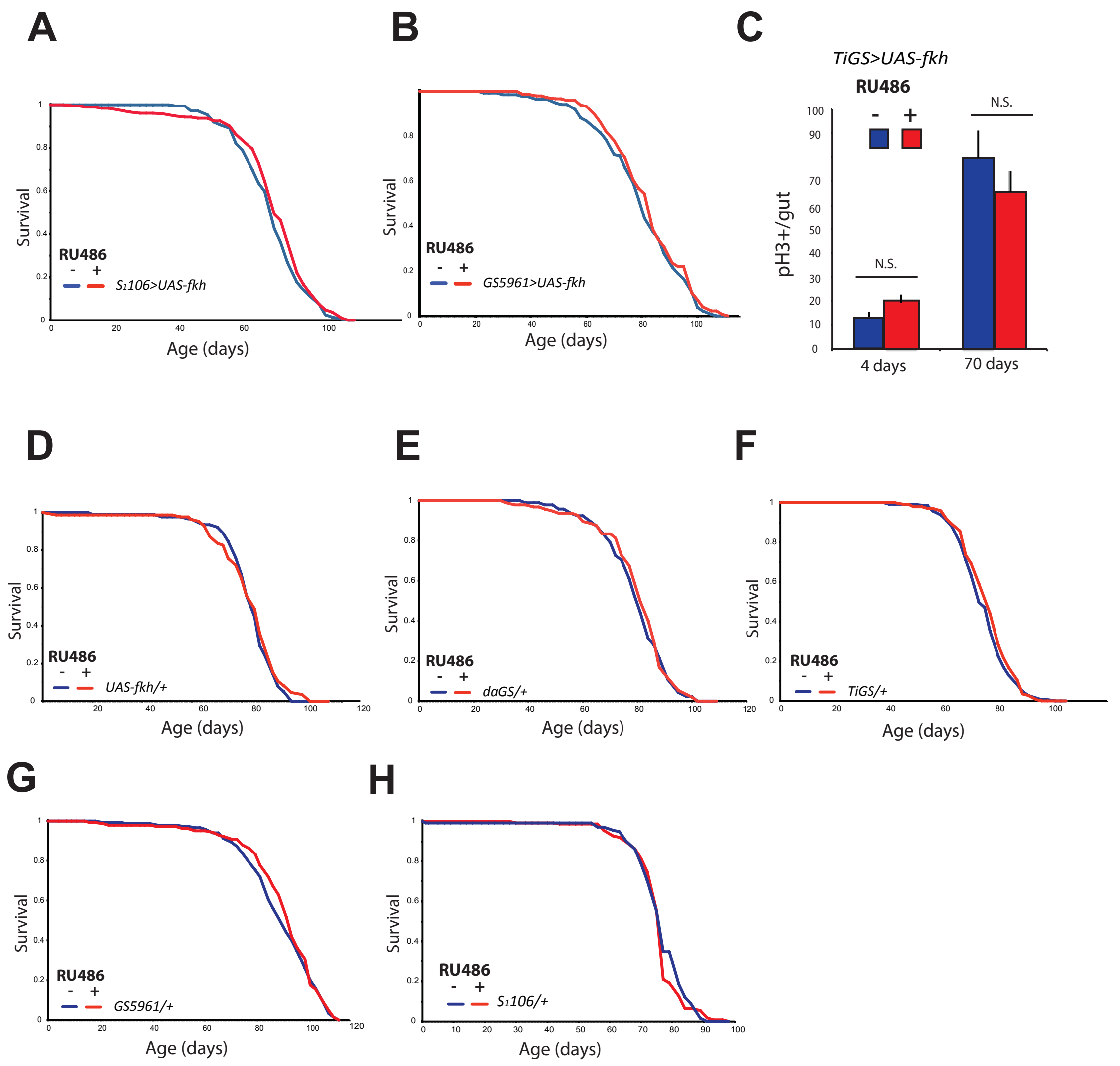

Figure S5: Overexpression of FKH in the progenitor cells does not affect lifespan and intestinal FKH up-regulation does not affect ISC proliferation. Related to Figure 5. (A) Adult abdominal fat body and gut specific over-expression of *fkh* transgene under the control of $S_1$106 driver did not affect lifespan (p= 0.093). (B) ISC and EB specific over-expression of the *fkh* transgene under the control of *GS5961* driver did not affect lifespan (p= 0.21). (C) Quantification of pH3+ cells per gut at 4 and 70 days of age in flies of genotype *TiGS>UAS-fkh* showed no difference in mitoses between – and + RU486 conditions in young and old flies (Student's t-test, p= 0.42 and p=0.51, n= 19 to 22 guts per condition, error bars represent SEM). See Also Table S8 A-B. (D) Presence of RU486 in the food did not affect survival of flies with *UAS-fkh* transgene alone (p= 0.48), (E) *daGS* driver alone (p=0.67), (F) TiGS driver alone (p=0.294), (G) *GS5961* driver alone (p= 0.38) and (H) *$S1_{106}$* driver alone (p=0.42). See also Table S8 C-G

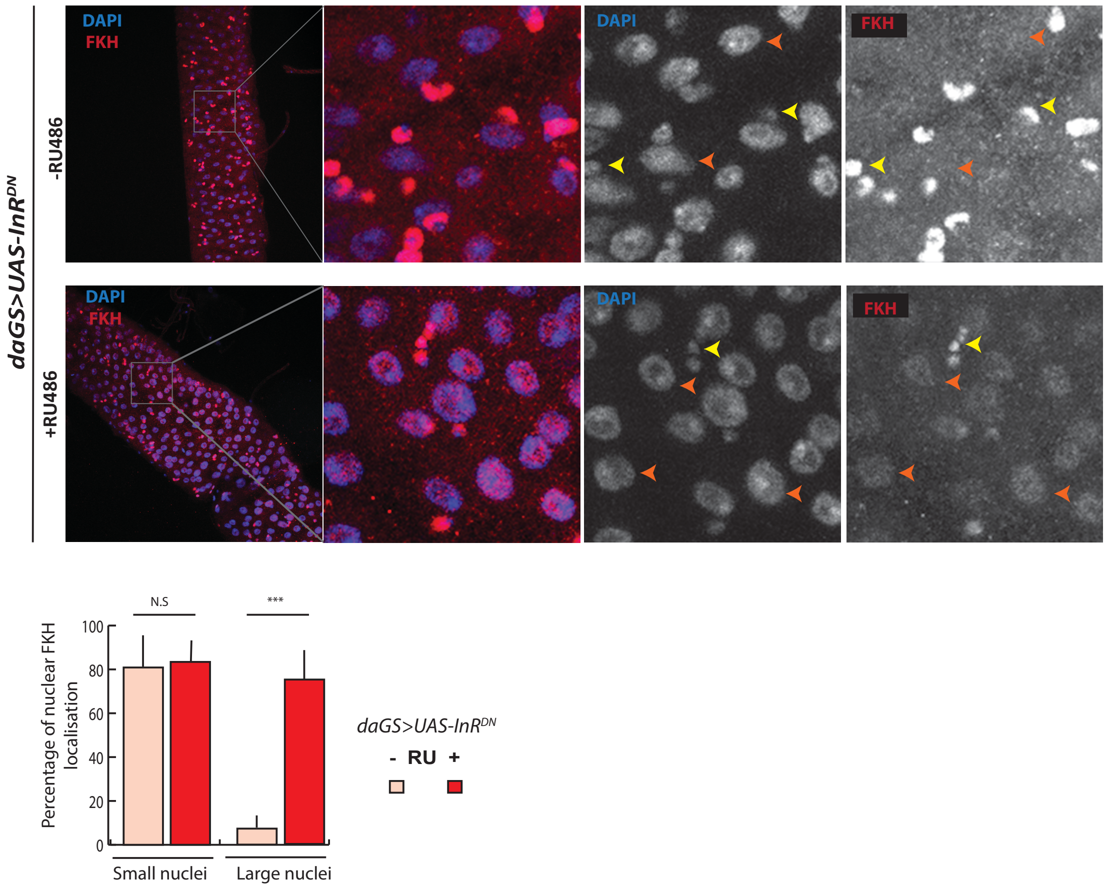

Figure S6: Reduced IIS leads nuclear localization of FKH in enterocytes. Related to Figure 6. *Drosophila* adult guts were dissected from flies of genotype *daGS>UAS-InR^{DN}* induced or not with 200 µM RU486. Endogenous FKH (red) and DAPI-stained nuclei (blue) were visualized by immunofluorescence in the mid-gut. Nuclear FKH localisation was quantified in two cell subtypes characterized by small and larger nuclei. Small nuclei and large nuclei are indicated by yellow and orange arrowheads respectively. Percentage of nuclear FKH staining was dramatically increased specifically in cells with large nuclei in the +RU486 condition (Student's t-test, $p< 0.0001$,n=5) whereas it remained unchanged in cells with smaller nuclei (Student's t-test, p=0.78, n=5, error bars represent SD).

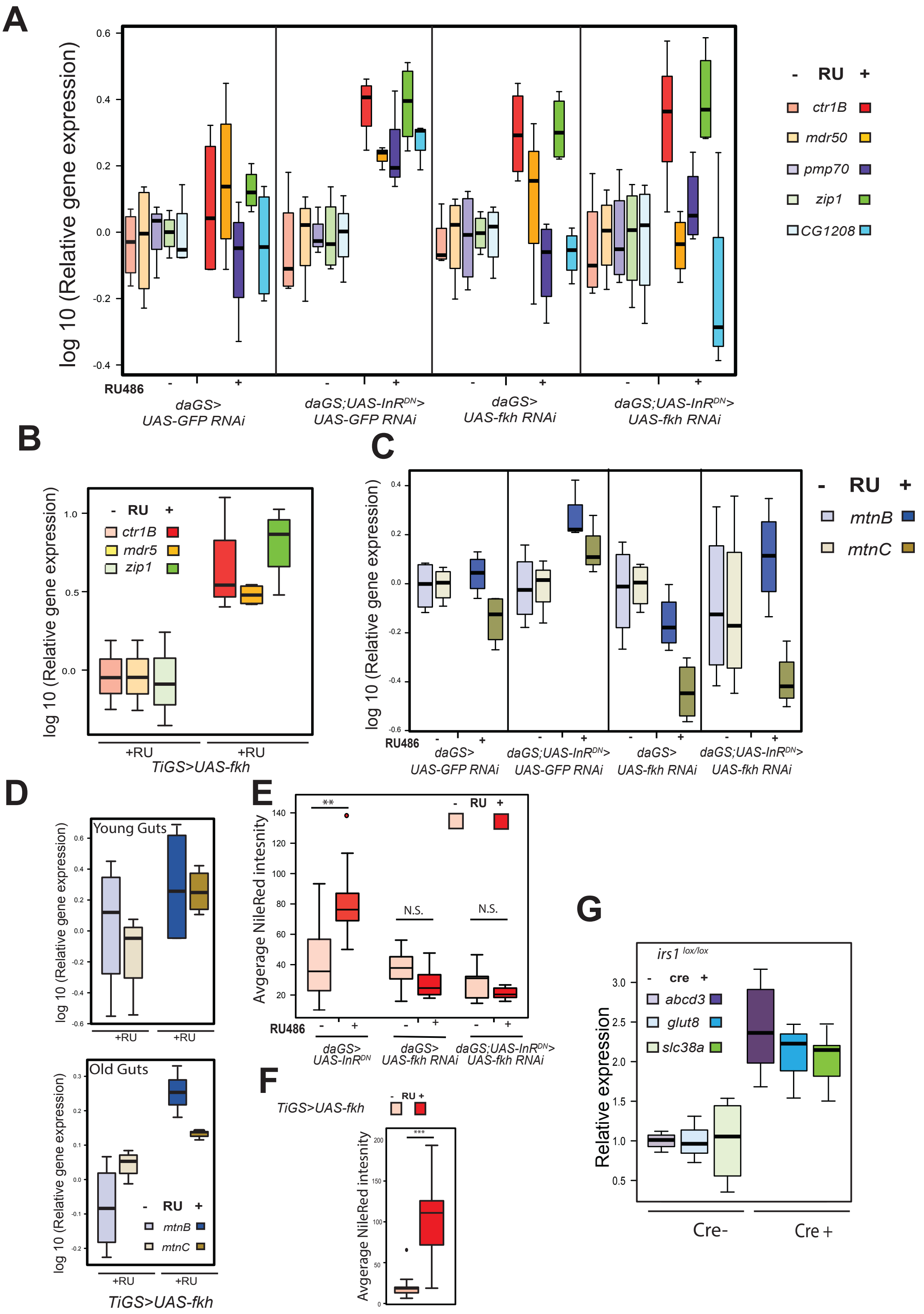

Figure S7: Expression of nutrient transporters and nutrient uptake is increased in the gut upon reduced IIS and FKH overexpression. Related to Figures 6 and 7. (A) *ctr1B*, *mdr50*, *zip1*, *pmp70* and *CG1208 mRNAs* were quantified relative to *actin5C* by qPCR in guts of *daGS >UAS-GFP RNAi, daGS;UAS-InR^{DN}>UAS-GFP RNAi, daGS >UAS-fkh RNAi and daGS;UAS-InR^{DN}>UAS-fkh RNAi* flies induced or not with RU486. Boxplots show log- 10 derived relative expression with – RU486 values set to zero. Data were analyzed with a linear model and the effects of the interaction between RU486 and genotype was significant (p=0.0004, n=3-4), whereas 3-way interaction between RU486, genotype and different gene categories was not significant (p= 0.2923). *daGS;UAS-InRDN>UAS-GFP RNAi* +RU condition showed significantly increased expression levels and was different form all other

conditions (t-test, p<0.05, n=3-4). (B) *ctr1B, mdr50 and zip1* mRNA levels quantified relative to *actin5C* by qRT-PCR in adult guts isolated from flies of genotype *TiGS>UAS-fkh* induced or not with 200 µM RU486. Boxplots show log-10 derived relative expression with - RU486 values set to zero. Induction of *fkh* resulted in a significant increase in *ctr1B, mdr50* and *zip1* expression levels (Student's t-test, p= 0.02, p= 0.006 and p= 0.007 respectively, n=4). (C) *mtnB* and *mtn*C mRNAs were quantified relative to *actin5C* by qPCR in guts of *daGS >UAS-GFP RNAi, daGS;UAS-InR^{DN}>UAS-GFP RNAi, daGS >UAS-fkh RNAi and daGS;UAS-InR^{DN}>UAS-fkh RNAi* flies induced or not with 200 µM RU486. Boxplots show log- 10 derived relative expression with – RU486 values set to zero. Data were analyzed with a linear model. The effect of interaction between RU and genotype was significant (p=0.0015, n=3-4) and the 3-way interaction between RU, genotype and different gene categories was not significant (p=0.5065, n=3-4). *daGS;UAS-InRDN>UAS-GFP RNAi* +RU condition was different from all others (t-test, p<0.05, n=3-4). (D) *mtnB* and *mtnC* mRNA levels quantified relative to *actin5C* by qPCR in young and old adult guts isolated from flies of genotype *TiGS>UAS-fkh* induced or not with 200 µM RU486. Boxplots show log-10 derived relative expression. Induction of *fkh* resulted in a significant increase in *mtnC* expression levels in young and old guts (t-test, p= 0.02 and p=0.005 respectively, n=4) and in a significant increase in *mtnB* expression levels in old guts (t-test, p= 0.01, n=4). (E) Quantification of the NileRed staining of the anterior region of mid-guts isolates from flies of genotypes *daGS>UAS-InR^{DN}*, *daGS;UAS-InR^{DN} > UAS-fkh RNAi* and *daGS > UAS-fkh RNAi* induced or not with 200 µM RU486. Boxplots show quantification of NileRed intensity in arbitrary units. NileRed staining is significantly increased in +RU486 condition of genotype *daGS>UAS-InR^{DN}* (t-test, p=0.0019, n=9-11). No significance was detected between + and – RU486 conditions of genotypes *daGS;UAS-InR^{DN} > UAS-fkh RNAi* and *daGS > UAS-fkh RNAi* (t-test, p=0.094 and p=0.059, respectively, n=9-11). (F) Quantification of the NileRed staining of the anterior region of mid-guts isolates from flies of genotype *TiGS>UAS-fkh* induced or not with 200 µM RU486. Boxplots show quantification of NileRed intensity in arbitrary units. NileRed staining is significantly increased in RU486 condition part of the mid-gut (t-test, p= 2.26 x 10^{-8}, n=15-19). (G) *Abcd3, Glut8 and Slc39a3* mRNA levels quantified relative to *actin* by qPCR in duodenum of 3 month old female mice of genotype *Irs1$^{lox/lox}$* (control) and *VillCre::Irs1$^{lox/lox}$* (*Irs1* knock-out). Boxplots show relative expression with control values set to 1. *Irs1* knock-out resulted in a significant increase in *Abcd3, Glut8* and *Slc39a* expression levels (Student's t-test, p= 0.006562, p=0.029 and p= 0.019 respectively, n=3-4).

Table S1- Ubiquitous FKH over expression extends lifespan. Related to Figure 1.

A. Survival data for da*GS>UAS-fkh* (related to Figure 1A)

Genotype/Treatment	Med Lifespan (days)	Max lifespan (days)	n Dead	n Censored	% Median Increase	% Maximum Increase	p-value (log rank) vs control
Repeat 1							
RU486-	79	95.5	93	5			
10 uM RU486	86	107	93	1	8.86	12.0418848	6.95E-06
50 uM RU486	84	102.5	98	2	6.33	7.32984293	0.017708512
100 uM RU486	81.5	101	92		3.16	5.7591623	0.181253206
200 uM RU486	52	93	105	1	-34.18	-2.617801	2.88501E-07

A. Survival data for da*GS>UAS-fkh* (related to Figure 1A)

Genotype/Treatment	Med Lifespan (days)	Max lifespan (days)	n Dead	n Censored	% Median Increase	% Maximum Increase	p-value (log rank) vs control
Repeat 1							
RU486-	78	98	90	4			
10 uM RU486	85	101	91	5	8.97	3.06122449	0.003
20 uM RU486	89.5	102	89	4	14.74359	4.08163265	6.3303E-06

Table S2- FKH is required for reduced IIS and rapamycin induced longevity. Related to Figure 3.

A. Survival data for da*GS;UAS-InRDN>GFP RNAi* +/-RU486 (related to Figure 3A)

Genotype/Treatment	Med Lifespan (days)	Max Lifespan (days)	n Dead	n Censored	% Median change (vs -RU)	% Maximum change (vs -RU)	p-value (log rank) vs control
Repeat 1							
daGS>GFP RNAiRU486-	62	78	150	0			
daGS>GFP RNAiRU486+	66.5	73.5	146	4			0.50
daGS;InRDN>GFP RNAiRU486-	64	78	140	10			
daGS;InRDN>GFP RNAiRU486+	73.5	80.5	146	4	14.84	3.205128205	4.45E-10

Genotype/Treatment	Med Lifespan (days)	Max Lifespan (days)	n Dead	n Censored	% Median change (vs -RU)	% Maximum change (vs -RU)	p-value (log rank) vs control
Repeat 2							
daGS>GFP RNAiRU486-	74.0	85.5	141	11			
daGS>GFP RNAiRU486+	74.0	85.5	148	12			0.918433719
daGS;InRDN>GFP RNAiRU486-	71.5	83.0	140	5			
daGS;InRDN>GFP RNAiRU486+	78.5	92.5	147	12	9.79	11.45	3.62E-09

B. Survival data for da*GS;UAS-InRDN>fkh RNAi* +/-RU486 (related to Figure 3B)

Genotype/Treatment	Med Lifespan (days)	Max Lifespan (days)	n Dead	n Censored	% Median change (vs -RU)	% Maximum change (vs -RU)	p-value (log rank)
Repeat 1							
daGS>fkh RNAiRU486-	76	90.5	130	5			
daGS>fkh RNAiRU486+	36	52.5	146	2	-52.63157895	-41.99	1.97E-54 (vs RU-)
daGS;InRDN>fkh RNAiRU486-	80.5	94.5	131	8			
daGS;InRDN>fkh RNAiRU486+	31.5	59.5	150	7	-60.87	-37.04	2.59E-63 (vs RU-)
					daGS>fkh RNAiRU486<br>daGS;InRDN>fkh RNAiRU486+	vs.	**0.08**

Genotype/Treatment	Med Lifespan (days)	Max Lifespan (days)	n Dead	n Censored	% Median change (vs -RU)	% Maximum change (vs -RU)	p-value (log rank)
Repeat 2							
daGS>fkh RNAiRU486-	76	92.5	154	4			
daGS>fkh RNAiRU486+	31.5	43.5	143	0	-58.55	-52.97	1.54 (vs RU-)
daGS;InRDN>fkh RNAiRU486-	76	85.5	136	9			
daGS;InRDN>fkh RNAiRU486+	32	50.5	154	8	-57.89	-40.94	3.07(vs RU-)
						daGS>fkh RNAiRU486+<br>daGS;InRDN>fkh RNAiRU486+	**0.11**

C.Survival data for daGS;chico> GFP RNAi(related to Figure 3C)

Genotype/Treatment	Med Lifespan (days)	Max Lifespan (days)	n Dead	n Censored	% Median change (vs wt)	% Maximum change (vs wt)	p-value (log rank)
Repeat 1							
daGS>GFP RNAiRU486-	83	97	142	1			
daGS>GFP RNAiRU486+	85	96	152	3			
chico;daGS>GFP RNAiRU486-	90	108.5	90	0	8.43	11.86	2.71E-06
chico;daGS>GFP RNAiRU486+	90	106	89	5	5.88	10.42	6.28E-07

D.Survival data for daGS;chico> UAS-fkh RNAi(related to Figure 3D)

Genotype/Treatment	Median Lifespan (days)	Maximum lifespan	n Dead	n Censored	% Median change (vs wt)	% Maximum change (vs wt)	p-value (log rank)
Repeat 1							
daGS>fkh RNAiRU486-	83	97	127	2			
daGS>fkh RNAiRU486+	37	64	113	1			
chico;daGS>fkh RNAiRU486-	90	113	115	2	8.43	16.49	3.89E-08
chico;daGS>fkh RNAiRU486+	38	66.5	122	2	2.70	3.91	0.61

Genotype/Treatment	Median Lifespan (days)	Maximum lifespan	n Dead	n Censored	% Median change (vs wt)	% Maximum change (vs wt)	p-value (log rank)
Repeat 2							
daGS>fkh RNAiRU486-	73	82.5	144	3			
daGS>fkh RNAiRU486+	29	49	142	1			
chico;daGS>fkh RNAiRU486-	75.5	89.5	142	3	3.42	8.48	2.06E-04
chico;daGS>fkh RNAiRU486+	26.5	47.5	150	0	-8.62	-3.06	0.15

E.Survival data for daGS> UAS-fkh RNAi +/- rapamycin (related to Figure 3E)

Genotype/Treatment	Median Lifespan (days)	Maximum lifespan	n Dead	n Censored	% Median change (vs -Rapa)	% Maximum change (vs -Rapa)	p-value (log rank)
Repeat 1							
daGS>fkh RNAiRU486-	69	73.5	147	5			
daGS>fkh RNAiRU486+	24.5	31.5	150	3			
daGS>fkh RNAiRU486- Rapa+	73.5	80.5	137	12	6.52	9.52	2.37E-08
daGS>fkh RNAiRU486+ Rapa+	24.5	31.5	146	4		0.00	0.82

Genotype/Treatment	Median Lifespan (days)	Maximum lifespan	n Dead	n Censored	% Median change (vs -Rapa)	% Maximum change (vs -Rapa)	p-value (log rank)
Repeat 2							
daGS>fkh RNAiRU486-	69.0	78.0	128	5			
daGS>fkh RNAiRU486+	27.0	45.5	114	10			
daGS>fkh RNAiRU486- Rapa+	73.5	89.0	106	8	6.52	14.10	3.05E-04
daGS>fkh RNAiRU486+ Rapa+	27.0	41.0	125	3		-9.89	0.34

Table S3- Details of CPH analysis. Related to Figures 3, 4AC and 5C.

A. Lifespan daGS;UAS-InRDN> +/- fkh RNAi(related to Figure 3AB)

Cox Proportional Hazard (CPH) analysis

Coefficient	estimate	exp(coeff)	SE (coeff)	z	p
RU486	0.07415	1.07697	0.12121	0.612	0.5407
InRDN	-0.32604	0.72178	0.12152	-2.683	0.0073 **
fkh RNAi	-1.35544	0.25783	0.12946	-10.47	< 2e-16 ***
RU486:InRDN	-0.67008	0.51167	0.17101	-3.918	8.91e-05 ***
RU486:fkh RNAi	3.31408	27.49708	0.18504	17.91	< 2e-16 ***
InRDN:fkh RNAi	0.12494	1.13308	0.17424	0.717	0.4733
RU486:InRDN vs fkh RNAi	0.48914	1.63091	0.24198	2.021	0.0432 *

B. Lifespan daGS;chico> +/- fkh RNAi(related to Figure 3CD)

Cox Proportional Hazard (CPH) analysis

Coefficient	estimate	exp(coeff)	SE (coeff)	z	p
RU486	-0.05856	0.94312	0.1355	-0.432	0.66563
chico	-0.66237	0.51563	0.13714	-4.83	1.37e-06 ***
fkh RNAi	-0.15165	-0.15165	0.12239	-1.239	0.21533
RU486 : chico	0.08379	1.0874	0.19071	0.439	0.66039
RU486 : fkh RNAi	3.4651	31.97972	0.21699	15.969	< 2e-16 ***
chico : fkhRNAi	-0.18896	0.82782	0.18949	-0.997	0.31868
RU486:chico vs fkh RNAi	0.76163	2.14177	0.26825	2.839	0.00452 **

C. Lifespan daGS> fkh RNAi +/- rapamycin (related to Figure 3E)

Cox Proportional Hazard (CPH) analysis

Coefficient	estimate	exp(coeff)	SE (coeff)	z	p
RU486	4.1513	63.5159	0.2791	14.876	< 2e-16 ***
Rapamycin	-0.6045	0.5464	0.1225	-4.936	7.99e-07 ***
RU486 : Rapamycin	0.5865	1.7976	0.1698	3.454	0.000552 ***

D. Paraquat resistance daGS;UAS-InRNDN> +/- fkh RNAi(related to Figure 4A)

Cox Proportional Hazard (CPH) analysis

Coefficient	estimate	exp(coeff)	SE (coeff)	z	p
RU486	-0.4351	0.6472	0.1486	-2.928	0.00342 **
fkh RNAi	-0.2492	0.7794	0.1503	6.996	0.07896
RU486: fkhRNAi	-0.1071	0.8985	0.2052	-0.522	0.60192

E. Starvation resistance daGS;UAS-InRNDN> +/- fkh RNAi(related to Figure 4C)

Cox Proportional Hazard (CPH) analysis

Coefficient	estimate	exp(coeff)	SE (coeff)	z	p
RU486	-0.59455	0.55181	0.14653	-4.057	4.96e-05 ***
fkh RNAi	0.07632	1.07931	0.13956	0.547	0.584
RU486: fkhRNAi	0.83095	2.29551	0.20347	4.084	4.43e-05 ***

F. Lifespan TiGS> fkh RNAi +/- rapamycin (related to Figure 5C)

Cox Proportional Hazard (CPH) analysis

Coefficient	estimate	exp(coeff)	SE (coeff)	z	p
RU486	0.02691	1.02728	0.02054	1.31	0.19
Rapamycin	-0.24298	0.78429	0.01997	-12.167	< 2e-16 ***
RU486 : Rapamycin	0.22289	1.24968	0.02861	7.792	6.66e-15 ***

Table S4- FKH is required for IIS-induced starvation resistance. Related to Figure 4A-C.

A. Paraquat resistance data for da*GS;UAS-InRDN>GFP RNAi or FKH RNAi* +/-RU486 (related to Figure 4A)

Genotype/Treatment	Med Survival (days)	Max Survival (days)	n Dead	p-value (log rank) vs -RU
Repeat 1				
daGS;InRDN>GFP RNAiRU486-	1.24	2.23	99	
daGS;InRDN>GFP RNAiRU486+	1.24	-	85	5.89E-04
daGS;InRDN>fkh RNAiRU486-	1.24	2.23	103	
daGS;InRDN>fkh RNAiRU486+	2.23	2.23	98	1.47E-07

Genotype/Treatment	Med Survival (days)	Max Survival (days)	n Dead	p-value (log rank) vs -RU
Repeat 2				
daGS;InRDN>GFP RNAiRU486-	0.60	2.13	94	
daGS;InRDN>GFP RNAiRU486+	1.15	7.92	100	4.43244E-05
daGS;InRDN>fkh RNAiRU486-	0.60	1.56	98	
daGS;InRDN>fkh RNAiRU486+	1.04	3.56	97	4.98E-10

B. DDT resistance data for da*GS;UAS-InRDN>GFP RNAi or FKH RNAi* +/-RU486 (related to Figure 4B)

Genotype/Treatment	Med Survival (days)	Max Survival (days)	n Dead	p-value (log rank) vs -RU
Repeat 1				
daGS;InRDN>GFP RNAiRU486-	2.6	3.7	90	
daGS;InRDN>GFP RNAiRU486+	3.7	-	88	1.34E-03
daGS;InRDN>fkh RNAiRU486-	2.6	3.7	96	
daGS;InRDN>fkh RNAiRU486+	2.6	3.7	92	2.80E-04

Genotype/Treatment	Med Survival (days)	Max Survival	n Dead	p-value (log rank) vs -RU
Repeat 2				
daGS;InRDN>GFP RNAiRU486-	3.61	4.65	91	
daGS;InRDN>GFP RNAiRU486+	4.13	6.61	92	3.34E-08
daGS;InRDN>fkh RNAiRU486-	3.19	4.65	100	
daGS;InRDN>fkh RNAiRU486+	4.13	-	104	2.32E-12

C.Starvation resistance data for daGS;UAS-InRDN>GFP RNAi or FKH RNAi +/-RU486 (related to Figure 4C)

Genotype/Treatment	Med Survival (days)	Max Survival (days)	n Dead	p-value (log rank) vs -RU
Repeat 1				
daGS;InRDN>GFP RNAiRU486-	7.64	8.61	102	
daGS;InRDN>GFP RNAiRU486+	8.61	9.51	95	3.67E-05
daGS;InRDN>fkh RNAiRU486-	7.64	8.61	105	
daGS;InRDN>fkh RNAiRU486+	7.64	8.61	101	0.085

Genotype/Treatment	Med Survival (days)	Max Survival (days)	n Dead	p-value (log rank) vs -RU
Repeat 2				
daGS;InRDN>GFP RNAiRU486-	6.99	8.63	90	
daGS;InRDN>GFP RNAiRU486+	7.60	9.54	88	5.58E-04
daGS;InRDN>fkh RNAiRU486-	6.15	7.60	96	
daGS;InRDN>fkh RNAiRU486+	6.21	6.55	92	0.70

Table S5- Intestinal overexpression of FKH extends lifespan. Related to Figures 5 and 7E.

A. Survival data for Ti*GS>UAS-fkh* (related to Figure 5A)

Genotype/Treatment	Med Lifespan (days)	Max Lifespan (days)	n Dead	n Censored	% Median change	% Maximum change	p-value (log rank) vs control
Repeat 1							
RU486-	66	78	219	7			
RU486+	69	82	212	5	4.5	5.13	0.001049071
Repeat 2							
RU486-	74	88	148	3			
RU486+	79	97	147	3	6.7	10.23	0.000153624
Repeat 3							
RU486-	103	114	119	1			
RU486+	108	124	119	2	4.8	8.77	4.16098E-05

B. Survival data for Ti*GS>fkh RNAi +/- rapa* (related to Figure 5C)

Genotype/Treatment	Med Lifespan (days)	Max Lifespan (days)	n Dead	n Censored	% Median change	% Maximum change	p-value (log rank) vs -rapa control
TiGS>fkh RNAiRU486-	80	94	146	1			
TiGS>fkh RNAiRU486+	80	94	146	2			
TiGS>fkh RNAiRU486- Rapa+	89	98	148		11.25	4.255319149	4.60E-10
TiGS>fkh RNAiRU486+ Rapa+	82	91.5	146	1			0.575678595

C. Survival data for Ti*GS>UAS-InRDN* (related to Figure 5D)

Genotype/Treatment	Med Lifespan (days)	Max Lifespan (days)	n Dead	n Censored	% Median change	% Maximum change	p-value (log rank) vs control
Repeat 1							
RU486-	85	104	147	3			0.014845219
RU486+	87.5	116.5	141	9	3	12	
Repeat 2							
RU486-	76	92	131	5			
RU486+	80.5	101.5	142	4	6	10.3	0.000289316

D. Survival data for Ti*GS;UAS-fkh RNAi> UAS-InRDN* (related to Figure 5E)

Genotype/Treatment	Med Lifespan (days)	Max Lifespan (days)	n Dead	n Censored	% Median change	% Maximum change	p-value (log rank) vs control
Repeat 1							
RU486-	83	94.5	126	15			
RU486+	73	87	136	4	-12	-7	8.97448E-07
Repeat 2							
RU486-	78	87.5	134	7			
RU486+	73.5	87.5	145	3	-5.7	0	0.000300082

E. Survival data for Ti*GS>UAS-fkh RNAi* (related to Figure 5E)

Genotype/Treatment	Med Lifespan (days)	Max Lifespan (days)	n Dead	n Censored	% Median change	% Maximum change	p-value (log rank) vs control
Repeat 1							
RU486-	87.5	105	144	1			
RU486+	83	106	141	1			0.08
Repeat 2							
RU486-	78	92	89	3			0.829119039
RU486+	76	94	79	10			

F. Survival data for *GS5966>UAS-fkh* (related to Figure 7E)

Genotype/Treatment	Med Lifespan (days)	Max Lifespan (days)	n Dead	n Censored	% Median change	% Maximum change	p-value (log rank) vs control
RU486-	71	87	139	12			
RU486+	82	92	139	8	15.4929577	5.747126437	2.03614E-07

Table S6- FKH is required for reduced IIS-induced longevity. Related to Figures S2B-D.

A. Survival data for da*GS;UAS-InRDN>GFP RNAi* +/-RU486 (related to Figure S2B)

Genotype/Treatment	Med Lifespan (days)	Max Lifespan (days)	n Dead	n Censored	% Median change (vs -RU)	% Maximum change (vs -RU)	p-value (log rank) vs control
daGS>GFP RNAiRU486-	90.5	87.5	144	4			
daGS>GFP RNAiRU486+	90.5	87.5	143	2	0.00	0.00	0.79
daGS;InRDN>GFP RNAiRU486-	76.0	80.5	131	2			
daGS;InRDN>GFP RNAiRU486+	80.5	92	146	4	5.92	14.29	7.91E-11

B. Survival data for da*GS;UAS-InRDN>fkh RNAi 2* +/-RU486 (related to Figure S2C)

Genotype/Treatment	Med Lifespan (days)	Max lifespan	n Dead	n Censored	% Median change (vs -RU)	% Maximum change (vs -RU)	p-value (log rank)
daGS>fkh RNAi2 RU486-	80.5	92	134	7			
daGS>fkh RNAi 2 RU486+	76	87.5	149	1	-5.590062112	-4.8913043	0.007503122
daGS;InRDN>fkh RNAi 2 RU486-	80.5	92	143	3			
daGS;InRDN>fkh RNAi 2 RU486+	76	90	123	7	-5.590062112	-2.173913	0.019521489
					daGS>fkh RNAiRU486 daGS;InRDN>fkh RNAiRU486+	vs.	**0.623024154**

D.Survival data for daGS;chico> UAS-fkh RNAi(related to Figure S2D)

Genotype/Treatment	Med Lifespan (days)	Max Lifespan (days)	n Dead	n Censored	% Median change (vs wt)	% Maximum change (vs wt)	p-value (log rank)
daGS>fkh RNAi2 RU486-	83	101.5	112				
daGS>fkh RNAi2 RU486+	85	106	118	1			
chico;daGS>fkh RNAi2 RU486-	87.5	120	77	3	5.42	18.23	3.15E-04
chico;daGS>fkh RNAi2 RU486+	85	103	78	3		-2.83	0.80

Table S7- GFP or FKH RNAi alone does not affect survival to stress. Related to Figure S4.

A. Paraquat resistance data for da*GS>GFP RNAi or FKH RNAi* +/-RU486 (related to Figure S4A)

Genotype/Treatment	Med Survival (days)	Max Survival (days)	n Dead	-value (log rank) vs -RU
daGS>GFP RNAiRU486-	1.24	2.23	97	
daGS>GFP RNAiRU486+	1.24	2.23	98	7.29E-02
daGS>fkh RNAiRU486-	1.24	2.23	102	
daGS>fkh RNAiRU486+	1.24	2.23	95	7.32E-01

B. DDT resistance data for da*GS>GFP RNAi or FKH RNAi* +/-RU486 (related to Figure S4B)

Genotype/Treatment	Med Survival (days)	Max Survival (days)	n Dead	-value (log rank) vs -RU
daGS>GFP RNAiRU486-	2.55	2.55	99	
daGS>GFP RNAiRU486+	2.55	2.55	100	9.30E-01
daGS>fkh RNAiRU486-	2.55	3.70	101	
daGS>fkh RNAiRU486+	2.55	3.70	99	4.41E-01

C. Starvation resistance data for da*GS>GFP RNAi or FKH RNAi* +/-RU486 (related to Figure S4C)

Genotype/Treatment	Med Survival (days)	Max Survival (days)	n Dead	-value (log rank) vs -RU
daGS>GFP RNAiRU486-	8.23	10.56	99	
daGS>GFP RNAiRU486+	7.60	9.54	94	9.47E-01
daGS>fkh RNAiRU486-	8.23	9.54	96	
daGS>fkh RNAiRU486+	7.60	8.63	94	1.23E-01

Table S8- Survival data Related to Figure S5

A. Survival data for S106>*UAS-fkh* (related to Figure S5A)

Genotype/Treatment	Med Lifespan (days)	Max lifespan (days)	n Dead	n Censored	% Median change	% Maximum change	p-value (log rank) vs control
RU486-	68.5	85	211	6			
RU486+	68.5	85	209	6			0.093464441

B. Survival data for GS5961>*UAS-fkh* (related to Figure S5B)

Genotype/Treatment	Median Lifespan (days)	Maximum lifespan (days)	n Dead	n Censored	% Median change	% Maximum change	p-value (log rank) vs control
RU486-	80	99	134	9			
RU486+	82.5	101	132	6			0.215031421

C. Survival data for UAS-fkh>*+* (related to Figure S5D)

Genotype/Treatment	Median Lifespan (days)	Maximum lifespan (days)	n Dead	n Censored	% Median change	% Maximum change	p-value (log rank) vs control
RU486-	78.5	90	85	7			
RU486+	78.5	92.5	86				0.484519478

D. Survival data for daGS>*+* (related to Figure S5E)

Genotype/Treatment	Median Lifespan (days)	Maximum lifespan (days)	n Dead	n Censored	% Median change	% Maximum change	p-value (log rank) vs control
RU486-	80	93	94	2			
RU486+	80	93	95	2			0.675801839

E. Survival data for TiGS>*+* (related to Figure S5F)

Genotype/Treatment	Median Lifespan (days)	Maximum lifespan (days)	n Dead	n Censored	% Median change	% Maximum change	p-value (log rank) vs control
RU486-	74.5	88	147	3			
RU486+	76	88	144	4			0.83062806

F. Survival data for GS5961>+ (related to Figure S5G)

Genotype/Treatment	Median Lifespan (days)	Maximum lifespan (days)	n Dead	n Censored	% Median change	% Maximum change	p-value (log rank) vs control
RU486-	89	106	150	1			
RU486+	92	106	143	2			0.38072779

G. Survival data for S106>+ (related to Figure S5H)

Genotype/Treatment	Median Lifespan (days)	Maximum lifespan (days)	n Dead	n Censored	% Median change	% Maximum change	p-value (log rank) vs control
RU486-	75	86	135	4			
RU486+	74	87.5	134	4			0.489215204

Supplemental Experimental Procedures

Lifespan Experiments

RU486 (Sigma) dissolved in ethanol was added to a final concentration of 200 µM, unless otherwise indicated. Rapamycin (LC Laboratories) was diluted in ethanol and added to SYA food at 100 µM concentration. Stocks were backcrossed for at least six generations into the wild-type outbred *wDahomey* population. For lifespans, flies were sorted into experimental vials at a density of 10 or 15 flies per vial. Flies were transferred to fresh vials three times a week, and deaths/censors were scored during the transfer.

Fly Stocks

The following stocks were used: *UAS-InR*^{DN} (*K1409A*) and *UAS-GFP-RNAi* stocks were obtained from the Bloomington Stock Centre. *UAS-fkh-RNAi* line was obtained from Vienna stock center (37062). *daGS* was kindly provided by Veronique Monnier (Tricoire et al., 2009). *TiGS* and $S_1$106 drivers were described in Poirier et al. 2008 (Poirier et al., 2008). The alternative *UAS-fkh-RNAi* (*fkh RNAi 2*) stock was kindly provided by Michael Junger (Bulow et al., 2010). d*foxo*$^{\Delta/\Delta}$ line was described in Slack et al., 2011 (Slack et al., 2011) . To create *UAS-FKH lines*, *Fork Head* genomic sequences encoding the full-length wild-type fkh protein sequence open reading frame were cloned into pENTR-D vector, confirmed by sequencing and transferred to the pUASg.attB (Bischof et al., 2007)P-element-based vector using standard Gateway cloning techniques (Invitrogen).

Paraquat, DDT and Fecundity Assays

Paraquat, DDT and fecundity assays were carried out as described by Slack et al., (Slack et al., 2011) with the following modification: the final concentration of DDT used in the xenobiotic assay was increased to 0.06% (w/v) DDT.

Dietary Restriction

The DR protocol was described in detail in Bass et al., (Bass et al., 2007)

S2 cell culture and transfections

S2 cells were routinely cultured in Schneider's insect medium (Sigma) supplemented with 10% FBS. S2 cell transfections were carried out by using Effectene transfection reagent (Qiagen) according to manufacturer's instructions. To express FKH-HA and dAKT-FLAG in S2 cells, full length wild type FKH and dAKT open reading frames were cloned into pAHW and pAFW *Drosophila* Gateway vectors (a gift from Martin Junger) respectively by using standard Gateway cloning techniques (Invitrogen).

Preparation of PhosTag SDS-PAGE Gels

24 hour following transfections, S2 cells were serum starved for 2 hours and stimulated with 1 µM human insulin (Sigma) for the indicated duration. Protein extracts were prepared by lysing the cells in RIPA buffer (NEB labs) containing PhoSTOP (Roche) and Complete Protease inhibitors (Roche). SDS-PAGE gels were prepared at 7.5% acrylamide concentration and contained 50 mM Phos-Tag^{TM} (Wako). Gels were run at 50 V until samples entered the top one third of the resolving gel at which point the voltage was increased to 140 V.

Immunoprecipitation

Prior to immunoprecipitations (IPs), S2 cells were stimulated with 1 µM human insulin (Sigma) for 15 minutes. S2 cells were washed in cold PBS and lysed in cold lysis buffer (50 mM HEPES-KOH pH 8, 100 mM KCL, 5 mM EDTA, 10% glycerol, 0.5% NP-40) containing PhoSTOP (Roche) and Complete

Protease Inhibitors (Roche). IPs were carried out by using either anti-HA coupled agarose beads (Sigma,) or anti-FLAG coupled agarose beads (Sigma, F2426) according to manufacturer's instructions.

Immunoblotting

Protein samples were resolved at 140 V by SDS-PAGE on precast 4–12% Bis-Tris polyacrylamide gels (Novex) and transferred onto nitrocellulose membranes in a Trans-Blot apparatus (Biorad). Membranes were blocked in TBSTw (Tris-buffered saline (150 mM NaCl, 20 mM Tris pH 7.5) + 0.05% Tween 20) and 5% (w/v) semi-skimmed milk for 1 h at room temperature and then incubated for 1 h with primary antibody in TBSTw. After washing three times for 5 min with TBSTw, the membranes were incubated in an appropriate horseradish peroxidase-linked secondary antibody (1 : 10 000) for 1 h in TBSTw at room temperature. Finally, the membranes were washed as above in Tris-buffered saline plus 0.2% Triton X and immune-complexes detected by enhanced chemiluminescence (ECL; GE Healthcare). Primary antibodies and dilutions used were as follows: anti- HA (Sigma, H3663) 1:2000; rabit anti-FKH (Abrams et al., 2006) (gift from Prof Deborah Andrew) 1:1000; anti-FLAG (Sigma, F3165) 1:2000. Secondary antibodies and dilutions used in were as follows: goat anti-rabbit (abcam, ab6721) 1:5000; goat anti-mouse (Abcam, ab6789) 1:5000.

Immunohistochemistry on adult guts

The following antibodies were used in in immunohistochemistry of guts; primary antibodies: rabbit anti-FKH (Abrams et al., 2006) (gift from Prof Deborah Andrew) 1:1000, rabbit anti-PH3 (Cell Signalling 9701) 1:500. Nile Red was diluted in PBS at 1:2000 from the stock solution prepared at 0.5mg/ml in acetone. Secondary antibodies: Alexa Fluor 488 donkey anti-rabbit (A21206) 1:1000. Guts were dissected in ice cold PBS. Insulin stimulation involved incubation of adult guts at room temperature in Schneider's medium (Sigma) containing 1 μM human insulin (Sigma). Guts were immediately fixed in 4% formaldehyde for 15 minutes, serially dehydrated in MeOH, stored at -20°C, and subsequently stained. Guts were washed in 0.2% 331 Triton-X / PBS, blocked in 5% bovine serum albumin / PBS, incubated in primary antibody overnight at 4°c and in secondary for 2 h at RT. 10-15 guts per condition were mounted, scored and imaged as described above.

Immunohistochemistry on S2 cells

The following antibodies were used in in immunohistochemistry of S2 cells; primary antibodies: mouse anti-HA (Sigma, H3663) 1:1000. Secondary antibodies: Alexa Fluor594 phalloidin (A12381) 1:1000; Alexa Fluor 488 donkey anti-rabbit (A21206) 1:1000. S2 cells were cultured on Concanavalin A coated coverslips in six-well plates overnight. Cells were rinsed in PBS for 3 min followed by a 3 min fixation in PBS containing 4 % paraformaldehyde (diluted from 16% ampoules). The fixative was removed by a 2 min wash in PBS. Cells were permeabilised by a 5 min incubation in PBS + 0.5% Triton X-100 followed by a 1 h block in PBS containing 3 per cent BSA. Subsequently, cells were washed in PBS + 0.1% Triton X-100 for 5 min. Primary antibody was diluted in PBS + 0.3% BSA + 0.1% Triton X-100 and incubated on the cells for 1 h at room temperature. Cells were washed twice for 5 min followed by a 1 h secondary antibody incubation diluted in PBS + 0.3% BSA + 0.1% Triton X-100. Subsequently, 45 min washes were performed in PBS + 0.1% Triton X-100. DAPI was included in the penultimate wash at 0.1 $\mu g\ ml^{-1}$ concentration.

Imaging

Images were captured using the Zeiss LSM 700 confocal microscope and processed using ImageJ software.

Smurf Assay

Gut barrier efficiency was analyzed by placing flies on blue food (minimum of 200 flies per condition at 10 week of age) prepared using 2.5% (w/v) FD&C blue dye no. 1 (Fastcolors) as previously described (Rera et al., 2012), except flies were kept 24 hours on the blue food before the Smurf phenotype was scored.

Generation of *Irs1* conditional KO and tissue-specific KO mice

Generation of *Irs1loxP/loxP* mice was described in Essers et al., 2016(Essers et al., 2016) For tissue-specific KO of *Irs1*, *Irs1loxP/loxP* mice were crossed with mice expressing Cre-recombinase under the control of the villin promoter (*Vill*Cre mice, The Jackson Laboratory, stock number 004586). Breeding *Irs1loxP/loxP Vill*Cre mice with *Irs1loxP/loxP*, produced mice with intestinal *Irs1* deletion (denoted as $VillCre::Irs1^{lox/lox}$) and littermate controls.

RNA isolation and quantitative PCR

RNA was isolated from 14 fly guts per sample or mouse small intestine with TRIzol (Invitrogen) and cDNA was synthesized by using SuperScript II Reverse Transcriptase (Invitrogen) according to manufacturers instructions. qPCR was carried out by using Power SYBR Green PCR Master Mix (ABI) on ABI Prism 7000.
The following primers sequences were used in the analysis.
Fork head (forward-ATTCGACATTCGCTGAGGTT, reverse-TCGAACATATTCCCCGAA)

Drosophila Actin5C (forward-CACACCAAATCTTACAAAATGTGTGA, reverse-AATCCGGCCTTGCACATG)
Zip42C.1 (forward- CTGTGGAACCTTGCTGTACG, reverse-AGGTTAGGCTGTCATCACCC)
Mdr50 (forward-GGCGCCAAACTAGAGGATTC, reverse-CGTACCGAAAGAGCTGGAAG)
Ctr1B (forward-ATCACGGCTCGGATGACAG, reverse-ACAGGAAGGACACCAGGAAG)
Pmp70 (forward-CCTCAGAGGCCCTACATGAC, reverse-CACGTCGATCCAGTCCTCAA)
CG1208 (forward- CCACAACTGGCGGTATTTCC, reverse- GGCGAAACTAAGCGTGATCC)
MtnB (forward-AAGGGTTGTGGAACAACTGC, reverse- GTCCTTGGGCCCATTCTT)
MtnC (forward-AAAGGCTGCGGAACAAACT, reverse-ATCTTTGGGGCCATTCTTG)
Abcd3 (forward-GGGAGAAGCAGACAATCCAC, reverse-CCGAAAGAAAATGAAATTATGTAGG)
Slc39a3 (forward-TGGCGTATTCCTGGCTACAT, reverse-GAAACCCACCATCATGAGCG)
Slc2a8 (forward- TCTTCATTGCTGGCTTTGCG, reverse-TTGGTGAGGACACAGATGCC)
Mouse Actin (forward- AACCGTGAAAAGATGACCCAG, reverse-CACAGCCTGGATGGCTACGTA

Expression and Purification of FKH

Full length, wild-type *Fork Head* coding sequence was cloned into pENTR-D-TOPO vector, transferred into pDEST17 expression vector and confirmed by sequencing. Rosetta™(DE3)pLysS Competent Cells (Novagen) were used to express the recombinant FKH. Bacterial cells were lysed via sonication in PBS+ 500 mM NaCl+ 25 mM immidizole containing Complete protease inhibitors (Roche) and lysates cleared by centrifugation. Purification of the protein was carried out on an Akta purifier system (GE Healthcare) and a Ni-NTA column. FKH was eluted with an imidazole gradient (25mM to 500 mM) in PBS + 500 mM NaCl. The fractions to be used for subsequent essays were determined by SDS-Page and Coomassie staining. Successful purification of recombinant FKH-his protein was confirmed by immunoblotting with an anti-His antibody (Sigma, H1029).

***In vitro* kinase reactions**

In vitro kinase reactions were carried out in a total volume of 30 µl containing 1X Kinase buffer (Cell signaling, 9802)+ 10 µM ATP + 5 µCi [γ-33P]ATP , 3µl of purified FKH and 0.1 µg of recombinant kinase. Active recombinant kinases mTOR kinase (SRP0364) and Akt1 (A8729) were purchased from Sigma and GSK fusion protein was purchased from Cell signaling (9237). Kinase reactions were incubated at 30°C for 20 minutes and reactions were stopped by adding sample buffer containing 10 mM DTT. Samples were subsequently boiled at 80°C for 10 minutes and run on 4–15% Mini-PROTEAN® TGX™ Precast Protein Gels (BioRad). The gel was dried on a Whatman 3.0 paper, and exposed to autoradiogram for 24 hours by using Amersham Hyperfilm (GE Healthcare).

Library preparation for sequencing

Fly guts were micro-dissected in ice-cold RNAlater (Qiagen) solution and RNA was extracted using the QIAGEN total RNA isolation kit and quantified on an Agilent 2100 bioanalyser. Sample concentration and purity of RNA was measured on a NanoDrop spectrophotometer, and RNA integrity was assessed on an Agilent 2100 Bioanalyzer. Samples were processed using Illumina's TruSeq Stranded mRNA LT sample preparation kit (p/n RS-122-2101) according to manufacturer's instructions. Deviations from the protocol were as follows:
250ng total RNA was used as starting material. Fragmentation was carried out for 10mins instead of 8mins, and 14 cycles of PCR were used.
Briefly, mRNA was isolated from total RNA using Oligo dT beads to pull down Poly-Adenylated transcripts. The purified mRNA was fragmented using chemical fragmentation (heat and divalent metal cation) and primed with random hexamers. Strand-specific first strand cDNA was generated using Reverse Transcriptase and Actinomycin D. This allows for RNA dependent synthesis while preventing spurious DNA-dependent synthesis. The second cDNA strand was synthesised using dUTP in place of dTTP, to mark the second strand.
The resultant cDNA is then "A-tailed" at the 3' end to prevent self-ligation and adapter dimerisation. Full length TruSeq adaptors, containing a T overhang are ligated to the A-Tailed cDNA. These adaptors contain sequences that allow the libraries to be uniquely identified by way of a 6bp Index sequence. Successfully ligated fragments were enriched with 14 cycles of PCR. The polymerase is

unable to read through uracil, so only the first strand is amplified, thus making the library strand-specific.

Sequencing

Libraries to be multiplexed in the same run were pooled in equimolar quantities, calculated from Qubit and Bioanalyser fragment analysis.

Samples were sequenced on the NextSeq 500 instrument (Illumina, San Diego, US) using a 43bp paired end run resulting in >15million reads per sample. Sequencing was carried out by UCL Genomics at the UCL GOS Institute of Child Health.

Alignment and differential expression of RNA-seq data

Initial quality control of the raw files (fastq) was performed using FastQC (v0.11.4, http://www.bioinformatics.babraham.ac.uk/projects/fastqc/). The raw RNA-seq data comprised paired-end reads with read length of 43bp. Reads were aligned to the *Drosophila* reference genome (Ensembl BDGP6 release 84 and *Wolbachia* NCBI NC_002978.6) using tophat2(Trapnell et al., 2009) (--library fr-firststrand --no-coverage-search --keep-fasta-order --segment-length 20 --segment-mismatches 1). Additionally, we estimated the tophat's insert distance (--mate-inner-dist) and standard deviation (--mate-std-dev) by aligning the first 1M reads. Aligned reads were filtered by removing non-primary alignments, unmapped reads and mates, reads not mapped in proper pairs and alignments with MAPQ <20, using samtools(Li et al., 2009) (v1.3.1; -F 0x100 -F 0x004 -F 0x008 -q 20 -f 0x002). Read counts per gene identifier were computed using htseq-count(Anders et al., 2015) (v0.6.0; --stranded=reverse -t exon --idattr=gene_id --mode=intersection-strict). Differentially expressed genes were determined using DESeq2(Love et al., 2014) (v1.10.1) with default parameters. Genes were deemed differentially expressed if p-value ≤ 0.05 following a Benjamini and Hochberg correction for multiple hypothesis testing (default parameter in DESeq2).

Functional annotation and gene-set enrichment analysis

The Wilcoxon rank sum test, as implemented in Catmap (Breslin et al., 2004), was used to perform functional analysis, ie significant enrichment of Gene Ontology (GO) categories. FlyBase's (http://flybase.org) gene identifiers were mapped to Gene Ontology identifiers (FlyBase version FB2016_04). Ranks of genes were based on the p-value derived from the DESeq2 analysis for differential expression. GO categories were deemed statistically significant if the p-value derived from the Wilcoxon rank sum test was ≤ 0.05 after Benjamini and Hochberg correction for multiple hypothesis testing. Gene lists were sorted by log-fold change and p-value. For all experiments three sets of lists were derived; a gene list comprising most differentially up-regulated (log-fold change > 0) genes at the top of the list and most differentially down-regulated genes (log-fold change < 0) at the bottom of the list (termed up-to-down) and vice versa (termed down-to-up). Additionally, a list comprising genes sorted by p-value only was also generated. These ranked lists of genes were used to generate significance of GO categories. If a GO category was found to be statistically significant in the up-to-down list, this GO was referred to as up-regulated, i.e. a large enough proportion of the genes that are part of this GO category were found to be up-regulated or at the top of the list. If a GO category was found to be statistically significant in the down-to-up list, this GO was referred to as down-regulated, i.e. a large enough proportion of the genes that are part of this GO category were found to be down-regulated or at the top of the list. GO categories were deemed to be statistically significant if the p-value was ≤ 0.05 after Benjamini and Hochberg correction for multiple hypothesis testing.

Statistical significance of differential expression gene sets

Statistical significance of overlaps of genes in two expression experiments was determined using Fisher's exact test. To account for multiple hypotheses testing, a p-value cut-off of ≤1.0x10-05 was used. Statistical significance of GO term enrichment in genes that overlapped between two experiments was determined using a hypergeometric test in R.

***Ecc15* infection**

Erwinia carotovora carotovora15 (Ecc15) was grown in LB overnight at 30 °C and overnight cultures were spun down and pelleted and resuspended in a very small volume of fresh LB. OD was measured at 600 nm and cultures were resuspended so that the final OD_{600} = 200. Flies were pricked with a needle dipped in the bacterial solution in the thorax just above the right wing. As a control same number of flies were pricked with a needle dipped in LB alone and survival was monitored over the following 7-8 days.

SUPPLEMENTAL REFERENCES

Abrams, E.W., Mihoulides, W.K., and Andrew, D.J. (2006). Fork head and Sage maintain a uniform and patent salivary gland lumen through regulation of two downstream target genes, PH4alphaSG1 and PH4alphaSG2. Development *133*, 3517-3527.
Anders, S., Pyl, P.T., and Huber, W. (2015). HTSeq--a Python framework to work with high-throughput sequencing data. Bioinformatics *31*, 166-169.
Bass, T.M., Grandison, R.C., Wong, R., Martinez, P., Partridge, L., and Piper, M.D. (2007). Optimization of dietary restriction protocols in Drosophila. J Gerontol A Biol Sci Med Sci *62*, 1071-1081.
Bischof, J., Maeda, R.K., Hediger, M., Karch, F., and Basler, K. (2007). An optimized transgenesis system for Drosophila using germ-line-specific phiC31 integrases. Proceedings of the National Academy of Sciences of the United States of America *104*, 3312-3317.
Breslin, T., Eden, P., and Krogh, M. (2004). Comparing functional annotation analyses with Catmap. BMC Bioinformatics *5*, 193.
Bulow, M.H., Aebersold, R., Pankratz, M.J., and Junger, M.A. (2010). The Drosophila FoxA ortholog Fork head regulates growth and gene expression downstream of Target of rapamycin. PloS one *5*, e15171.
Essers, P., Tain, L.S., Nespital, T., Goncalves, J., Froehlich, J., and Partridge, L. (2016). Reduced insulin/insulin-like growth factor signaling decreases translation in Drosophila and mice. Sci Rep *6*, 30290.
Li, H., Handsaker, B., Wysoker, A., Fennell, T., Ruan, J., Homer, N., Marth, G., Abecasis, G., Durbin, R., and Genome Project Data Processing, S. (2009). The Sequence Alignment/Map format and SAMtools. Bioinformatics *25*, 2078-2079.
Love, M.I., Huber, W., and Anders, S. (2014). Moderated estimation of fold change and dispersion for RNA-seq data with DESeq2. Genome Biol *15*, 550.
Poirier, L., Shane, A., Zheng, J., and Seroude, L. (2008). Characterization of the Drosophila gene-switch system in aging studies: a cautionary tale. Aging cell *7*, 758-770.
Rera, M., Clark, R.I., and Walker, D.W. (2012). Intestinal barrier dysfunction links metabolic and inflammatory markers of aging to death in Drosophila. Proceedings of the National Academy of Sciences of the United States of America *109*, 21528-21533.
Slack, C., Giannakou, M.E., Foley, A., Goss, M., and Partridge, L. (2011). dFOXO-independent effects of reduced insulin-like signaling in Drosophila. Aging cell *10*, 735-748.
Trapnell, C., Pachter, L., and Salzberg, S.L. (2009). TopHat: discovering splice junctions with RNA-Seq. Bioinformatics *25*, 1105-1111.
Tricoire, H., Battisti, V., Trannoy, S., Lasbleiz, C., Pret, A.M., and Monnier, V. (2009). The steroid hormone receptor EcR finely modulates Drosophila lifespan during adulthood in a sex-specific manner. Mechanisms of ageing and development *130*, 547-552.
