# Supplementary material for: Intestinal Fork Head Regulates Nutrient Absorption and Promotes Longevity
Source: Cell Rep. 2017 Oct 17;21(3):641–53. doi: 10.1016/j.celrep.2017.09.042 (PMC5656751; doi:10.1016/j.celrep.2017.09.042)
Supplement: Document S2. Article plus Supplemental Information [file mmc2.pdf]

Cell Reports

Article

Intestinal Fork Head Regulates Nutrient Absorption and Promotes Longevity

Graphical Abstract

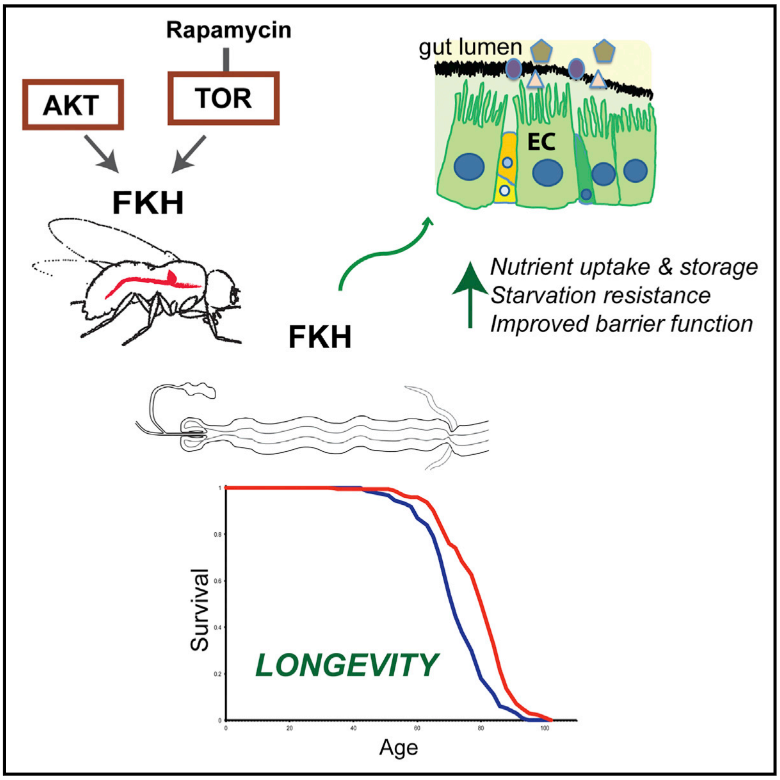

Highlights

- *Drosophila* FKH biochemically interacts with AKT and TOR
- IIS- and rapamycin-induced longevity requires FKH
- Gut tissue, specifically differentiated cells, mediates FKH's pro-longevity effects
- FKH activity in the gut upregulates intestinal nutrient transporters

Authors

Ekin Bolukbasi, Mobina Khericha, Jennifer C. Regan, ..., Janet M. Thornton, Nazif Alic, Linda Partridge

Correspondence

l.partridge@ucl.ac.uk

In Brief

Bolukbasi et al. identify the transcription factor FKH as a mediator of nutrient-sensing pathway signaling in *Drosophila*, and they characterize FKH's essential involvement in increased longevity via this network. They pinpoint FKH's pro-longevity effect to the gut, and they show increased expression of nutrient transporters and improvement of barrier function by FKH activity.

Data and Software Availability

E-MTAB-6056

Bolukbasi et al., 2017, Cell Reports *21*, 641–653
October 17, 2017 © 2017 The Author(s).
https://doi.org/10.1016/j.celrep.2017.09.042

OPEN ACCESS
CellPress

Intestinal Fork Head Regulates Nutrient Absorption and Promotes Longevity

Ekin Bolukbasi,[1,2] Mobina Khericha,[1,2] Jennifer C. Regan,[1,2] Dobril K. Ivanov,[3] Jennifer Adcott,[1] Miranda C. Dyson,[1] Tobias Nespital,[2] Janet M. Thornton,[3] Nazif Alic,[1] and Linda Partridge[1,2,4,*]
[1]Institute of Healthy Ageing, Department of Genetics, Evolution and Environment, University College London, Gower St, London WC1E 6BT, UK
[2]Max Planck Institute for Biology of Ageing, Joseph-Stelzmann-Strasse 9b, 50931 Cologne, Germany
[3]European Molecular Biology Laboratory, European Bioinformatics Institute (EMBL-EBI), Wellcome Trust Genome Campus, Hinxton, Cambridge CB10 1SD, UK
[4]Lead Contact
*Correspondence: l.partridge@ucl.ac.uk
https://doi.org/10.1016/j.celrep.2017.09.042

SUMMARY

Reduced activity of nutrient-sensing signaling networks can extend organismal lifespan, yet the underlying biology remains unclear. We show that the anti-aging effects of rapamycin and reduced intestinal insulin/insulin growth factor (IGF) signaling (IIS) require the *Drosophila* FoxA transcription factor homolog Fork Head (FKH). Intestinal FKH induction extends lifespan, highlighting a role for the gut. FKH binds to and is phosphorylated by AKT and Target of Rapamycin. Gut-specific FKH upregulation improves gut barrier function in aged flies. Additionally, it increases the expression of nutrient transporters, as does lowered IIS. Evolutionary conservation of this effect of lowered IIS is suggested by the upregulation of related nutrient transporters in *insulin receptor substrate 1* knockout mouse intestine. Our study highlights a critical role played by FKH in the gut in mediating anti-aging effects of reduced IIS. Malnutrition caused by poor intestinal absorption is a major problem in the elderly, and a better understanding of the mechanisms involved will have important therapeutic implications for human aging.

INTRODUCTION

The signaling network of nutrient-sensing, insulin/insulin growth factor signaling (IIS) and Target of Rapamycin (TOR) influences healthy lifespan in diverse eukaryotic organisms, including mammals (Alic and Partridge, 2011). Specific alleles of IIS genes (Li et al., 2009; Pawlikowska et al., 2009; Suh et al., 2008; Willcox et al., 2008) and transcriptional variation of genes encoding components of the TOR pathway (Passtoors et al., 2013) are associated with survival to advanced ages in humans. Reduced network activity can induce a broad-spectrum resistance to age-related loss of function and disease in animal models (Clancy et al., 2001; Kenyon et al., 1993; Selman et al., 2008; Tatar et al., 2001), making it an attractive target for pharmacological intervention to improve human health during aging (de Cabo et al., 2014). Indeed, attenuation of TOR signaling by rapamycin extends lifespan in diverse species, including mice (Bjedov et al., 2010; Harrison et al., 2009), as does inhibition of the Ras-Erk branch of IIS by the drug trametinib in *Drosophila* (Slack et al., 2015).

In addition to its effect on aging, the IIS/TOR network regulates growth, metabolism, stress responses, and fecundity, potentially resulting in undesired side effects of reduction of network activity. For example, at some doses, rapamycin is a strong immunosuppressant (de Cabo et al., 2014) and can also impair wound healing (Squarize et al., 2010), while trametinib is a Mek1/2 inhibitor with anti-proliferative properties (Yamaguchi et al., 2011). Therefore, we need to uncover molecular and mechanistic outputs of nutrient-sensing networks in order to triage apart the positive effects of intervention from the negative effects inherent in manipulating upstream network nodes. In particular, we need to determine the tissue-specific effect of signaling activity in lifespan extension and the physiological processes underlying it.

Recent studies identified the intestinal tissue as pivotal in aging (Alic et al., 2014; Biteau et al., 2010; Rera et al., 2012), and they have mainly focused on hyperplastic intestinal pathology resulting from age-dependent intestinal stem cell (ISC) over-proliferation as a major determinant of lifespan (Biteau et al., 2010). However, while stem cell maintenance is no doubt important for intestinal homeostasis, hyperplasia may not occur early enough to influence the early tipping point between young and old metabolic states. Therefore, other aspects of intestinal physiology that determine lifespan still remain to be elucidated.

Outputs of the IIS/TOR signaling network are mediated by several transcription factors (TFs). For instance, in *C. elegans* and *Drosophila*, the single Fork Head Box O (FoxO) TF is required for extended lifespan from lowered IIS (Giannakou et al., 2004; Hwangbo et al., 2004; Murphy et al., 2003; Slack et al., 2011; Willcox et al., 2008). In *C. elegans* the heat shock TF HSF-1 (Hsu et al., 2003) and the Nrf-like xenobiotic response factor SKN-1 (Tullet et al., 2008) are also required. In *Drosophila*, lowered IIS increases lifespan through both the canonical IIS pathway and its FOXO effector, and through the Ras-Erk-ETS branch and its transcriptional repressor effector *anterior open* (AOP) (Slack et al., 2015). In *Drosophila*, at least one other TF is also likely to play a role, because the TF-binding sites

This is an open access article under the CC BY license (http://creativecommons.org/licenses/by/4.0/).

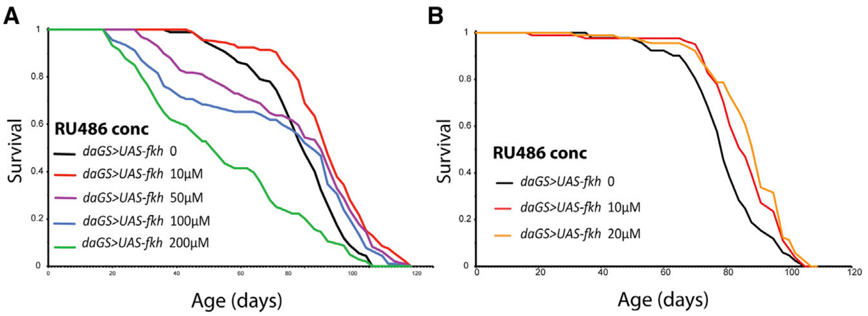

Figure 1. Ubiquitous Overexpression of FKH Extends Lifespan

(A and B) Survival curves of female flies expressing the *UAS-fkh* transgene under the control of the *daGS* driver at different RU486 concentrations. Ubiquitous overexpression of FKH extended lifespan (A) at 10 and 50 μM RU486 ($p = 6.94 \times 10^{-6}$ and p = 0.017, respectively) and (B) at 10 and 20 μM RU486 (p = 0.003 and $p = 6.33 \times 10^{-6}$, respectively). See also Table S1.

upstream of genes regulated by lowered IIS imply the involvement of a FKH box family member other than dFOXO (Alic et al., 2011).

The Fork Head box (FOX) family of TFs shares a conserved DNA-binding domain, whose sequence assigns them to subclasses from FoxA to FoxS (Lam et al., 2013). The *Drosophila* FoxA homolog FKH is the founding member and namesake, and it plays an essential role in embryonic development (Weigel et al., 1989). FKH also regulates larval cell size in a rapamycin- and TOR-dependent manner (Bülow et al., 2010). Mammalian FoxAs regulate glucose metabolism in the liver, pancreas, and adipose tissue (Friedman and Kaestner, 2006), and liver-specific knockout of FoxA2 results in a premature aging phenotype and increased mTOR activity in mouse (Bochkis et al., 2013).

We have investigated the role of *Drosophila* FKH in the IIS-and-TOR-signaling network and, in particular, its key role in intestinal aging. We find that FKH interacts with and is phosphorylated by both dAKT and dTOR, placing it as a central transcriptional regulator. Concordantly, we demonstrate an essential requirement for FKH for both IIS- and rapamycin-induced longevity, as well as IIS-induced starvation resistance, a phenotype previously shown to be dFOXO independent (Slack et al., 2011). We locate the longevity effects of FKH to intestinal tissue and specifically to differentiated intestinal cells. We establish that the anti-aging effects of rapamycin and intestinal IIS downregulation both require, and can be recapitulated by, FKH induction in the gut. Gut barrier function loss over aging is improved by intestinal FKH upregulation, while ISC proliferation remains unaffected. Transcriptomic analysis of adult guts revealed FKH-dependent upregulation of nutrient transporters upon reduced IIS. Consistent with this finding, we demonstrate an FKH-dependent increase in nutrient absorption upon reduced IIS and gut-specific FKH overexpression, suggesting improved gut absorption as a possible underlying longevity mechanism. Concordantly, starvation resistance declines over age, but it is rescued by intestinal FKH upregulation in young and old flies. Additionally, we show upregulation of related nutrient transporters in *irs1* knockout mouse intestine, suggesting evolutionary conservation of this mechanism. Overall, our results demonstrate FKH-dependent functional consequences of reduced IIS for intestinal absorption, and they imply that FoxA is an evolutionarily conserved regulator of lifespan and gut function, pointing to new directions for therapeutic intervention into aging-related loss of function.

RESULTS

FKH Overexpression Results in Increased Longevity

FOXO overexpression can increase longevity in both worms and flies (Alic and Partridge, 2011), and overexpression of the FoxA homolog *pha-4* can also do so in *C. elegans* (Panowski et al., 2007). To determine whether *Drosophila* FKH plays a similar role, we assessed the effect of ubiquitous, adult-onset FKH overexpression on lifespan.

We used the inducible daughterless Gene Switch (*daGS*) driver to produce a graded increase in FKH expression, by varying the concentration of the activating drug RU486. Strong FKH induction led to short-lived flies (Figure 1A), whereas weaker induction resulted in lifespan extension (Figure 1A; 10 μM median survival + 9%, $p = 6.94 \times 10^{-6}$). Intermediate induction at 50 and 100 μM RU486 resulted in early life mortality, and upregulation at 200 μM RU486 resulted in significantly short-lived flies. To more precisely define the range of induction that maximized lifespan, we used 10 and 20 μM RU486, and we found significant extension at both concentrations (Figure 1B; 10 μM RU486 median survival +9%, p = 0.003 and 20 μM median survival +14%, $p = 6.33 \times 10^{-6}$). Moderate, ubiquitous, adult-specific upregulation of FKH expression is thus sufficient to extend *Drosophila* lifespan in a dose-dependent manner.

Weakening immune response and hence lowered defense against infection contribute to decline of health during aging (Zerofsky et al., 2005). FKH was recently proposed to act as a regulator of innate immune response in *Drosophila* larvae (Varma et al., 2014). As an indicator of healthspan, we assessed the survival upon systemic infection with *Erwinia carotovora carotovora15* (*Ecc15*), a Gram-negative bacterium that has been used as a model of natural infection in *Drosophila* larvae and adults (Basset et al., 2000). Systemic *Ecc15* infection at 1 week of age resulted in lower mortality than did infection at 7 weeks of age (p = 0.00052), suggesting a decline in immune response over age. Overexpression of FKH led to increased survival at both ages (Figure S1; p = 0.00014 and p = 0.0001 for young and old cohorts, respectively). Hence, FKH activity results in more efficient immunity and attenuates age-related decline in immune function.

IIS Induces Phosphorylation and a Marked Decrease in Nuclear Localization of FKH

IIS activity results in phosphorylation and nuclear exclusion of dFOXO by *Drosophila* AKT (dAKT), and, thereby, it controls the expression of dFOXO target genes. We first determined whether

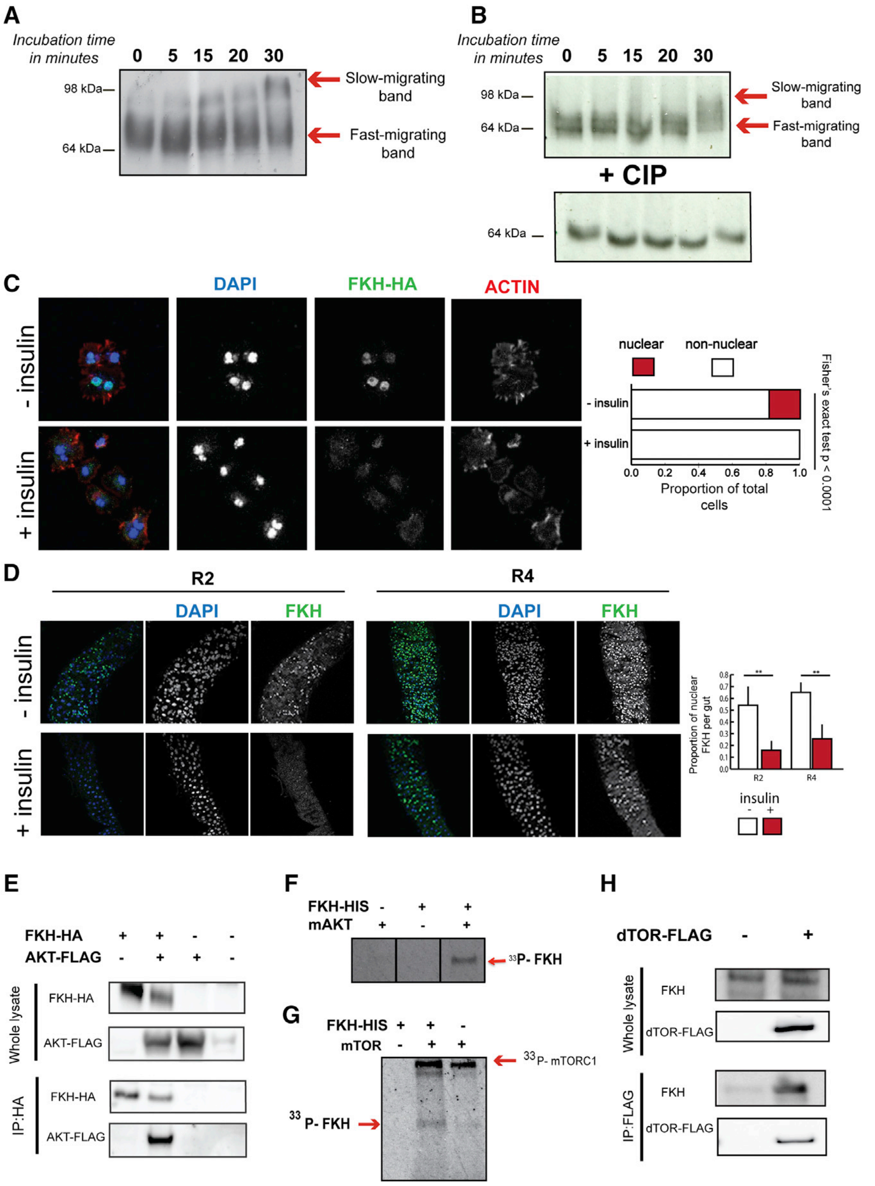

Figure 2. Activation of IIS Results in FKH Phosphorylation, and FKH Interacts with AKT and TOR In Vitro

(A) S2 cells were transfected with HA-tagged FKH construct and stimulated with insulin for 5, 15, 20, and 30 min. A band above 64 kDa was detected in extracts pre-insulin stimulation. A slower migrating band at ~98 kDa was present in extracts isolated post-insulin stimulation.

(B) Following phosphatase treatment of the same extracts, a single band corresponding to non-phosphorylated FKH-HA was present (lower panel).

(C) S2 cells were visualized for FKH-HA protein (green), Actin (red), and nuclei stained by DAPI (blue) pre-and post-insulin treatment for 20 min. Quantification of nuclear FKH revealed a significant difference between two conditions (Fisher's exact test, $p < 0.0001$).

(D) Ex vivo insulin-stimulated guts were stained for endogenous FKH (green) and DAPI-stained nuclei (blue) and in two different sections of midgut: R2 (left panel) and R4 (right panel). Initially strong nuclear FKH staining was markedly decreased in both R2 (t test, $p = 0.045$; $n = 4$) and R4 (t test, $p = 0.0014$; $n = 4$) upon insulin stimulation. Error bars represent SD.

(E) S2 cells were transiently transfected with the indicated cDNAs in expression vectors. Anti-HA immunoprecipitates were prepared, and cell lysates were analyzed by immunoblotting with either anti-HA or anti-FLAG antibodies.

(F and G) Recombinant FKH-HIS was incubated in an in vitro kinase reaction with active recombinant (F) Akt1 or (G) mTORC1 radiolabelled [γ-33P]ATP. Reactions lacking either the substrate or the kinase served as negative controls. Proteins were separated by SDS-PAGE and phosphorylated proteins were visualized by autoradiography. In reactions with mTORC1, the incorporation of [γ-33P]ATP was also observed at a higher molecular weight (~160 kDa), which probably reflected the previously reported phosphorylation of raptor by mTOR (Wang et al., 2009).

(H) S2 cells were transiently transfected with the dTOR-FLAG vector. Anti-FLAG immunoprecipitates were prepared, and cell lysates were analyzed by immunoblotting with either anti-FKH or anti-FLAG antibodies.

IIS activity has similar effects on FKH, by searching for the appearance of a slow-migrating FKH protein band on polyacrylamide gels (Puig et al., 2003). *Drosophila* S2 cells were transfected with an HA-tagged FKH construct, serum starved, and then stimulated with insulin, and protein extracts were run on polyacrylamide gels containing PhosTag (Kinoshita et al., 2006). Immunoblotting of unstimulated cell extracts revealed a band just above 64 kDa, while extracts from insulin-stimulated cells contained a slower migrating band of around 98 kDa, already apparent at 15 min post-insulin stimulation, with the highest intensity at 30 min (Figure 2A). Phosphatase treatment resulted in a single band at 64 kDa for all time points (Figure 2B), indicating that phosphorylation caused the appearance of the slow-migrating FKH band.

We next assessed if the subcellular localization of FKH was changed by IIS, using transiently overexpressed FKH-HA protein and immunofluorescence of S2 cells pre- and post-stimulation with insulin. Around 20% of unstimulated transfected cells showed strong nuclear staining of HA-tagged FKH protein, which was lost upon stimulation with insulin (Figure 2C). To determine whether insulin affected localization of endogenous FKH in adult tissue, guts were isolated from 5-day-old flies and stimulated with insulin ex vivo for 20 min. The *Drosophila* gut is regionalized in morphology and function (Buchon et al., 2013), and we measured FKH localization in two regions of the midgut, R2 and R4. In both, insulin stimulation led to reduced nuclear FKH staining (Figure 2D).

IIS activity thus results in phosphorylation of FKH in cell culture and a substantial decrease in nuclear localization of FKH in both

cell culture and gut ex vivo, demonstrating IIS regulation of FKH function.

FKH Interacts with AKT and TOR In Vitro

We next determined where FKH is integrated into the IIS/TOR network. Since mammalian FoxA2 interacts with mAKT1 in vitro (Wolfrum et al., 2003) and FKH genetically interacts with dTOR in *Drosophila* larval fat body (Bülow et al., 2010), we investigated AKT and TOR kinases as candidates for a potential interaction with FKH.

Drosophila S2 cells were transiently transfected with HA-tagged FKH and/or a FLAG-tagged dAKT. Following 15 min of insulin stimulation, FKH-HA protein was co-immunoprecipitated as part of a complex with FLAG-tagged dAKT (Figure 2E). Since FKH could physically interact with dAKT, we next assessed whether AKT could phosphorylate FKH. Recombinant, His-tagged FKH was expressed in *E.coli* and purified. An in vitro kinase assay was carried out using mammalian recombinant active mAKT, and phosphorylation of FKH was assessed by the incorporation of [γ-33P]ATP. FKH-His was phosphorylated upon incubation with active mAKT (Figure 2F).

To assess whether FKH interacted physically with dTOR, S2 cells were transfected with FLAG-tagged dTOR, which was co-immunoprecipitated with endogenous FKH protein (Figure 2H). An in vitro kinase assay with recombinant active mTORC1 and purified FKH-His resulted in the phosphorylation of FKH (Figure 2G).

FKH can interact physically with both AKT and TOR and can be phosphorylated by them, and it could thus integrate signals from these two kinases.

FKH Functions Downstream of IIS to Determine Lifespan

In *Drosophila,* the reduction of IIS in adult flies results in a complex array of phenotypes, including resistance to starvation, oxidative stress and xenobiotics, reduced fecundity, and increased longevity (Slack et al., 2011).

To determine the contribution of FKH function to longevity, we knocked down *fkh* using RNAi in wild-type flies and flies with a dominant-negative version of the single-fly IIS receptor InR^{DN}. Assessment of transcript levels by qPCR showed that ubiquitous RNAi against *fkh* resulted in an ~65% reduction in *fkh* expression (Figure S2A). Ubiquitous, adult-specific expression of InR^{DN} with the inducible *daGS* driver resulted in a significant lifespan extension in flies with co-overexpression of *GFP RNAi*, to control for possible titration of GAL4 (Figure 3A; median survival +14%, $p = 4.45 \times 10^{-10}$). However, RNAi against *fkh* both substantially reduced the lifespan of wild-type flies and blocked the extension of lifespan by InR^{DN} (Figure 3B; p = 0.08). Cox Proportional Hazard (CPH) analysis established that the response to the presence of the activating drug RU486 in *daGS;UAS-*InR^{DN} flies was significantly different in the presence and absence of *fkh* RNAi (Table S3; p = 0.0432). To confirm that IIS was biochemically active upon FKH RNAi, we assessed expression levels of the dFOXO target *4EBP*, and we found a significant increase upon reduced IIS on both wild-type and FKH knockdown background (Figure S2E; t test, $p < 0.05$).

We examined the role of FKH in the extension of lifespan by a second IIS mutant, heterozygous $chico^1$, which has lost a single copy of the insulin receptor substrate and is long-lived (Clancy et al., 2001). $chico^1$ heterozygotes were long-lived with control GFP RNAi (Figure 3C; median survival +8%, $p = 2.71 \times 10^{-6}$), but they lost this lifespan extension with RNAi against *fkh* (Figure 3D; p = 0.61), confirmed by CPH analysis (Table S3; p = 0.00452). To confirm the specificity of the effect of *fkh* RNAi, we used a second *fkh* RNAi line (Bülow et al., 2010) (referred to as *UAS-fkh RNAi 2)* that also prevented lifespan extension upon overexpression of *UAS-*InR^{DN} and heterozygous $chico^1$ mutation (Figures S2B–S2D).

These data show that FKH function is essential for mediating the lifespan-extending effects of reduced IIS in *Drosophila*.

FKH Mediates Lifespan Extension by Rapamycin, but Not by Dietary Restriction

TOR is a conserved serine/threonine kinase that is present as two distinct protein complexes, TORC1 and TORC2. TORC1 activity can be directly inhibited by rapamycin (Cornu et al., 2013; Kapahi et al., 2004), which can also extend lifespan in yeast (Powers et al., 2006), *C. elegans* (Robida-Stubbs et al., 2012), *Drosophila* (Bjedov et al., 2010), and mice (Harrison et al., 2009). Our results showed biochemical interaction between FKH and TOR, and we therefore examined whether FKH is required for rapamycin-induced longevity.

Treatment of control flies with 100 μM rapamycin resulted in significant lifespan extension (Figure 3E; median survival +6.5%, $p = 2.37 \times 10^{-8}$), which was abolished by ubiquitous, adult-specific RNAi against *fkh* (Figure 3E; p = 0.81), confirmed by CPH analysis (Table S3; $p = 5.5 \times 10^{-4}$). The biochemical effect of rapamycin upon FKH RNAi was confirmed by assessing phosphorylation levels of S6K, which were decreased on both wild-type and FKH knockdown backgrounds following rapamycin treatment (Figure S2F). FKH thus functions downstream of TOR to mediate the extension of lifespan by rapamycin.

TOR kinase can play a role in the extension of lifespan by dietary restriction (DR) (Grandison et al., 2009), and the *C. elegans* homolog of *fkh* is required for DR-induced longevity (Panowski et al., 2007). We subjected flies overexpressing *fkh* RNAi and uninduced controls to DR, and we found that DR promoted longevity in both (Figures S3A and S3B; at 0.5× versus 1× yeast concentration, for +RU486 condition median survival +18%, $p = 1.02 \times 10^{-10}$, and for −RU486 condition median survival +3%, p = 0.03, respectively). Importantly, since ubiquitous RNAi again *fkh* shortened lifespan, the extension of lifespan in these flies by DR indicates that the failure to extend in response to reduced IIS and rapamycin was specific to these interventions.

Together these results indicate that FKH is required for rapamycin-induced, but not DR-induced, longevity.

FKH Is Required for IIS-Induced Starvation Resistance

In addition to its effect on lifespan, lowered IIS is associated with resistance to starvation, oxidative stress, and xenobiotics. We therefore determined whether these types of stress resistance require FKH function.

Flies were subjected to oxidative stress by supplementing their food with paraquat, a superoxide generator. Flies expressing InR^{DN} were resistant to paraquat (Figure 4A; p = 0.00058).

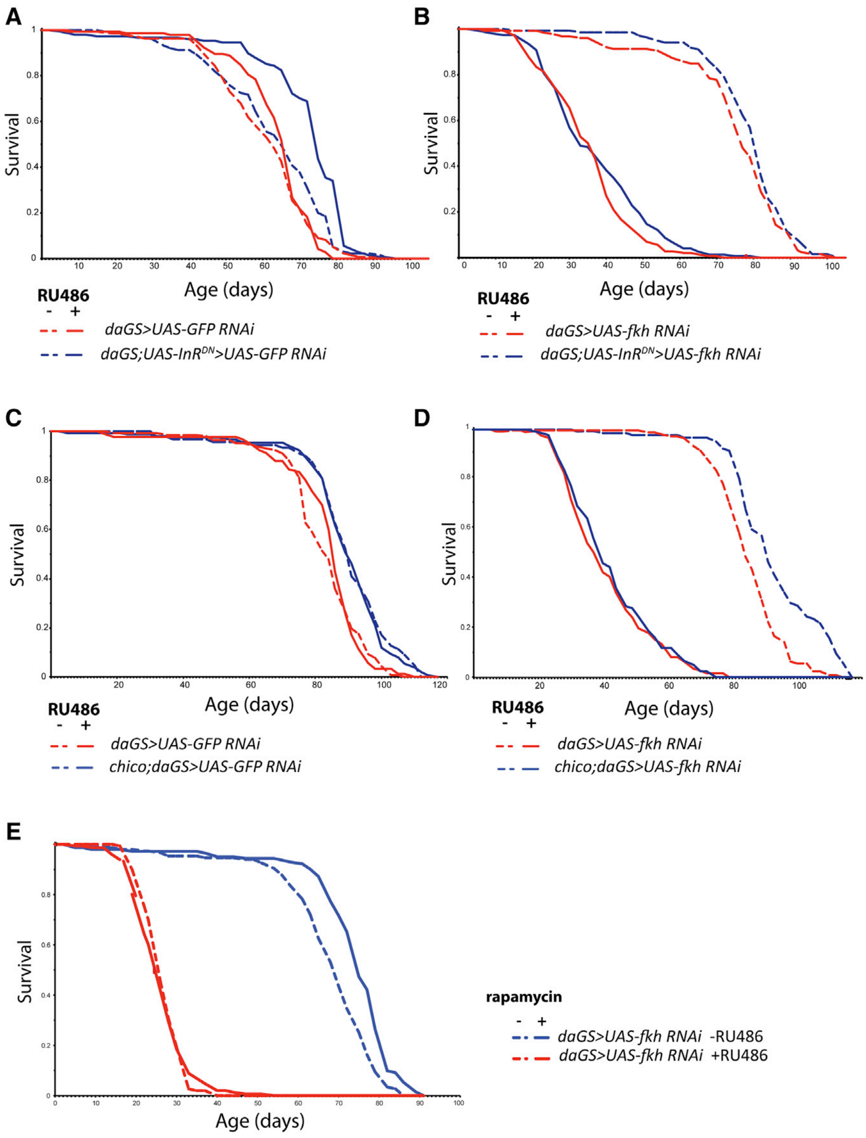

Figure 3. FKH Is Required for Reduced IIS- and Rapamycin-Induced Longevity

(A) *daGS;UAS-InR^{DN} > UAS-GFP RNAi* females showed increased lifespan in the presence of RU486 ($p = 4.45 \times 10^{-10}$). Survival of *daGS > UAS-GFP RNAi* females was not different between –RU486 and +RU468 conditions (p = 0.508). See also Table S2A.

(B) *daGS > UAS-fkh RNAi* and *daGS;UAS-InR^{DN} > UAS-fkh RNAi* flies showed significantly decreased lifespan in the presence of RU486 ($p = 1.97 \times 10^{-54}$ and $p = 2.59 \times 10^{-63}$, respectively). No significant difference in survival was detected between *daGS > UAS-fkh RNAi* +RU486 and *daGS;UAS-InR^{DN} > UAS-fkh RNAi* +RU486 flies (p = 0.08). See also Table S2B.

(C) *chico* mutation increased lifespan of *daGS>UAS-GFP* RNAi females in the absence ($p = 6.28 \times 10^{-7}$) and in the presence ($p = 2.71 \times 10^{-6}$) of RU486. See also Table S2C.

(D) *chico* mutation extended lifespan in *daGS > UAS-fkh RNAi* female flies in the absence of RU486 ($p = 3.86 \times 10^{-8}$), but not in its presence (p = 0.61). See also Table S2D.

(E) Rapamycin extended the lifespan of *daGS > UAS-fkh RNAi* female flies in the absence of RU486 ($p = 2.37 \times 10^{-8}$), but not in its presence (p = 0.81). See also Table S2E.

Ubiquitous RNAi against GFP or *fkh* did not affect the resistance of either wild-type (Figure S4A) or *InR^{DN}*-expressing flies (Figure 4A; Table S3). Resistance to xenobiotic treatment was tested by supplementing fly food with dichlorodiphenyltrichloroethane (DDT). Ubiquitous RNAi against GFP or *fkh* did not affect resistance of wild-type flies to DDT (Figure S4B). Reduction of IIS by *InR^{DN}* expression resulted in a small, but significant, increase in resistance (Figure 4B; p = 0.0013), but flies lacking *fkh* function showed even greater resistance to DDT upon reduction of IIS (Figure 4B; p = 0.00028). FKH function is thus not required for reduced IIS to induce resistance to oxidative stress or xenobiotic treatment, and indeed somehow it interferes with the latter.

Resistance to starvation was assessed by exposing flies only to agar. Ubiquitous RNAi against GFP or *fkh* did not affect the response to starvation (Figure S4C). Flies with reduced IIS were resistant to starvation (Figure 4C; $p = 3.66 \times 10^{-5}$). Knocking down *fkh* resulted in a loss of starvation resistance in flies expressing *InR^{DN}* (Figure 4C; p = 0.085), with CPH revealing a significant difference between these conditions (Table S3; $p = 4 \times 10^{-5}$). Resistance to starvation upon the downregulation of IIS thus requires FKH function.

IIS mutant females often have a fecundity deficit (Slack et al., 2011), and we assessed the role of FKH in this trait. RNAi against GFP did not affect egg-laying (Figure 4D; p = 0.47). Reduction of IIS resulted in a substantial decrease in fecundity (Figure 4D; $p = 2.04 \times 10^{-7}$), as did *fkh* RNAi alone (Figure 4D; $p = 1.63 \times 10^{-5}$). Knocking down *fkh* in flies with lowered IIS neither restored nor exacerbated their fecundity deficit. These results suggest that FKH is not required for the reduced fecundity observed upon the downregulation of IIS. The decrease in fecundity observed upon reduced IIS and *fkh* RNAi alone was not additive, and, therefore, it was likely to be occurring through independent mechanisms.

Upregulation of FKH in the Gut Extends Lifespan and Improves Gut Barrier Function

Many targets of TFs are cell type specific (Heintzman et al., 2009). Indeed, both dFOXO (Alic et al., 2014; Teleman et al., 2008) and AOP (Alic et al., 2014) have distinct targets in different tissues. Interestingly, *dfoxo* or *aop* activity in the abdominal fat body and adult midgut results in longevity, whereas their upregulation in the gut alone does not (Alic et al., 2014). A possible reason for the requirement of several distinct TFs

OPEN ACCESS
CellPress

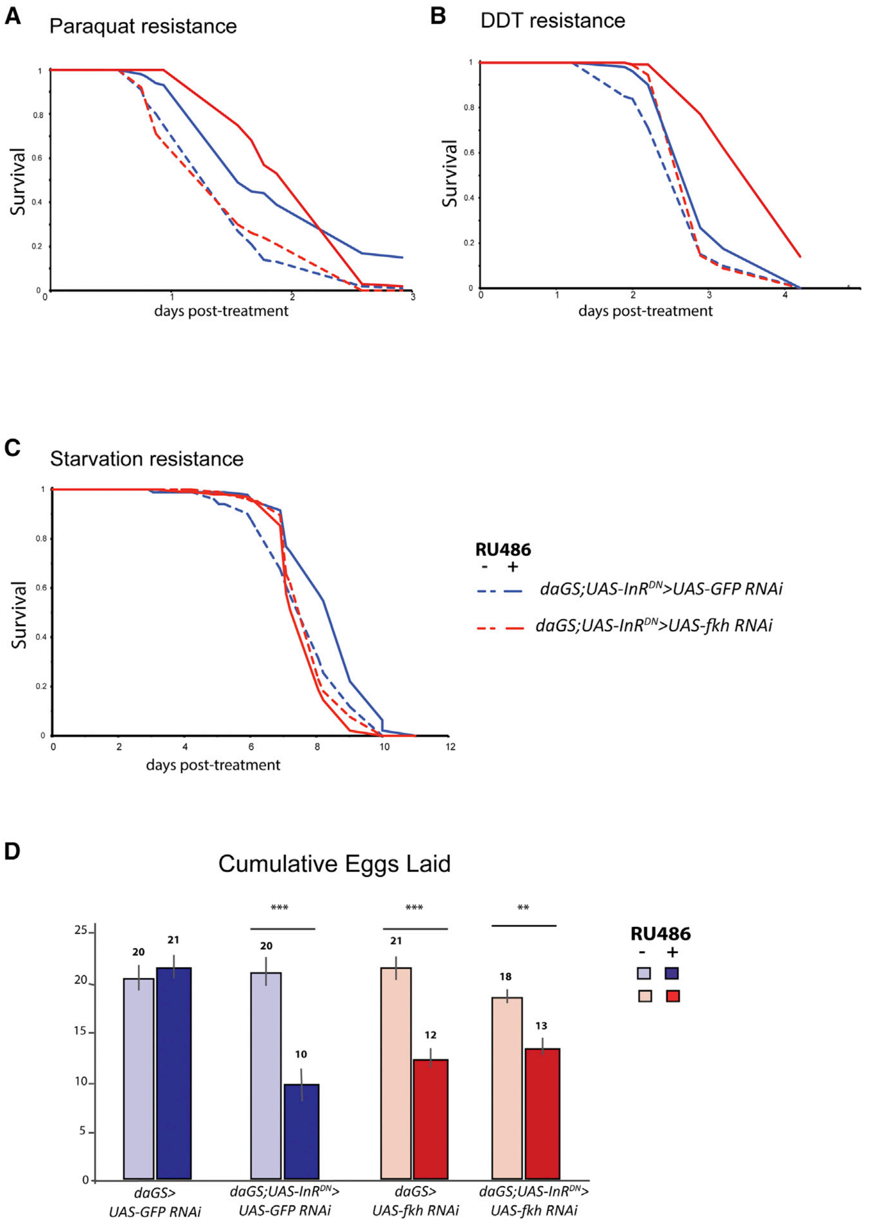

Figure 4. Functions of FKH during Lowered IIS-Induced Oxidative Stress, Xenobiotics, Starvation Resistance, and Reduced Fecundity

(A and B) The (A) paraquat and (B) DDT resistance of *daGS;UAS-InR^{DN} > UAS-GFP RNAi* and *daGS;UAS-InR^{DN} > UAS-fkh RNAi* females was significantly increased in the presence of RU486 ($p = 0.00058$ and $p = 1.46 \times 10^{-7}$; $p = 0.0013$ and $p = 3.58 \times 10^{-14}$, respectively). See also Table S4, A and B.

(C) Starvation resistance of *daGS;UAS-InR^{DN} > UAS-GFP RNAi* females was significantly increased in the presence of RU486 ($p = 3.66 \times 10^{-5}$), but resistance to starvation was not different for *daGS;UAS-InR^{DN} > UAS-fkh RNAi* in the presence of RU486 ($p = 0.085$). See also Table S4C.

(D) Cumulative number of eggs per female over 3 weeks following RU486 induction. Data are presented as the mean number of eggs laid per female over a 24-hr period each week ± SEM; t test revealed a significant difference between +RU486 and −RU486 conditions for *daGS;UAS-InR^{DN} > UAS-GFP RNAi* ($p = 2.04 \times 10^{-7}$), *daGS;UAS-InR^{DN} > UAS-fkh RNAi* ($p = 0.0037$), and *daGS > UAS-fkh RNAi* ($p = 1.63 \times 10^{-5}$).

for IIS-mediated lifespan is, thus, the tissue specificity of their action. We therefore investigated possible tissue specificity of FKH action to affect lifespan. A plethora of studies in both *C. elegans* (Libina et al., 2003; Tullet et al., 2008) and *Drosophila* (Alic et al., 2014; Biteau et al., 2010; Regan et al., 2016; Slack et al., 2015) has pointed to adipose and/or intestinal tissue as key mediators of the effects of reduced IIS on lifespan, and we therefore investigated the role of FKH in these two tissues.

Overexpression of FKH in the adult gut using the inducible TiGS driver, which expresses throughout the gut including differentiated cells (Alic et al., 2014), significantly increased lifespan (Figure 5A; median survival +6%, $p = 0.00015$), but its overexpression simultaneously in abdominal fat body and gut with *$S_1$106* driver did not (Figure S5A; $p = 0.093$). Furthermore, FKH overexpression restricted solely to ISCs and enteroblasts (EBs) with *GS5961* driver (Biteau et al., 2010) did not affect longevity (Figure S5B; $p = 0.21$). RU486 feeding had no effect on the lifespan of control flies with the UAS transgene or driver alone (Figures S5D–S5H).

These results show that at least part of the lifespan-extending effect of FKH activity occurs in the gut, in contrast to dFOXO. However, FKH upregulation solely in the progenitor cells (ISCs and EBs) is not sufficient to extend lifespan, suggesting involvement of other gut cell types.

To determine how FKH acts in the gut to extend lifespan, we examined the gut barrier function by feeding aged flies with a blue dye that does not normally leak into the body cavity (Rera et al., 2012), and we scored the proportion of “Smurf” flies, in which leakage occurred. FKH overexpression resulted in a significant decrease in the number of Smurfs at 10 week of age (Figure 5B; Fisher’s exact test, $p < 0.0001$), indicating stronger gut barrier function.

ISC proliferation increases with age, eventually leading to intestinal dysplasia (Biteau et al., 2008; Choi et al., 2008). To determine whether FKH function affects age-related proliferation of ISCs, we assessed the number of PH3-positive cells in the gut, but we did not find any difference at either young or old age between FKH-overexpressing and control flies (Figure S5C; t test, $p = 0.42$ and $p = 0.51$).

OPEN
ACCESS
CellPress

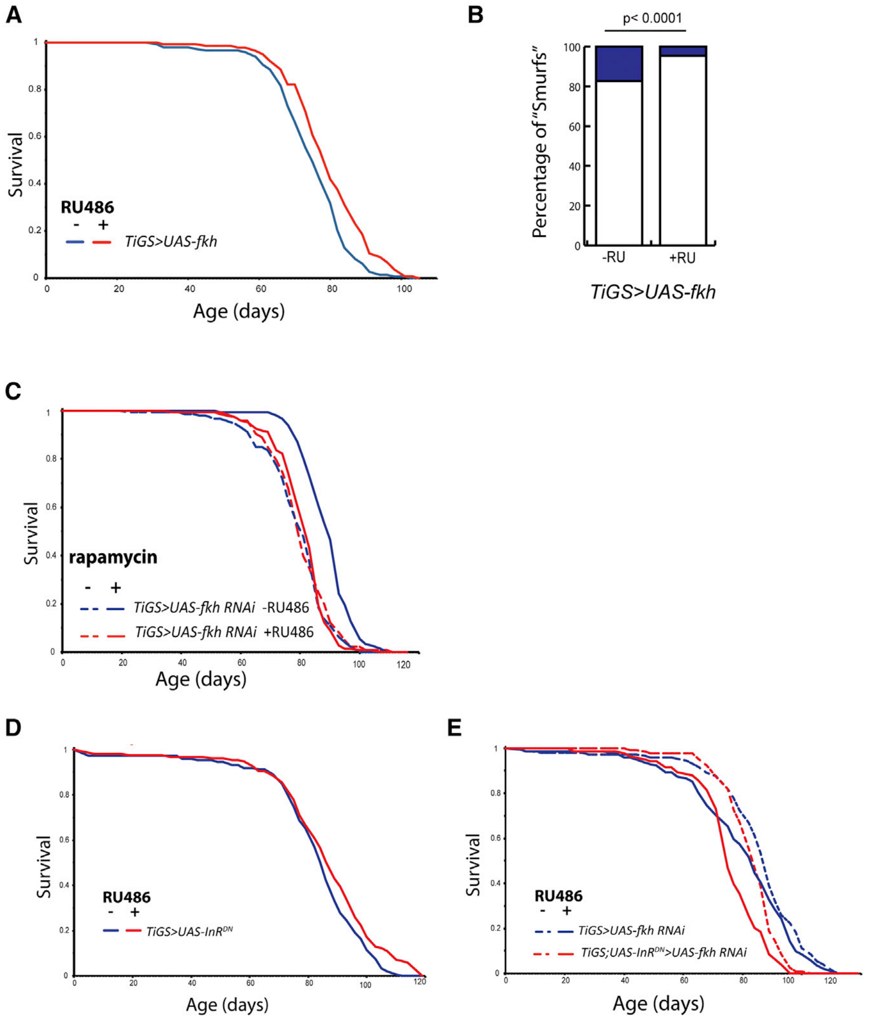

Figure 5. FKH Upregulation in the Gut Extends Lifespan, and Intestinal FKH Function Mediates Longevity Effects of Rapamycin and Reduced IIS

(A) Adult gut-specific overexpression of *UAS-fkh* under the control of TiGS driver extended lifespan (p = 0.00015). See also Table S5A.

(B) Quantification of the proportion of "Smurf" flies with leaky guts at 10 weeks of age in *TiGS>UAS-fkh* flies revealed a significant reduction in Smurf numbers in the +RU486 condition (two-tailed Fisher's exact test, $p < 0.0001$; n > 200 flies per condition).

(C) Rapamycin extended the lifespan of *TiGS > UAS-fkh RNAi* females in the absence of RU486 ($p = 1.79 \times 10^{-12}$), but not in its presence (p = 0.57). See also Table S5B.

(D) *TiGS < UAS-InR^DN* females were long-lived in the presence of RU486 (p = 0.014). See also Table S5C.

(E) Survival of *TiGS > UAS-fkh RNAi* females was not affected by RU468 (p = 0.077). The reduction of IIS upon overexpression of *UAS-fkh RNAi* led to significantly short-lived flies ($p = 8.97 \times 10^{-7}$). See also Table S5, D and E.

These results suggest that FKH overexpression in the gut maintains barrier function during aging, but it does not affect ISC proliferation.

FKH Function in the Gut Mediates Rapamycin-Induced Longevity

Rapamycin reduces age-related intestinal pathologies in flies (Fan et al., 2015). Given the increased lifespan upon gut-targeted FKH overexpression, we assessed the requirement for FKH for rapamycin-induced longevity. We knocked down *fkh* in the gut by RNAi with the *TiGS* driver. Treatment of uninduced flies with rapamycin significantly extended lifespan (Figure 5C; median survival + 11%, $p = 1.79 \times 10^{-12}$), and this extension was lost upon gut-specific RNAi against FKH (Figure 5C; p = 0.57), confirmed by CPH analysis (Table S3; $p = 6.66 \times 10^{-15}$). Hence, FKH function in the gut is necessary for rapamycin-induced longevity.

Reduced IIS in the Gut Induced an FKH-Dependent Extension of Lifespan

Downregulating IIS activity in progenitor cells of the gut extends *Drosophila* lifespan (Biteau et al., 2010). We examined the effect of a broader IIS reduction, and the contribution of FKH, by expressing InR^{DN} and RNAi against *fkh* under the control of the *TiGS* driver. The gut-targeted reduction of IIS resulted in a small, but significant, increase in lifespan (Figure 5D; median survival + 3%, p = 0.014), while RNAi against *fkh* resulted in slightly elevated early life mortality (Figure 5E; p = 0.077). Importantly, a reduction of IIS in the gut in combination with *fkh* knockdown failed to extend lifespan, and indeed it led to significantly short-lived flies (Figure 5E; $p = 8.92 \times 10^{-7}$). Hence, the decrease of IIS activity in the gut alone results in long-lived flies, and this not only requires FKH function but also is deleterious without it.

Reduced IIS Increases the Expression of Intestinal Transmembrane Transporters through FKH, and It Induces Enterocyte-Specific Nuclear Localization of FKH

Our results indicate that FKH function in the gut is necessary for both rapamycin- and reduced IIS-induced longevity. Given the detrimental effect of FKH knockdown in guts with reduced IIS, in our subsequent analysis we focused on the identification of genes regulated by FKH in the gut upon downregulation of IIS. We isolated guts from flies with ubiquitous reduction of IIS with or without knockdown of FKH, and controls, and we carried out genome-wide transcript profiling using next-generation RNA sequencing.

We first identified the set of genes differentially expressed in wild-type and reduced-IIS guts (*daGS;UAS-InR^DN* > *UAS-GFP*

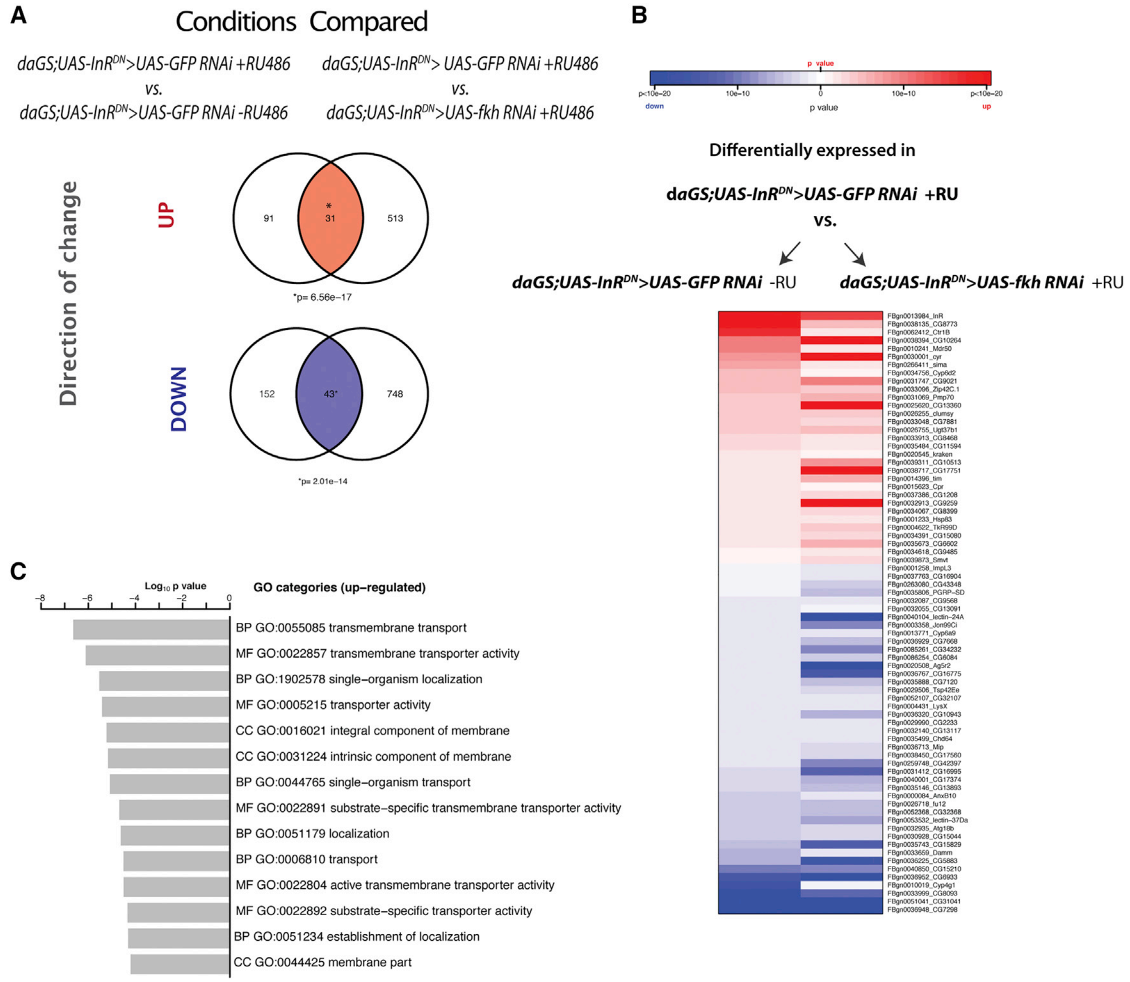

Figure 6. Reduced IIS in the Gut Results in a Transcriptional Response that Upregulates Transmembrane Transporters via FKH Function

(A) In the adult gut, the overlap between the transcriptional response induced by reduced IIS compared to controls (*daGS;UAS-InR*^{DN} > *UAS-GFP RNAi* +RU486 versus *daGS;UAS-InR*^{DN} > *UAS- GFP RNAi* –RU486) and compared to flies with reduced IIS and *fkh* knockdown (*daGS;UAS-InR*^{DN} > *UAS-GFP RNAi* +RU486 versus *daGS;UAS-InR*^{DN} > *UAS-fkh RNAi* +RU486) revealed 31 upregulated and 43 downregulated genes.

(B) Heatmap showing genes significantly upregulated or downregulated in both comparisons.

(C) Significantly upregulated GO categories induced by reduced IIS in an FKH-dependent manner in the adult gut.

RNAi + RU486 versus *daGS;UAS-InR*^{DN} > *UAS-GFP RNAi* – RU486) and the set of genes differentially expressed in reduced-IIS guts with or without RNAi against FKH (*daGS;UAS-InR*^{DN} > *UAS-GFP RNAi* + RU486 versus *daGS;UAS-InR*^{DN} > *UAS-fkh RNAi* + RU486). We reasoned that the transcriptional response regulated by FKH in response to reduced IIS should be indicated by genes whose expression levels are reverted back to wild-type levels upon FKH knockdown in reduced-IIS flies (Figures 6A and 6B). We carried out gene ontology (GO) category analysis on genes in the overlap between these two sets.

We found GO enrichment only among genes upregulated in reduced-IIS flies, particularly for genes encoding proteins involved in transmembrane transport (Figure 6C). Within this GO category were a number of genes responsible for the transport and uptake of nutrients, such as copper and zinc (*ctr1b* and *zip1*), glucose (*CG1208*), phospholipids (*mdr50*), and fatty acids (*pmp70*) (Figure 6B), suggesting that FKH could be increasing nutrient uptake and absorption in reduced-IIS flies.

Drosophila enterocytes (ECs) regulate nutrient transport and absorption in the midgut (Lemaitre and Miguel-Aliaga, 2013). We next investigated whether reduced IIS affected subcellular FKH localization, and we found that it increased nuclear localization of FKH specifically in ECs, which are characterized by polyploidy and large nuclei (Figure S6; t test, $p < 0.0001$). In cells with smaller nuclei, possibly consisting of ISCs, EBs, and enteroendocrine cells (EEs), nuclear localization of FKH did not change (Figure S6; t test, $p = 0.78$). Additionally, and in agreement with previously published work (Biteau et al., 2010), in the IIS mutant there were fewer cells with smaller nuclei, consistent with reduced ISC proliferation. These observations suggest a role for FKH in regulating transcription in ECs, including of genes related to nutrient transport and absorption, upon reduced IIS activity.

FKH Function Promotes Nutrient Absorption, and FKH Activity in Differentiated Gut Cells Results in Increased Longevity

To confirm our transcriptomic data, we assessed by qRT-PCR the expression levels of transmembrane transporters, including *ctr1B*, *zip1*, *mdr50*, *pmp70*, and *CG1208* (Figure S7A), and we found that they were increased in the IIS mutant (t test, p = 0. 0029, p = 0.0035, p = 0.031, p = 0.043, and p = 0.03, respectively; n = 3–4). RNAi against *fkh* abolished the differential expression of *ctr1B*, *mdr50*, *pmp70*, or *CG1208* (t test, p > 0.05; n = 3–4), but *zip1* expression levels remained significantly increased (t test, p = 0.0085; n = 4). Linear model analysis showed a significant interaction between RU and genotype (p = 0.0004), showing that the expression of genes involved in transport of nutrients was increased in the IIS mutant in an FKH-dependent manner.

We next assessed the effect of gut-specific upregulation of FKH on the expression of nutrient transporters, and we found a significant increase in *ctr1B*, *zip1*, and *mdr50* expression in guts with the induction of *fkh* (Figure S7B; t test, p = 0.02, p = 0.006, and p = 0.007, respectively). Therefore, FKH overexpression in the gut alone can upregulate expression of nutrient transporters.

To determine whether the upregulation of nutrient transporters also increased nutrient uptake, we assessed intestinal metal and lipid absorption. *Drosophila* metallothioneins are cysteine-rich proteins that bind heavy metals, such as zinc and copper, and their expression levels are used as a proxy measure of metal content of the *Drosophila* gut (Binks et al., 2010; Qin et al., 2013). We assessed by qRT-PCR the expression levels of the *metallothioneinB* (*mtnB*) and *metallothioneinC* (*mtnC*) in guts isolated from flies with *fkh* knockdown alone or with reduced IIS with or without *fkh* knockdown. Reduced IIS increased expression of both metallothionein genes, and this increase was dependent on FKH (Figure S7C), as confirmed by linear model analysis (interaction between RU and genotype, p = 0.0015). Next, we quantified *mtnB* and *mtnC* expression in guts with intestinal FKH overexpression and controls at 1 and 7 weeks of age. *mtnC* levels were significantly upregulated in *fkh*-overexpressing guts at both ages (Figure S7D; t test, p = 0.02 and p = 0.005), whereas *mtnB* levels were significantly increased only at 7 weeks of age (Figure S7D; t test, p = 0.01). These results indicate that FKH promotes intestinal metal uptake in the intestine upon reduced IIS activity.

Previous studies highlighted a key role for the fly gut in absorption and metabolism of dietary lipids (Karpac et al., 2013; Luis et al., 2016). The midgut displays an age-related decline in lipid storage, probably due to an overall reduction in intestinal lipid absorption, which results in increased starvation sensitivity (Karpac et al., 2013). We assessed intestinal lipid uptake with Nile Red staining to visualize lipid droplets in midguts with reduced IIS and with or without *fkh* knockdown. In agreement with previously published work, we detected lipid droplets in the anterior region of the midgut (Karpac et al., 2013; Luis et al., 2016), with a significant increase upon reduced IIS (Figures 7A and S7E; t test, p = 0.0019). This was lost upon *fkh* knockdown specifically in the guts of reduced-IIS flies (Figures 7A and S7E; t test, p = 0.094). These results suggest that FKH promotes intestinal lipid storage in response to reduced IIS. Gut-specific FKH overexpression also increased Nile Red staining in the midgut (Figures 7B and S7F; t test, $p = 2.29 \times 10^{-8}$), TAG levels in whole flies (Figure 7C; t test, p = 0.033), and resistance to starvation in both young and old flies (Figure 7D; p = 0.0047 and $p = 1.74 \times 10^{-5}$).

Our findings suggest a role for FKH in nutrient absorption. We next assessed whether increasing FKH activity specifically in differentiated gut cells, namely EEs and absorptive ECs, affected longevity. Upregulation of FKH in differentiated cells resulted in a significant increase in lifespan (Figure 7E; median survival +15%, $p = 2.03 \times 10^{-7}$). This finding further supports the transcriptomic and molecular analysis, and it pinpoints the pro-longevity effect of FKH to EEs and ECs.

irs1 Knockout Upregulates *CG1208*, *pmp70*, and *zip1* Orthologs in Mouse Small Intestine

Mouse small intestine is functionally and morphologically similar to *Drosophila* midgut, and it plays an essential role in the uptake of nutrients (Radtke and Clevers, 2005). We therefore measured expression levels of orthologous nutrient transporters in duodenum from female mice with intestinal *Irs1* knockout (*VillCre::Irs1*$^{lox/lox}$) and controls (*Irs1*$^{lox/lox}$) at 3 months of age. We quantified mRNA levels of *Glut8*, *Abcd3*, *Slc39a3*, and *Slc31a1* (mouse orthologs of *CG1208*, *pmp70*, *zip1*, and *ctr1B*, respectively), and we found a significant increase in *Abcd3, Glut8*, and *Slc39a3* expression (Figure S7G; t test, p = 0.006562, p = 0.029, and p = 0.019, respectively) upon intestinal *Irs1* knockout. Thus, consistent with our observations in the *Drosophila* gut, reduced IIS in mouse small intestine upregulates the expression of genes involved in nutrient transport.

DISCUSSION

The major role played by the IIS-and-TOR-signaling network during the control of metabolism, cell growth, and organismal aging has been under investigation for over two decades (Alic and Partridge, 2011). In *Drosophila*, dFOXO integrates signals from the insulin-PI3K-AKT branch of the IIS pathway (Jünger et al., 2003), and it has been considered to be the key TF mediating many of its transcriptional outputs and its effect on organismal lifespan (Slack et al., 2011). Here we have shown that a second FOX family member, FKH, interacts with both the AKT and TOR branches to regulate organismal lifespan.

While the functionality of the phosphorylation events remains to be determined, based on our biochemical data, we propose a model in which FKH can be phosphorylated by AKT and TOR and integrate signals from them (Figure 7F). IIS activity leads to FKH phosphorylation, potentially contributing to a decrease in nuclear FKH. Insulin-dependent subcellular localization of FoxA2 in mammalian cells has been subject to conflicting reports. While Wolfrum et al. (2003, 2004), reported complete nuclear exclusion of FoxA2, in vitro and in vivo in response to insulin, a study by Zhang et al. (2005) showed that FoxA2 remained nuclear under all metabolic states in mouse hepatocytes. We did not detect complete nuclear FKH exclusion in the fly but a decrease in strong nuclear FKH staining upon in vitro and ex vivo insulin stimulation. Rapamycin feeding was previously shown to increase nuclear FKH in larvae (Bülow et al., 2010;

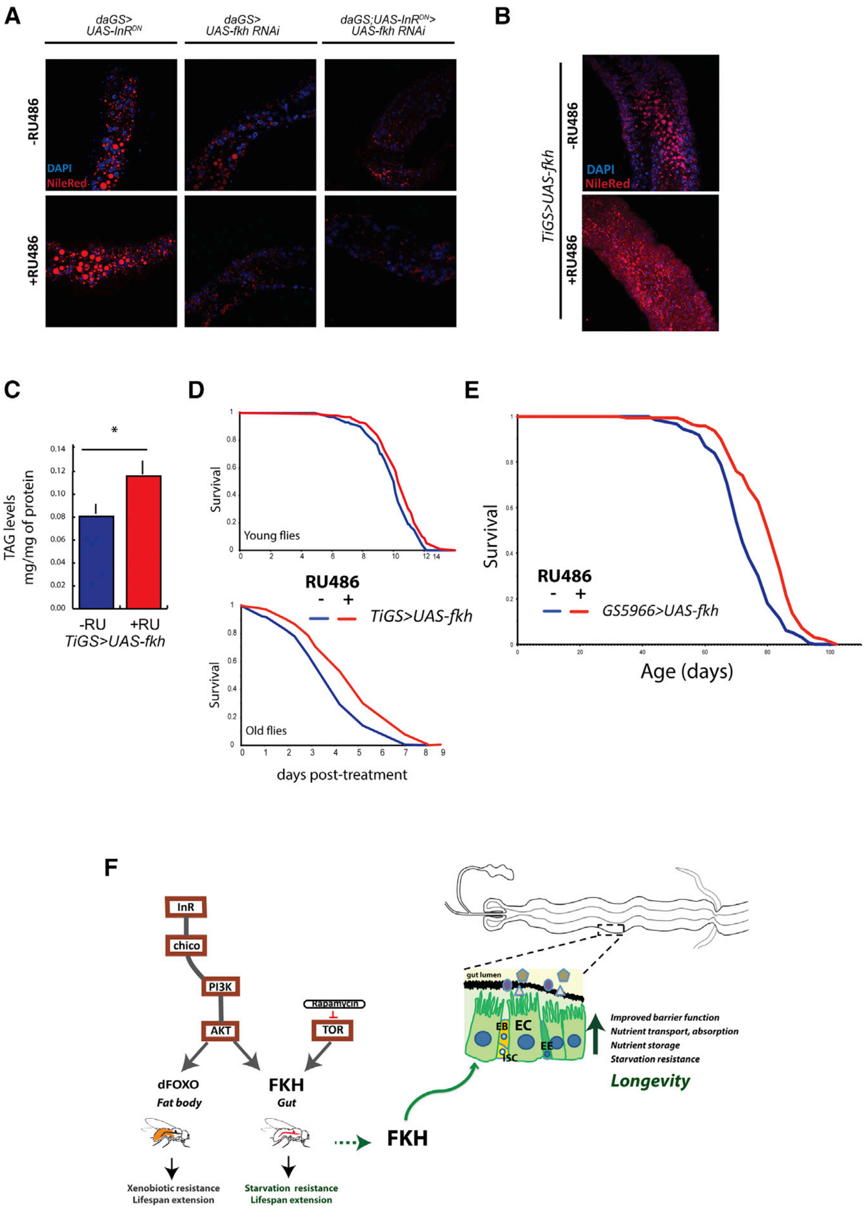

Figure 7. FKH Promotes Intestinal Nutrient Uptake upon Overexpression and in Response to Reduced IIS, and FKH Activity in Differentiated Cells of the Gut Induces Longevity

(A) Nile Red (red) and DAPI (blue) staining of the anterior region of midgut isolates from flies of genotypes *daGS > UAS-InR^DN*, *daGS;UAS-InR^DN > UAS-fkh RNAi*, and *daGS > UAS-fkh RNAi* induced or not with 200 μM RU486. See also Figure S7E.

(B) Nile Red (red) and DAPI (blue) staining of the anterior region of midgut isolates from flies of genotype *TiGS > UAS-fkh* induced or not with 200 μM RU486. See also Figure S7F.

(C) Quantification of whole-animal TAG levels normalized to total protein in flies of genotype *TiGS < UAS-fkh* induced or not with RU486 revealed significant increases upon induction (t test, $p = 0.033$; $n = 10$; error bars represent SEM).

(D) Starvation resistance of *TiGS > UAS-fkh* females was significantly increased in the presence of RU486 at 1 week ($p = 0.0047$) and 10 weeks of age ($p = 1.74 \times 10^{-5}$).

(E) Intestinal differentiated cell-specific overexpression of *UAS-fkh* under the control of *GS5966* driver extended lifespan ($p = 2.03614 \times 10^{-7}$). See also Table S5.

(F) Model. FKH integrates signals from AKT and TOR. dFOXO and FKH exert their pro-longevity effects in different tissues. In the gut, FKH improves barrier function and upregulates the expression of nutrient transporters, resulting in increased nutrient storage and resistance to starvation and overall contributing to increased longevity.

Varma et al., 2014), lending further support to a regulating role of nutrient signaling in FKH subcellular localization in the fly.

We have established an important role for FKH in *Drosophila* longevity. Ubiquitous upregulation of FKH extends lifespan, and FKH is required for reduced IIS- and rapamycin-induced longevity. We found that FKH mediates starvation resistance of IIS mutants. Interestingly, *dfoxo* plays no role in either extension of lifespan by rapamycin or in the starvation resistance of *Drosophila* IIS mutants (Slack et al., 2011). Furthermore, dFOXO is required for xenobiotic resistance of IIS mutants while FKH is not. These findings highlight different functional roles of dFOXO and FKH in determination of the phenotypes of IIS mutants and the response of lifespan to TORC1 inhibition. However, both TFs are necessary for reduced IIS-induced longevity, suggesting that they are functionally complementary. The identification of FKH as a TF downstream of both IIS and TOR branches enables a more insightful picture of the transcriptional regulation within the nutrient-signaling network, and it defies the notion that dFOXO is the key TF regulating *Drosophila* lifespan downstream of AKT kinase.

We found that the response of lifespan to DR in *Drosophila* is independent of FKH function, as has previously been shown for dFOXO (Slack et al., 2015). In contrast, the *C. elegans* FoxA homolog PHA-4 is required for DR-induced longevity, but not reduced IIS-induced lifespan extension (Panowski et al., 2007). Hence, our results show a major evolutionarily functional divergence between PHA-4 and FKH, suggesting distinct mechanisms of organismal lifespan regulation by the worm and fly FoxA orthologs.

Here, we show that intestinal FKH upregulation is sufficient to extend lifespan, whereas gut-specific dFOXO overexpression does not (Alic et al., 2014). The two TFs may, therefore, be modulating lifespan through different target genes in different tissues. We propose that tissue specificity might be the reason for the requirement of multiple TFs with lifespan-extending effects

within the nutrient-signaling network, as is the case in worms (Bishop and Guarente, 2007; Libina et al., 2003).

The *Drosophila* midgut is the functional equivalent of the mammalian stomach and small intestine, and similarly it contains distinct cell types with diverse functions (Lemaitre and Miguel-Aliaga, 2013). ISCs and EBs are progenitor cells, whereas EEs and ECs are the sole two differentiated intestinal cell types. EEs primarily play a secretory role, while ECs are responsible for immune response, absorption of nutrients, and secretion of digestive enzymes. The interaction between IIS activity in *Drosophila* gut and aging has been intensively studied (Alic et al., 2014; Biteau et al., 2010; Rera et al., 2012). Major interest has focused on ISCs, and previous work showed that reduced IIS in progenitor cells decreases age-induced proliferation of ISCs and extends lifespan (Biteau et al., 2010). However, an exclusively deterministic role for ISCs during aging is contentious (Petkau et al., 2014; Resnik-Docampo et al., 2017). In addition to intestinal homeostasis and stem cell maintenance, other aspects of gut physiology, such as immunity, secretion, and absorption, are also likely to influence organismal aging.

We found that a broader intestinal reduction of IIS activity with *TiGS* driver also results in lifespan extension but becomes detrimental upon gut-specific FKH knockdown. Despite increased longevity upon broad intestinal upregulation of FKH, restricting its overexpression to intestinal progenitor cells does not affect lifespan. Increasing FKH activity specifically in differentiated cells significantly increases longevity, suggesting this pro-longevity effect is ISC independent. Concordantly, FKH-overexpressing guts display improved barrier function without any changes in age-related ISC proliferation.

Induction of FKH activity by either lowered IIS or direct overexpression induced the expression of nutrient transporters. Aging results in decreased intestinal lipid storage and whole-body nutrient stores (Karpac et al., 2013; Rera et al., 2012), which correlates with age-related repression of lipases essential for lipid absorption (Karpac et al., 2013). Reduced intestinal storage may be due to an overall decline in absorptive capacity, resulting in decreased whole-body metabolic stores. We propose a model whereby FKH-dependent expression of nutrient transporters overcomes this decline and contributes to starvation resistance and longevity (Figure 7F). Concordantly, we show that starvation resistance declines over aging, likely due to increasingly poor nutrient uptake, but it can be enhanced by intestinal FKH induction at both young and old age. Similarly, upon reduced IIS, FKH activity may promote nutrient uptake, leading to better nutrient stores, contributing to increased starvation resistance. Recent work (Luis et al., 2016) revealed increased midgut lipid storage and expression of nutrient transporters in *Drosophila* ECs in response to DR. It is possible that upregulation of nutrient transporters is promoted by a number of lifespan-extending interventions. Intestinal barrier function is another important aspect of gut physiology improved by FKH, also likely to be contributing to its pro-longevity effects. Absorptive and barrier capacity of gut epithelial cells may well be functionally linked and also reflective of an overall improvement in the maintenance of EC physiology over age. Altogether, our data suggest that the pro-longevity effect of FKH in the gut involves ISC-independent mechanisms.

Finally, *irs1* knockout led to the upregulation of three orthologous nutrient transporters in the mouse small intestine. This suggests that an evolutionarily conserved mechanism, by which the reduction of IIS upregulates intestinal nutrient absorption, is also present in mammals. Intriguingly, mammalian FoxAs provide protection against hypoglycemia (Friedman and Kaestner, 2006). We propose that fly and mammalian FoxAs might show functional convergence in promoting nutrient uptake. Further studies are required to dissect out detailed consequences of the upregulation of nutrient transporters on digestive and absorptive capacity of the fly and mammalian gut over aging. In humans, aging causes a decrease in nutrient absorption, and malnourishment is a major problem for many elderly people (Woudstra and Thomson, 2002). Our findings may, therefore, represent new directions for therapeutic interventions to improve human health during aging.

EXPERIMENTAL PROCEDURES

Fly Husbandry

Stocks were maintained and experiments conducted at 25°C on a 12-hr light/dark cycle at 60% humidity, on food containing 10% (w/v) brewer's yeast, 5% (w/v) sucrose, and 1.5% (w/v) agar, unless otherwise noted. Please see the Supplemental Experimental Procedures for further details.

Statistical Analysis

Statistical analyses were performed in JMP (version 9) software (SAS Institute), R, or Excel (Microsoft). Survival data were analyzed with either log rank test or CPH using Excel (Microsoft) or R using the survival package (Terry Therneau, https://cran.r-project.org/web/packages/survival/index.html). qPCR analysis was performed by using either Student's t test or a linear model.

Mouse Models and Husbandry

All mice were maintained at 22°C under a 12-hr light/dark cycle (lights on from 6:00 a.m. to 6:00 p.m.). Mice were housed in groups of three to five same-sex littermates under specific pathogen-free conditions within individually ventilated cages (Techniplast UK, Kettering, Northamptonshire, UK). Mice had ad libitum access to normal chow (ssniff R/M-H phytoestrogen-poor [9% fat, 34% protein, and 57% carbohydrate], ssniff Spezialdiäten, Soest, Germany) and water. Female mice were sacrificed at 3 months. Duodenums were dissected and tissues were snap-frozen in liquid nitrogen. Details of the generation of tissue-specific *Irs1* knockout (KO) mouse are described in the Supplemental Experimental Procedures.

Ethics Statement

This study was performed in strict accordance with the recommendations and guidelines of the Federation of European Laboratory Animal Science Associations (FELASA). The protocol was approved by the Landesamt für Natur, Umwelt und Verbraucherschutz Nordrhein-Westfalen.

DATA AND SOFTWARE AVAILABILITY

The accession number for the microarray data reported in this paper is ArrayExpress: E-MTAB-6056.

SUPPLEMENTAL INFORMATION

Supplemental Information includes Supplemental Experimental Procedures, seven figures, and eight tables and can be found with this article online at https://doi.org/10.1016/j.celrep.2017.09.042.

AUTHOR CONTRIBUTIONS

E.B. and L.P. designed the experiments. E.B., M.K., J.C.R., J.A., M.C.D., and T.N. performed the experiments. E.B., N.A., and D.K.I. analyzed data. J.M.T.

and L.P. supervised the project. E.B. and L.P. wrote the manuscript. All authors approved the final submission.

ACKNOWLEDGMENTS

We thank Dr. M. Junger for providing valuable reagents and insightful information at the initial steps of the project. This work was funded by a Wellcome Trust Strategic Award (098565), Alzheimer's Research UK (ART-PG2009-4), and the Max Planck Society.

Received: April 7, 2017
Revised: July 24, 2017
Accepted: September 12, 2017
Published: October 17, 2017

REFERENCES

Alic, N., and Partridge, L. (2011). Death and dessert: nutrient signalling pathways and ageing. Curr. Opin. Cell Biol. *23*, 738–743.

Alic, N., Andrews, T.D., Giannakou, M.E., Papatheodorou, I., Slack, C., Hoddinott, M.P., Cochemé, H.M., Schuster, E.F., Thornton, J.M., and Partridge, L. (2011). Genome-wide dFOXO targets and topology of the transcriptomic response to stress and insulin signalling. Mol. Syst. Biol. *7*, 502.

Alic, N., Giannakou, M.E., Papatheodorou, I., Hoddinott, M.P., Andrews, T.D., Bolukbasi, E., and Partridge, L. (2014). Interplay of dFOXO and two ETS-family transcription factors determines lifespan in Drosophila melanogaster. PLoS Genet. *10*, e1004619.

Basset, A., Khush, R.S., Braun, A., Gardan, L., Boccard, F., Hoffmann, J.A., and Lemaitre, B. (2000). The phytopathogenic bacteria Erwinia carotovora infects Drosophila and activates an immune response. Proc. Natl. Acad. Sci. USA *97*, 3376–3381.

Binks, T., Lye, J.C., Camakaris, J., and Burke, R. (2010). Tissue-specific interplay between copper uptake and efflux in Drosophila. J. Biol. Inorg. Chem. *15*, 621–628.

Bishop, N.A., and Guarente, L. (2007). Two neurons mediate diet-restriction-induced longevity in C. elegans. Nature *447*, 545–549.

Biteau, B., Hochmuth, C.E., and Jasper, H. (2008). JNK activity in somatic stem cells causes loss of tissue homeostasis in the aging Drosophila gut. Cell Stem Cell *3*, 442–455.

Biteau, B., Karpac, J., Supoyo, S., Degennaro, M., Lehmann, R., and Jasper, H. (2010). Lifespan extension by preserving proliferative homeostasis in Drosophila. PLoS Genet. *6*, e1001159.

Bjedov, I., Toivonen, J.M., Kerr, F., Slack, C., Jacobson, J., Foley, A., and Partridge, L. (2010). Mechanisms of life span extension by rapamycin in the fruit fly Drosophila melanogaster. Cell Metab. *11*, 35–46.

Bochkis, I.M., Shin, S., and Kaestner, K.H. (2013). Bile acid-induced inflammatory signaling in mice lacking Foxa2 in the liver leads to activation of mTOR and age-onset obesity. Mol. Metab. *2*, 447–456.

Buchon, N., Osman, D., David, F.P., Fang, H.Y., Boquete, J.P., Deplancke, B., and Lemaitre, B. (2013). Morphological and molecular characterization of adult midgut compartmentalization in Drosophila. Cell Rep. *3*, 1725–1738.

Bülow, M.H., Aebersold, R., Pankratz, M.J., and Jünger, M.A. (2010). The Drosophila FoxA ortholog Fork head regulates growth and gene expression downstream of Target of rapamycin. PLoS ONE *5*, e15171.

Choi, N.H., Kim, J.G., Yang, D.J., Kim, Y.S., and Yoo, M.A. (2008). Age-related changes in Drosophila midgut are associated with PVF2, a PDGF/VEGF-like growth factor. Aging Cell *7*, 318–334.

Clancy, D.J., Gems, D., Harshman, L.G., Oldham, S., Stocker, H., Hafen, E., Leevers, S.J., and Partridge, L. (2001). Extension of life-span by loss of CHICO, a Drosophila insulin receptor substrate protein. Science *292*, 104–106.

Cornu, M., Albert, V., and Hall, M.N. (2013). mTOR in aging, metabolism, and cancer. Curr. Opin. Genet. Dev. *23*, 53–62.

de Cabo, R., Carmona-Gutierrez, D., Bernier, M., Hall, M.N., and Madeo, F. (2014). The search for antiaging interventions: from elixirs to fasting regimens. Cell *157*, 1515–1526.

Fan, X., Liang, Q., Lian, T., Wu, Q., Gaur, U., Li, D., Yang, D., Mao, X., Jin, Z., Li, Y., and Yang, M. (2015). Rapamycin preserves gut homeostasis during Drosophila aging. Oncotarget *6*, 35274–35283.

Friedman, J.R., and Kaestner, K.H. (2006). The Foxa family of transcription factors in development and metabolism. Cell. Mol. Life Sci. *63*, 2317–2328.

Giannakou, M.E., Goss, M., Jünger, M.A., Hafen, E., Leevers, S.J., and Partridge, L. (2004). Long-lived Drosophila with overexpressed dFOXO in adult fat body. Science *305*, 361.

Grandison, R.C., Piper, M.D., and Partridge, L. (2009). Amino-acid imbalance explains extension of lifespan by dietary restriction in Drosophila. Nature *462*, 1061–1064.

Harrison, D.E., Strong, R., Sharp, Z.D., Nelson, J.F., Astle, C.M., Flurkey, K., Nadon, N.L., Wilkinson, J.E., Frenkel, K., Carter, C.S., et al. (2009). Rapamycin fed late in life extends lifespan in genetically heterogeneous mice. Nature *460*, 392–395.

Heintzman, N.D., Hon, G.C., Hawkins, R.D., Kheradpour, P., Stark, A., Harp, L.F., Ye, Z., Lee, L.K., Stuart, R.K., Ching, C.W., et al. (2009). Histone modifications at human enhancers reflect global cell-type-specific gene expression. Nature *459*, 108–112.

Hsu, A.L., Murphy, C.T., and Kenyon, C. (2003). Regulation of aging and age-related disease by DAF-16 and heat-shock factor. Science *300*, 1142–1145.

Hwangbo, D.S., Gershman, B., Tu, M.P., Palmer, M., and Tatar, M. (2004). Drosophila dFOXO controls lifespan and regulates insulin signalling in brain and fat body. Nature *429*, 562–566.

Jünger, M.A., Rintelen, F., Stocker, H., Wasserman, J.D., Végh, M., Radimerski, T., Greenberg, M.E., and Hafen, E. (2003). The Drosophila forkhead transcription factor FOXO mediates the reduction in cell number associated with reduced insulin signaling. J. Biol. *2*, 20.

Kapahi, P., Zid, B.M., Harper, T., Koslover, D., Sapin, V., and Benzer, S. (2004). Regulation of lifespan in Drosophila by modulation of genes in the TOR signaling pathway. Curr. Biol. *14*, 885–890.

Karpac, J., Biteau, B., and Jasper, H. (2013). Misregulation of an adaptive metabolic response contributes to the age-related disruption of lipid homeostasis in Drosophila. Cell Rep. *4*, 1250–1261.

Kenyon, C., Chang, J., Gensch, E., Rudner, A., and Tabtiang, R. (1993). A C. elegans mutant that lives twice as long as wild type. Nature *366*, 461–464.

Kinoshita, E., Kinoshita-Kikuta, E., Takiyama, K., and Koike, T. (2006). Phosphate-binding tag, a new tool to visualize phosphorylated proteins. Mol. Cell. Proteomics *5*, 749–757.

Lam, E.W., Brosens, J.J., Gomes, A.R., and Koo, C.Y. (2013). Forkhead box proteins: tuning forks for transcriptional harmony. Nat. Rev. Cancer *13*, 482–495.

Lemaitre, B., and Miguel-Aliaga, I. (2013). The digestive tract of Drosophila melanogaster. Annu. Rev. Genet. *47*, 377–404.

Li, Y., Wang, W.J., Cao, H., Lu, J., Wu, C., Hu, F.Y., Guo, J., Zhao, L., Yang, F., Zhang, Y.X., et al. (2009). Genetic association of FOXO1A and FOXO3A with longevity trait in Han Chinese populations. Hum. Mol. Genet. *18*, 4897–4904.

Libina, N., Berman, J.R., and Kenyon, C. (2003). Tissue-specific activities of C. elegans DAF-16 in the regulation of lifespan. Cell *115*, 489–502.

Luis, N.M., Wang, L., Ortega, M., Deng, H., Katewa, S.D., Li, P.W., Karpac, J., Jasper, H., and Kapahi, P. (2016). Intestinal IRE1 Is Required for Increased Triglyceride Metabolism and Longer Lifespan under Dietary Restriction. Cell Rep. *17*, 1207–1216.

Murphy, C.T., McCarroll, S.A., Bargmann, C.I., Fraser, A., Kamath, R.S., Ahringer, J., Li, H., and Kenyon, C. (2003). Genes that act downstream of DAF-16 to influence the lifespan of Caenorhabditis elegans. Nature *424*, 277–283.

Panowski, S.H., Wolff, S., Aguilaniu, H., Durieux, J., and Dillin, A. (2007). PHA-4/Foxa mediates diet-restriction-induced longevity of C. elegans. Nature *447*, 550–555.

Passtoors, W.M., Beekman, M., Deelen, J., van der Breggen, R., Maier, A.B., Guigas, B., Derhovanessian, E., van Heemst, D., de Craen, A.J., Gunn, D.A., et al. (2013). Gene expression analysis of mTOR pathway: association with human longevity. Aging Cell *12*, 24–31.

Pawlikowska, L., Hu, D., Huntsman, S., Sung, A., Chu, C., Chen, J., Joyner, A.H., Schork, N.J., Hsueh, W.C., Reiner, A.P., et al.; Study of Osteoporotic Fractures (2009). Association of common genetic variation in the insulin/IGF1 signaling pathway with human longevity. Aging Cell *8*, 460–472.

Petkau, K., Parsons, B.D., Duggal, A., and Foley, E. (2014). A deregulated intestinal cell cycle program disrupts tissue homeostasis without affecting longevity in Drosophila. J. Biol. Chem. *289*, 28719–28729.

Powers, R.W., 3rd, Kaeberlein, M., Caldwell, S.D., Kennedy, B.K., and Fields, S. (2006). Extension of chronological life span in yeast by decreased TOR pathway signaling. Genes Dev. *20*, 174–184.

Puig, O., Marr, M.T., Ruhf, M.L., and Tjian, R. (2003). Control of cell number by Drosophila FOXO: downstream and feedback regulation of the insulin receptor pathway. Genes Dev. *17*, 2006–2020.

Qin, Q., Wang, X., and Zhou, B. (2013). Functional studies of Drosophila zinc transporters reveal the mechanism for dietary zinc absorption and regulation. BMC Biol. *11*, 101.

Radtke, F., and Clevers, H. (2005). Self-renewal and cancer of the gut: two sides of a coin. Science *307*, 1904–1909.

Regan, J.C., Khericha, M., Dobson, A.J., Bolukbasi, E., Rattanavirotkul, N., and Partridge, L. (2016). Sex difference in pathology of the ageing gut mediates the greater response of female lifespan to dietary restriction. eLife *5*, e10956.

Rera, M., Clark, R.I., and Walker, D.W. (2012). Intestinal barrier dysfunction links metabolic and inflammatory markers of aging to death in Drosophila. Proc. Natl. Acad. Sci. USA *109*, 21528–21533.

Resnik-Docampo, M., Koehler, C.L., Clark, R.I., Schinaman, J.M., Sauer, V., Wong, D.M., Lewis, S., D'Alterio, C., Walker, D.W., and Jones, D.L. (2017). Tricellular junctions regulate intestinal stem cell behaviour to maintain homeostasis. Nat. Cell Biol. *19*, 52–59.

Robida-Stubbs, S., Glover-Cutter, K., Lamming, D.W., Mizunuma, M., Narasimhan, S.D., Neumann-Haefelin, E., Sabatini, D.M., and Blackwell, T.K. (2012). TOR signaling and rapamycin influence longevity by regulating SKN-1/Nrf and DAF-16/FoxO. Cell Metab. *15*, 713–724.

Selman, C., Lingard, S., Choudhury, A.I., Batterham, R.L., Claret, M., Clements, M., Ramadani, F., Okkenhaug, K., Schuster, E., Blanc, E., et al. (2008). Evidence for lifespan extension and delayed age-related biomarkers in insulin receptor substrate 1 null mice. FASEB J. *22*, 807–818.

Slack, C., Giannakou, M.E., Foley, A., Goss, M., and Partridge, L. (2011). dFOXO-independent effects of reduced insulin-like signaling in Drosophila. Aging Cell *10*, 735–748.

Slack, C., Alic, N., Foley, A., Cabecinha, M., Hoddinott, M.P., and Partridge, L. (2015). The Ras-Erk-ETS-Signaling Pathway Is a Drug Target for Longevity. Cell *162*, 72–83.

Squarize, C.H., Castilho, R.M., Bugge, T.H., and Gutkind, J.S. (2010). Accelerated wound healing by mTOR activation in genetically defined mouse models. PLoS ONE *5*, e10643.

Suh, Y., Atzmon, G., Cho, M.O., Hwang, D., Liu, B., Leahy, D.J., Barzilai, N., and Cohen, P. (2008). Functionally significant insulin-like growth factor I receptor mutations in centenarians. Proc. Natl. Acad. Sci. USA *105*, 3438–3442.

Tatar, M., Kopelman, A., Epstein, D., Tu, M.P., Yin, C.M., and Garofalo, R.S. (2001). A mutant Drosophila insulin receptor homolog that extends life-span and impairs neuroendocrine function. Science *292*, 107–110.

Teleman, A.A., Hietakangas, V., Sayadian, A.C., and Cohen, S.M. (2008). Nutritional control of protein biosynthetic capacity by insulin via Myc in Drosophila. Cell Metab. *7*, 21–32.

Tullet, J.M., Hertweck, M., An, J.H., Baker, J., Hwang, J.Y., Liu, S., Oliveira, R.P., Baumeister, R., and Blackwell, T.K. (2008). Direct inhibition of the longevity-promoting factor SKN-1 by insulin-like signaling in C. elegans. Cell *132*, 1025–1038.

Varma, D., Bülow, M.H., Pesch, Y.Y., Loch, G., and Hoch, M. (2014). Forkhead, a new cross regulator of metabolism and innate immunity downstream of TOR in Drosophila. J. Insect Physiol. *69*, 80–88.

Wang, L., Lawrence, J.C., Jr., Sturgill, T.W., and Harris, T.E. (2009). Mammalian target of rapamycin complex 1 (mTORC1) activity is associated with phosphorylation of raptor by mTOR. J. Biol. Chem. *284*, 14693–14697.

Weigel, D., Jürgens, G., Küttner, F., Seifert, E., and Jäckle, H. (1989). The homeotic gene fork head encodes a nuclear protein and is expressed in the terminal regions of the Drosophila embryo. Cell *57*, 645–658.

Willcox, B.J., Donlon, T.A., He, Q., Chen, R., Grove, J.S., Yano, K., Masaki, K.H., Willcox, D.C., Rodriguez, B., and Curb, J.D. (2008). FOXO3A genotype is strongly associated with human longevity. Proc. Natl. Acad. Sci. USA *105*, 13987–13992.

Wolfrum, C., Besser, D., Luca, E., and Stoffel, M. (2003). Insulin regulates the activity of forkhead transcription factor Hnf-3beta/Foxa-2 by Akt-mediated phosphorylation and nuclear/cytosolic localization. Proc. Natl. Acad. Sci. USA *100*, 11624–11629.

Wolfrum, C., Asilmaz, E., Luca, E., Friedman, J.M., and Stoffel, M. (2004). Foxa2 regulates lipid metabolism and ketogenesis in the liver during fasting and in diabetes. Nature *432*, 1027–1032.

Woudstra, T., and Thomson, A.B. (2002). Nutrient absorption and intestinal adaptation with ageing. Best Pract. Res. Clin. Gastroenterol. *16*, 1–15.

Yamaguchi, T., Kakefuda, R., Tajima, N., Sowa, Y., and Sakai, T. (2011). Antitumor activities of JTP-74057 (GSK1120212), a novel MEK1/2 inhibitor, on colorectal cancer cell lines in vitro and in vivo. Int. J. Oncol. *39*, 23–31.

Zerofsky, M., Harel, E., Silverman, N., and Tatar, M. (2005). Aging of the innate immune response in Drosophila melanogaster. Aging Cell *4*, 103–108.

Zhang, L., Rubins, N.E., Ahima, R.S., Greenbaum, L.E., and Kaestner, K.H. (2005). Foxa2 integrates the transcriptional response of the hepatocyte to fasting. Cell Metab. *2*, 141–148.

Cell Reports, Volume *21*

Supplemental Information

Intestinal Fork Head Regulates Nutrient Absorption and Promotes Longevity

Ekin Bolukbasi, Mobina Khericha, Jennifer C. Regan, Dobril K. Ivanov, Jennifer Adcott, Miranda C. Dyson, Tobias Nespital, Janet M. Thornton, Nazif Alic, and Linda Partridge

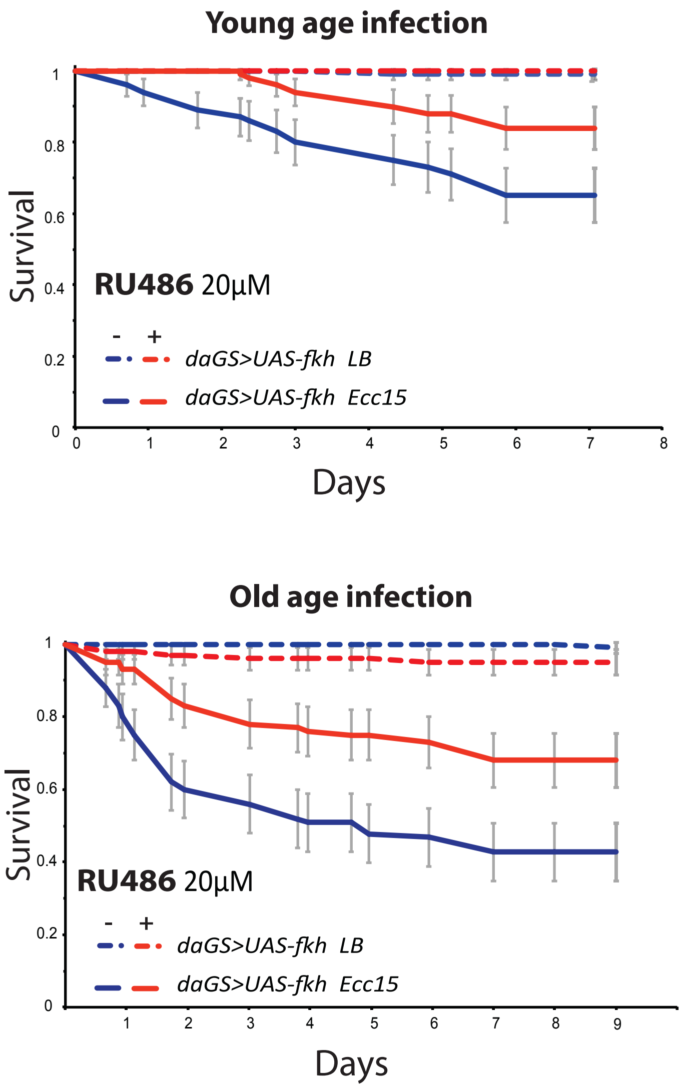

Figure S1: Over-expression of *fkh* improves immune response to *Ecc15*. Related to Figure 1. Survival curves of flies ubiquitously overexpressing *UAS-fkh* transgene under the control of *daGS* driver following systemic *Ecc15* infection or sham infection with LB only at 1 week of age (upper panel) or 7 weeks of age (lower panel). No significant difference in survival was observed at 20 µM RU486 in Sham infected flies at young (p= 0.317) and old age (p=0.093). Significant increase in survival following *Ecc15* infection was observed at 20 µM RU486 at young (p= 0.00014) and old age (p=0.0001).

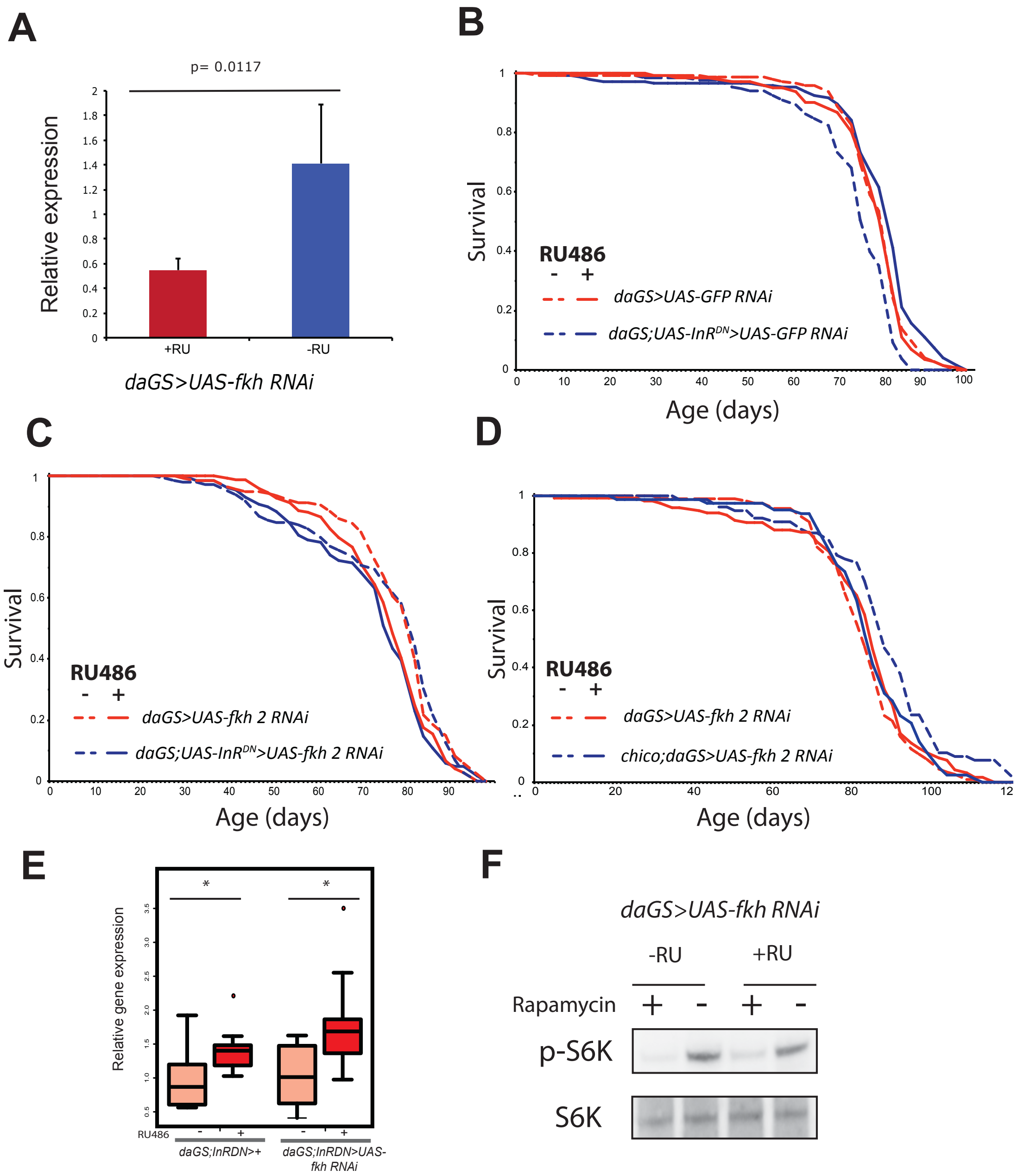

Figure S2: Over-expression of *fkh RNAi 2* transgene abolishes lifespan extension observed upon reduced IIS activity and IIS and rapamycin still exert downstream biochemical effects upon *fkh* RNAi. Related to Figure 3. (A) *fkh* mRNA was quantified relative to *actin5C* by qPCR in whole flies of genotype *daGS>UAS-fkh RNAi* induced or not with 200 µM RU486. Induction of *fkh* RNAi resulted in a significant decrease in *fkh* expression levels (Student's t-test, $p < 0.05$, n=5, error bars represent SD). (B) *daGS;UAS-InR^{DN}>UAS-GFP RNAi* females show increased lifespan in the presence of RU486 ($p = 7.9 \times 10^{-11}$). Survival of *daGS >UAS-GFP RNAi* females was not different between –RU486 and +RU468 conditions (p= 0.78).). See also Table S6A (C) *daGS>UAS-fkh RNAi 2* and *daGS;UAS-InR^{DN}>UAS-fkh RNAi 2* flies showed significantly decreased lifespan in the presence of RU486 (p= 0.0075 and p= 0.0019 respectively). Reduction of IIS via simultaneous over-expression of *UAS-InR^{DN}* and *fkh RNAi 2* did not result in lifespan extension since no significant difference in survival was detected between *daGS>UAS-fkh RNAi 2* +RU486 and *daGS;UAS-InR^{DN}>UAS-fkh RNAi 2* +RU486 flies (p= 0.62).). See also Table S6B. (D) *chico* mutation extended lifespan in *daGS>UAS-fkh RNAi 2* flies in the absence of RU486 (p= 0.00031), but not in its presence (p=0.802). See also Table S6C. (E) *4EBP* mRNA was quantified relative to *tubulin* by qPCR in whole flies induced or not with 200 µM RU486. Reduction of IIS resulted in a significant increase in *4EBP* levels in both wild type and fkh RNAi background (Student's t-test, $p < 0.05$, n=9-10). (F) Immunoblotting of whole fly extracts using phospho-specific and total dS6K antibodies reveals decreased pS6K levels upon rapamycin treatment in flies induced or not with 200 µM RU486

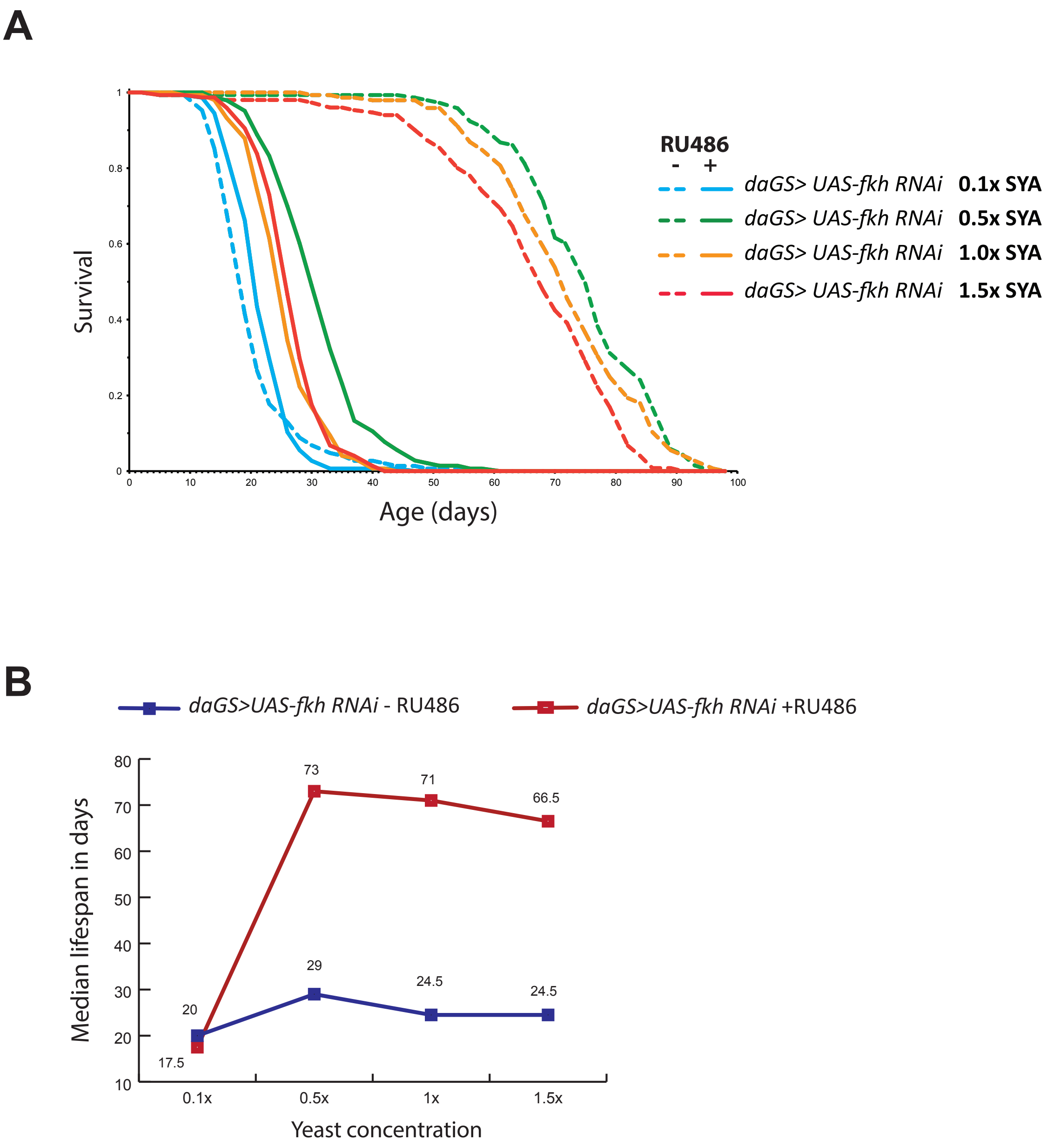

Figure S3: FKH does not mediate the response of lifespan to DR. Related to Figure 3. (A) Survival curves and (B) median lifespan values of *daGS> UAS-fkh RNAi* female flies in the presence or absence of RU486 and across different yeast concentrations (0.1x, 0.5x, 1.0x, and 1.5x yeast) on SYA food. Log-rank test revealed a significant difference in survival of *daGS>UAS-fkh RNAi* +RU486 flies at 0.5x yeast SYA food compared to *daGS>UAS-fkh RNAi* +RU486 flies at 1.0x and 1.5x yeast SYA food concentration ($p = 1.36 \times 10^{-10}$ and $p = 1.58 \times 10^{-9}$, respectively). For – RU486 conditions, at 0.1xyeast n= 147 deaths /9 censors , at 0.5xyeast n= 141 deaths /10 censors, at 1.0xyeast n= 145 deaths /3 censors, at 1.5xyeast n= 148 deaths /7 censors.). For +RU486 conditions, at 0.1xyeast n= 145 deaths /4 censors , at 0.5xyeast n= 143 deaths /8 censors, at 1.0xyeast n= 148 deaths /5 censors, at 1.5xyeast n= 148 deaths /4 censors.

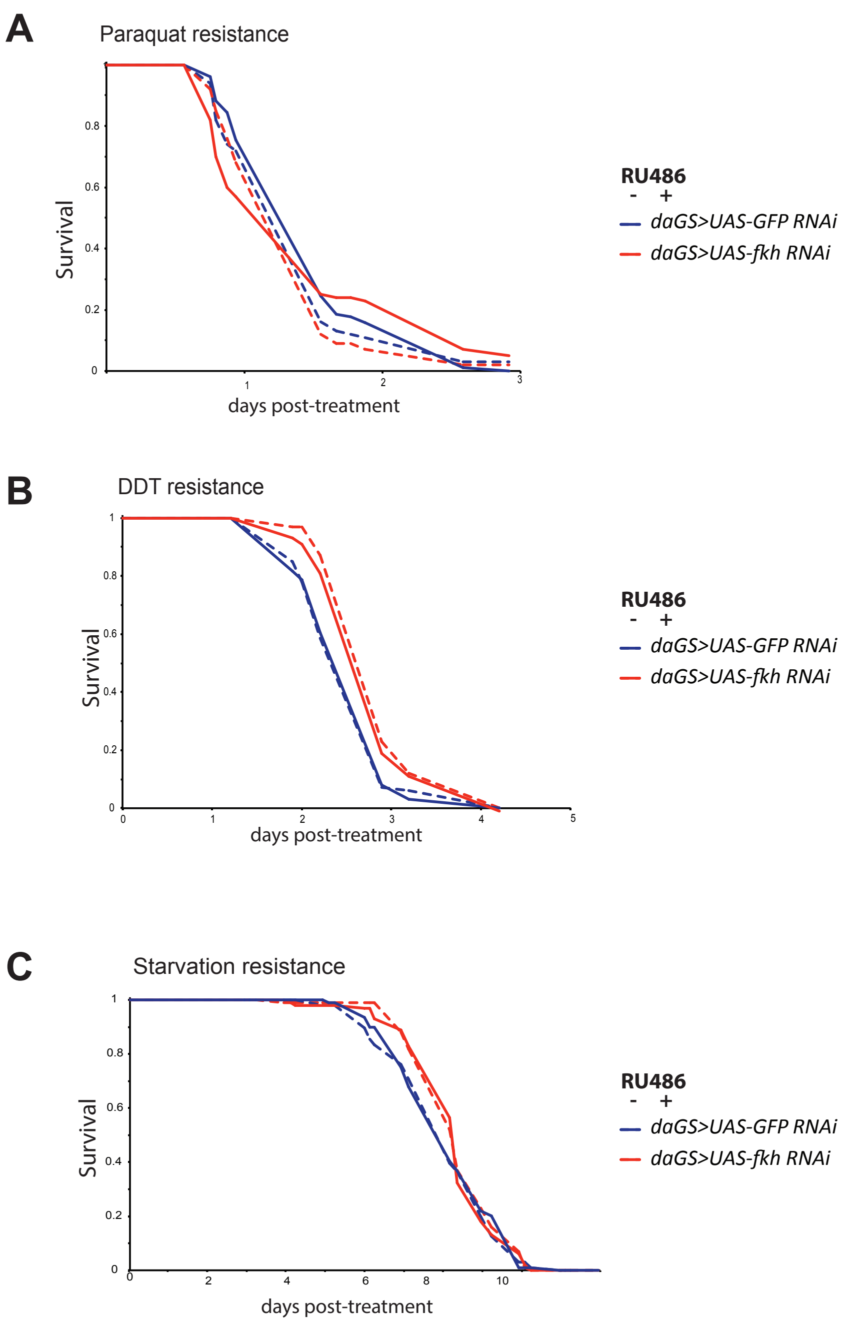

Figure S4: Over-expression of *GFP RNAi* or *fkh RNAi* alone does not affect stress resistance. Related to Figure 4. (A-C) Survival curves in response to paraquat treatment, DDT treatment and amino acid starvation of female flies induced to ubiquitously express either *UAS-GFP-RNAi* or *UAS-fkh-RNAi* transgenes (*daGS >UAS-GFP RNAi* or *daGS >UAS-fkh RNAi)* by using RU486 compared with uninduced controls. No significant difference in lifespan was observed between + and - RU486 conditions for *daGS >GFP RNAi* and for *daGS >fkh RNAi* upon (A) paraquat treatment (p= 0.072 and p=0.73 respectively), (B) DDT feeding (p= 0.93 and p=0.44 respectively) and (C) amino acid starvation (p= 0.78 and p=0.48 respectively). See also Table S7.

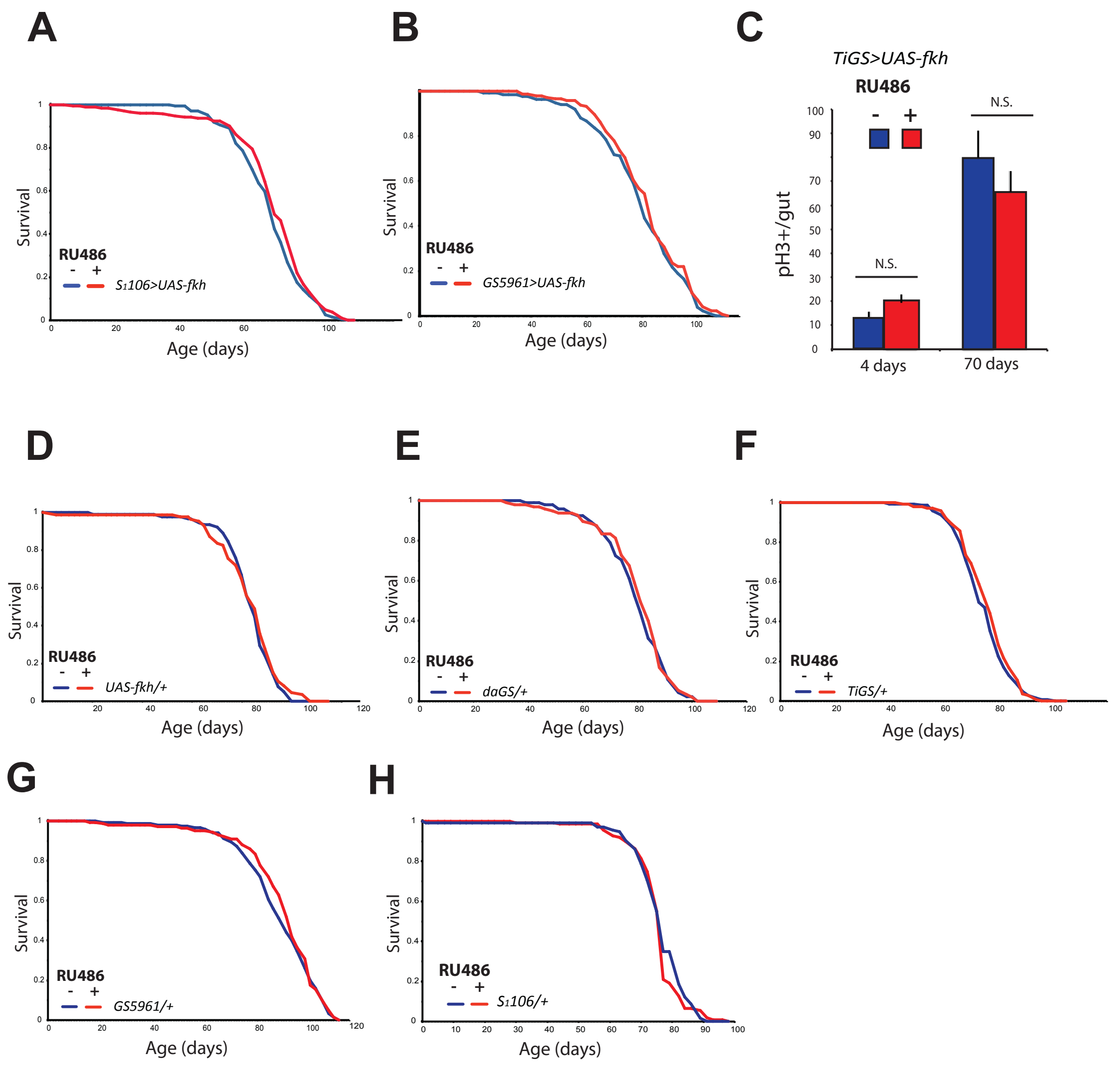

Figure S5: Overexpression of FKH in the progenitor cells does not affect lifespan and intestinal FKH up-regulation does not affect ISC proliferation. Related to Figure 5. (A) Adult abdominal fat body and gut specific over-expression of *fkh* transgene under the control of S_1106 driver did not affect lifespan (p= 0.093). (B) ISC and EB specific over-expression of the *fkh* transgene under the control of *GS5961* driver did not affect lifespan (p= 0.21). (C) Quantification of pH3+ cells per gut at 4 and 70 days of age in flies of genotype *TiGS>UAS-fkh* showed no difference in mitoses between – and + RU486 conditions in young and old flies (Student's t-test, p= 0.42 and p=0.51, n= 19 to 22 guts per condition, error bars represent SEM). See Also Table S8 A-B. (D) Presence of RU486 in the food did not affect survival of flies with *UAS-fkh* transgene alone (p= 0.48), (E) *daGS* driver alone (p=0.67), (F) TiGS driver alone (p=0.294), (G) *GS5961* driver alone (p= 0.38) and (H) $S1_{106}$ driver alone (p=0.42). See also Table S8 C-G

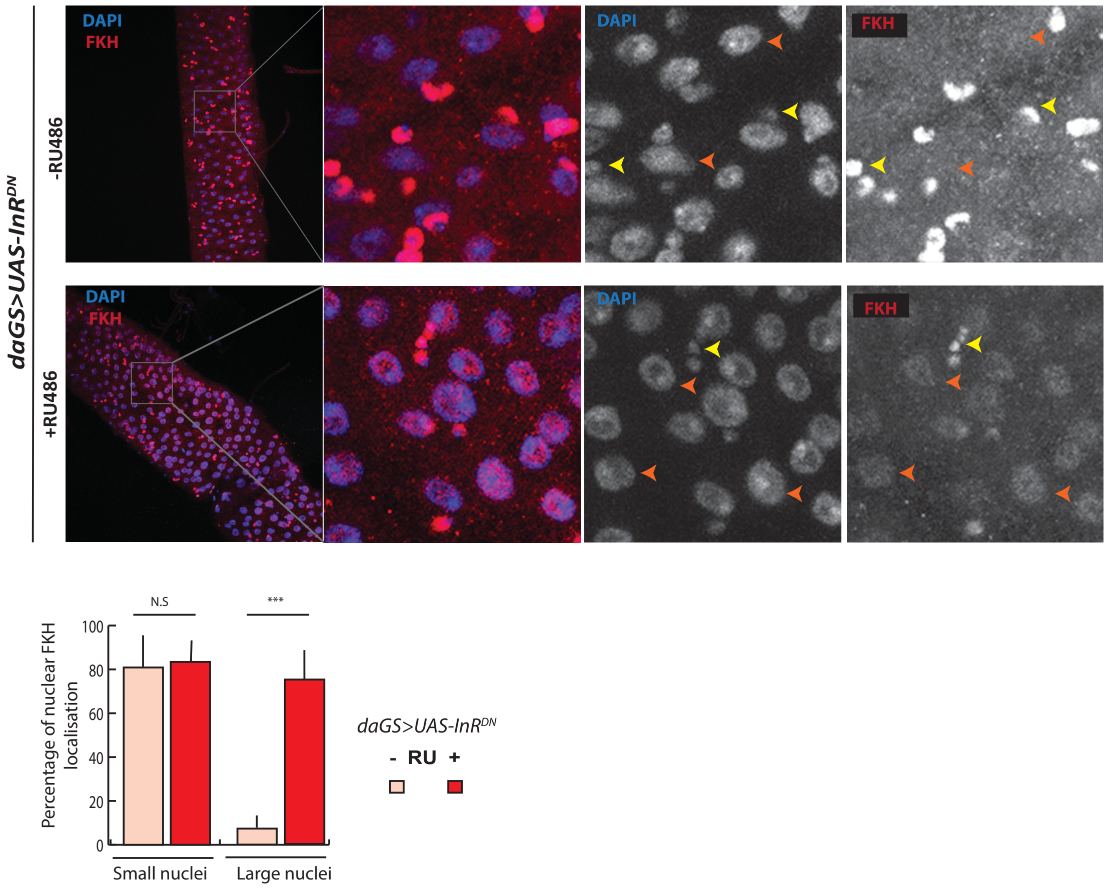

Figure S6: Reduced IIS leads nuclear localization of FKH in enterocytes. Related to Figure 6. *Drosophila* adult guts were dissected from flies of genotype *daGS>UAS-InR^{DN}* induced or not with 200 µM RU486. Endogenous FKH (red) and DAPI-stained nuclei (blue) were visualized by immunofluorescence in the mid-gut. Nuclear FKH localisation was quantified in two cell subtypes characterized by small and larger nuclei. Small nuclei and large nuclei are indicated by yellow and orange arrowheads respectively. Percentage of nuclear FKH staining was dramatically increased specifically in cells with large nuclei in the +RU486 condition (Student's t-test, $p< 0.0001$, $n=5$) whereas it remained unchanged in cells with smaller nuclei (Student's t-test, $p=0.78$, $n=5$, error bars represent SD).

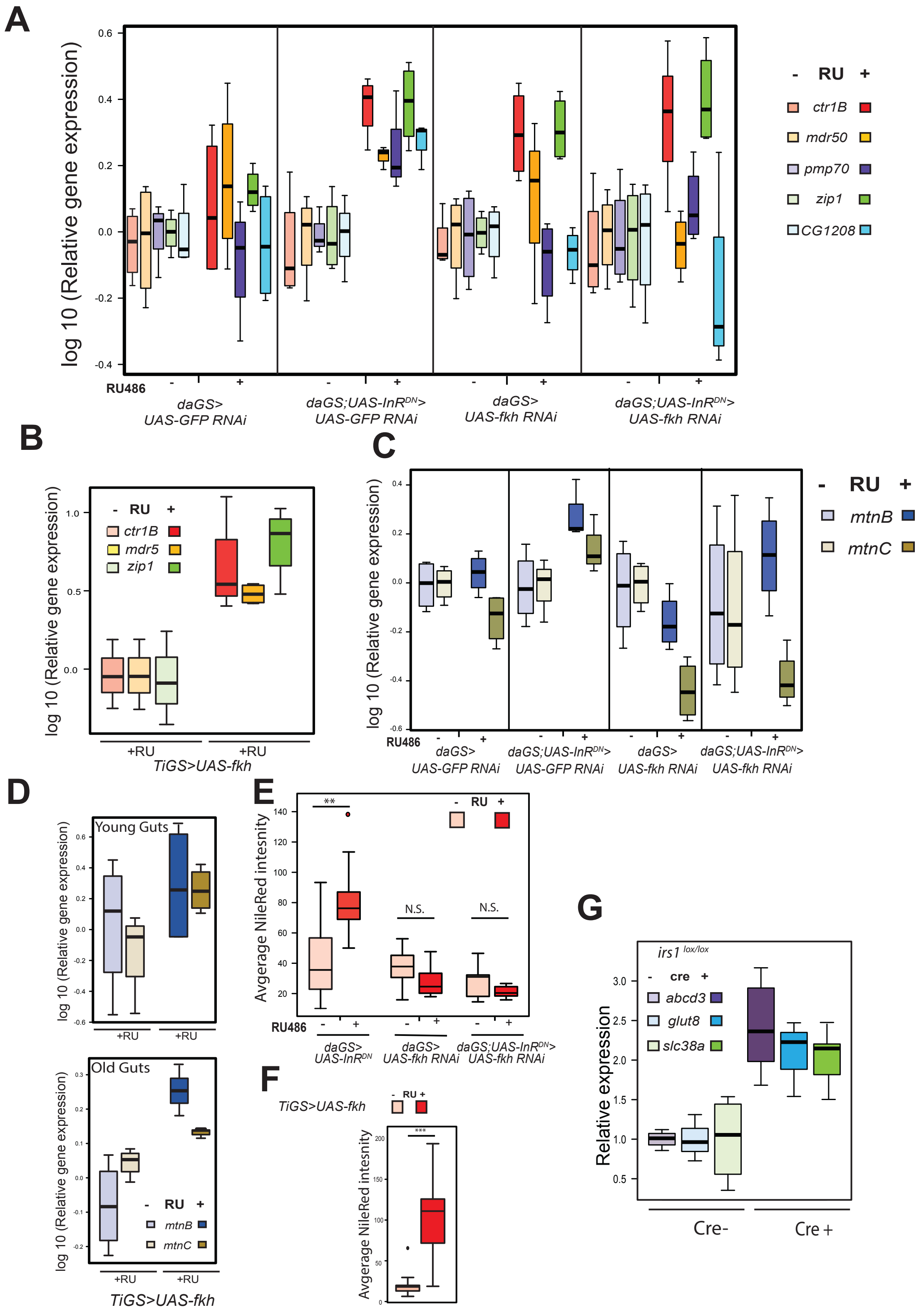

Figure S7: Expression of nutrient transporters and nutrient uptake is increased in the gut upon reduced IIS and FKH overexpression. Related to Figures 6 and 7. (A) *ctr1B*, *mdr50*, *zip1*, *pmp70* and *CG1208 mRNAs* were quantified relative to *actin5C* by qPCR in guts of *daGS >UAS-GFP RNAi, daGS;UAS-InR^{DN}>UAS-GFP RNAi, daGS >UAS-fkh RNAi and daGS;UAS-InR^{DN}>UAS-fkh RNAi* flies induced or not with RU486. Boxplots show log- 10 derived relative expression with – RU486 values set to zero. Data were analyzed with a linear model and the effects of the interaction between RU486 and genotype was significant (p=0.0004, n=3-4), whereas 3-way interaction between RU486, genotype and different gene categories was not significant (p= 0.2923). *daGS;UAS-InRDN>UAS-GFP RNAi* +RU condition showed significantly increased expression levels and was different form all other

conditions (t-test, $p<0.05$, n=3-4). (B) *ctr1B, mdr50 and zip1* mRNA levels quantified relative to *actin5C* by qRT-PCR in adult guts isolated from flies of genotype *TiGS>UAS-fkh* induced or not with 200 µM RU486. Boxplots show log-10 derived relative expression with - RU486 values set to zero. Induction of *fkh* resulted in a significant increase in *ctr1B, mdr50* and *zip1* expression levels (Student's t-test, p= 0.02, p= 0.006 and p= 0.007 respectively, n=4). (C) *mtnB* and *mtn*C mRNAs were quantified relative to *actin5C* by qPCR in guts of *daGS >UAS-GFP RNAi, daGS;UAS-InR^{DN}>UAS-GFP RNAi, daGS >UAS-fkh RNAi and daGS;UAS-InR^{DN}>UAS-fkh RNAi* flies induced or not with 200 µM RU486. Boxplots show log- 10 derived relative expression with – RU486 values set to zero. Data were analyzed with a linear model. The effect of interaction between RU and genotype was significant (p=0.0015, n=3-4) and the 3-way interaction between RU, genotype and different gene categories was not significant (p=0.5065, n=3-4). *daGS;UAS-InRDN>UAS-GFP RNAi* +RU condition was different from all others (t-test, $p<0.05$, n=3-4). (D) *mtnB* and *mtnC* mRNA levels quantified relative to *actin5C* by qPCR in young and old adult guts isolated from flies of genotype *TiGS>UAS-fkh* induced or not with 200 µM RU486. Boxplots show log-10 derived relative expression. Induction of *fkh* resulted in a significant increase in *mtnC* expression levels in young and old guts (t-test, p= 0.02 and p=0.005 respectively, n=4) and in a significant increase in *mtnB* expression levels in old guts (t-test, p= 0.01, n=4). (E) Quantification of the NileRed staining of the anterior region of mid-guts isolates from flies of genotypes *daGS>UAS-InR^{DN}*, *daGS;UAS-InR^{DN} > UAS-fkh RNAi* and *daGS > UAS-fkh RNAi* induced or not with 200 µM RU486. Boxplots show quantification of NileRed intensity in arbitrary units. NileRed staining is significantly increased in +RU486 condition of genotype *daGS>UAS-InR^{DN}* (t-test, p=0.0019, n=9-11). No significance was detected between + and – RU486 conditions of genotypes *daGS;UAS-InR^{DN} > UAS-fkh RNAi* and *daGS > UAS-fkh RNAi* (t-test, p=0.094 and p=0.059, respectively, n=9-11). (F) Quantification of the NileRed staining of the anterior region of mid-guts isolates from flies of genotype *TiGS>UAS-fkh* induced or not with 200 µM RU486. Boxplots show quantification of NileRed intensity in arbitrary units. NileRed staining is significantly increased in RU486 condition part of the mid-gut (t-test, p= 2.26×10^{-8}, n=15-19). (G) *Abcd3, Glut8 and Slc39a3* mRNA levels quantified relative to *actin* by qPCR in duodenum of 3 month old female mice of genotype *Irs1$^{lox/lox}$* (control) and *VillCre::Irs1$^{lox/lox}$* (*Irs1* knock-out). Boxplots show relative expression with control values set to 1. *Irs1* knock-out resulted in a significant increase in *Abcd3, Glut8* and *Slc39a* expression levels (Student's t-test, p= 0.006562, p=0.029 and p= 0.019 respectively, n=3-4).

Table S1- Ubiquitous FKH over expression extends lifespan. Related to Figure 1.

A. Survival data for da*GS>UAS-fkh* (related to Figure 1A)

Genotype/Treatment	Med Lifespan (days)	Max lifespan (days)	n Dead	n Censored	% Median Increase	% Maximum Increase	p-value (log rank) vs control
Repeat 1							
RU486-	79	95.5	93	5			
10 uM RU486	86	107	93	1	8.86	12.0418848	6.95E-06
50 uM RU486	84	102.5	98	2	6.33	7.32984293	0.017708512
100 uM RU486	81.5	101	92		3.16	5.7591623	0.181253206
200 uM RU486	52	93	105	1	-34.18	-2.617801	2.88501E-07

A. Survival data for da*GS>UAS-fkh* (related to Figure 1A)

Genotype/Treatment	Med Lifespan (days)	Max lifespan (days)	n Dead	n Censored	% Median Increase	% Maximum Increase	p-value (log rank) vs control
Repeat 1							
RU486-	78	98	90	4			
10 uM RU486	85	101	91	5	8.97	3.06122449	0.003
20 uM RU486	89.5	102	89	4	14.74359	4.08163265	6.3303E-06

Table S2- FKH is required for reduced IIS and rapamycin induced longevity. Related to Figure 3.

A. Survival data for da*GS;UAS-InRDN>GFP RNAi* +/-RU486 (related to Figure 3A)

Genotype/Treatment	Med Lifespan (days)	Max Lifespan (days)	n Dead	n Censored	% Median change (vs -RU)	% Maximum change (vs -RU)	p-value (log rank) vs control
Repeat 1							
daGS>GFP RNAiRU486-	62	78	150	0			
daGS>GFP RNAiRU486+	66.5	73.5	146	4			0.50
daGS;InRDN>GFP RNAiRU486-	64	78	140	10			
daGS;InRDN>GFP RNAiRU486+	73.5	80.5	146	4	14.84	3.205128205	4.45E-10

Genotype/Treatment	Med Lifespan (days)	Max Lifespan (days)	n Dead	n Censored	% Median change (vs -RU)	% Maximum change (vs -RU)	p-value (log rank) vs control
Repeat 2							
daGS>GFP RNAiRU486-	74.0	85.5	141	11			
daGS>GFP RNAiRU486+	74.0	85.5	148	12			0.918433719
daGS;InRDN>GFP RNAiRU486-	71.5	83.0	140	5			
daGS;InRDN>GFP RNAiRU486+	78.5	92.5	147	12	9.79	11.45	3.62E-09

B. Survival data for da*GS;UAS-InRDN>fkh RNAi* +/-RU486 (related to Figure 3B)

Genotype/Treatment	Med Lifespan (days)	Max Lifespan (days)	n Dead	n Censored	% Median change (vs -RU)	% Maximum change (vs -RU)	p-value (log rank)
Repeat 1							
daGS>fkh RNAiRU486-	76	90.5	130	5			
daGS>fkh RNAiRU486+	36	52.5	146	2	-52.63157895	-41.99	1.97E-54 (vs RU-)
daGS;InRDN>fkh RNAiRU486-	80.5	94.5	131	8			
daGS;InRDN>fkh RNAiRU486+	31.5	59.5	150	7	-60.87	-37.04	2.59E-63 (vs RU-)
					daGS>fkh RNAiRU486	vs.	**0.08**
					daGS;InRDN>fkh RNAiRU486+		

Genotype/Treatment	Med Lifespan (days)	Max Lifespan (days)	n Dead	n Censored	% Median change (vs -RU)	% Maximum change (vs -RU)	p-value (log rank)
Repeat 2							
daGS>fkh RNAiRU486-	76	92.5	154	4			
daGS>fkh RNAiRU486+	31.5	43.5	143	0	-58.55	-52.97	1.54 (vs RU-)
daGS;InRDN>fkh RNAiRU486-	76	85.5	136	9			
daGS;InRDN>fkh RNAiRU486+	32	50.5	154	8	-57.89	-40.94	3.07(vs RU-)
						daGS>fkh RNAiRU486+	**0.11**
						daGS;InRDN>fkh RNAiRU486+	

C.Survival data for daGS;chico> GFP RNAi(related to Figure 3C)

Genotype/Treatment	Med Lifespan (days)	Max Lifespan (days)	n Dead	n Censored	% Median change (vs wt)	% Maximum change (vs wt)	p-value (log rank)
Repeat 1							
daGS>GFP RNAiRU486-	83	97	142	1			
daGS>GFP RNAiRU486+	85	96	152	3			
chico;daGS>GFP RNAiRU486-	90	108.5	90	0	8.43	11.86	2.71E-06
chico;daGS>GFP RNAiRU486+	90	106	89	5	5.88	10.42	6.28E-07

D.Survival data for daGS;chico> UAS-fkh RNAi(related to Figure 3D)

Genotype/Treatment	Median Lifespan (days)	Maximum lifespan	n Dead	n Censored	% Median change (vs wt)	% Maximum change (vs wt)	p-value (log rank)
Repeat 1							
daGS>fkh RNAiRU486-	83	97	127	2			
daGS>fkh RNAiRU486+	37	64	113	1			
chico;daGS>fkh RNAiRU486-	90	113	115	2	8.43	16.49	3.89E-08
chico;daGS>fkh RNAiRU486+	38	66.5	122	2	2.70	3.91	0.61

Genotype/Treatment	Median Lifespan (days)	Maximum lifespan	n Dead	n Censored	% Median change (vs wt)	% Maximum change (vs wt)	p-value (log rank)
Repeat 2							
daGS>fkh RNAiRU486-	73	82.5	144	3			
daGS>fkh RNAiRU486+	29	49	142	1			
chico;daGS>fkh RNAiRU486-	75.5	89.5	142	3	3.42	8.48	2.06E-04
chico;daGS>fkh RNAiRU486+	26.5	47.5	150	0	-8.62	-3.06	0.15

E.Survival data for daGS> UAS-fkh RNAi +/- rapamycin (related to Figure 3E)

Genotype/Treatment	Median Lifespan (days)	Maximum lifespan	n Dead	n Censored	% Median change (vs -Rapa)	% Maximum change (vs -Rapa)	p-value (log rank)
Repeat 1							
daGS>fkh RNAiRU486-	69	73.5	147	5			
daGS>fkh RNAiRU486+	24.5	31.5	150	3			
daGS>fkh RNAiRU486- Rapa+	73.5	80.5	137	12	6.52	9.52	2.37E-08
daGS>fkh RNAiRU486+ Rapa+	24.5	31.5	146	4		0.00	0.82

Genotype/Treatment	Median Lifespan (days)	Maximum lifespan	n Dead	n Censored	% Median change (vs -Rapa)	% Maximum change (vs -Rapa)	p-value (log rank)
Repeat 2							
daGS>fkh RNAiRU486-	69.0	78.0	128	5			
daGS>fkh RNAiRU486+	27.0	45.5	114	10			
daGS>fkh RNAiRU486- Rapa+	73.5	89.0	106	8	6.52	14.10	3.05E-04
daGS>fkh RNAiRU486+ Rapa+	27.0	41.0	125	3		-9.89	0.34

Table S3- Details of CPH analysis. Related to Figures 3, 4AC and 5C.

A. Lifespan daGS;UAS-InRDN> +/- fkh RNAi(related to Figure 3AB)

Cox Proportional Hazard (CPH) analysis

Coefficient	estimate	exp(coeff)	SE (coeff)	z	p
RU486	0.07415	1.07697	0.12121	0.612	0.5407
InRDN	-0.32604	0.72178	0.12152	-2.683	0.0073 **
fkh RNAi	-1.35544	0.25783	0.12946	-10.47	< 2e-16 ***
RU486:InRDN	-0.67008	0.51167	0.17101	-3.918	8.91e-05 ***
RU486:fkh RNAi	3.31408	27.49708	0.18504	17.91	< 2e-16 ***
InRDN:fkh RNAi	0.12494	1.13308	0.17424	0.717	0.4733
RU486:InRDN vs fkh RNAi	0.48914	1.63091	0.24198	2.021	0.0432 *

B. Lifespan daGS;chico> +/- fkh RNAi(related to Figure 3CD)

Cox Proportional Hazard (CPH) analysis

Coefficient	estimate	exp(coeff)	SE (coeff)	z	p
RU486	-0.05856	0.94312	0.1355	-0.432	0.66563
chico	-0.66237	0.51563	0.13714	-4.83	1.37e-06 ***
fkh RNAi	-0.15165	-0.15165	0.12239	-1.239	0.21533
RU486 : chico	0.08379	1.0874	0.19071	0.439	0.66039
RU486 : fkh RNAi	3.4651	31.97972	0.21699	15.969	< 2e-16 ***
chico : fkhRNAi	-0.18896	0.82782	0.18949	-0.997	0.31868
RU486:chico vs fkh RNAi	0.76163	2.14177	0.26825	2.839	0.00452 **

C. Lifespan daGS> fkh RNAi +/- rapamycin (related to Figure 3E)

Cox Proportional Hazard (CPH) analysis

Coefficient	estimate	exp(coeff)	SE (coeff)	z	p
RU486	4.1513	63.5159	0.2791	14.876	< 2e-16 ***
Rapamycin	-0.6045	0.5464	0.1225	-4.936	7.99e-07 ***
RU486 : Rapamycin	0.5865	1.7976	0.1698	3.454	0.000552 ***

D. Paraquat resistance daGS;UAS-InRNDN> +/- fkh RNAi(related to Figure 4A)

Cox Proportional Hazard (CPH) analysis

Coefficient	estimate	exp(coeff)	SE (coeff)	z	p
RU486	-0.4351	0.6472	0.1486	-2.928	0.00342 **
fkh RNAi	-0.2492	0.7794	0.1503	6.996	0.07896
RU486: fkhRNAi	-0.1071	0.8985	0.2052	-0.522	0.60192

E. Starvation resistance daGS;UAS-InRNDN> +/- fkh RNAi(related to Figure 4C)

Cox Proportional Hazard (CPH) analysis

Coefficient	estimate	exp(coeff)	SE (coeff)	z	p
RU486	-0.59455	0.55181	0.14653	-4.057	4.96e-05 ***
fkh RNAi	0.07632	1.07931	0.13956	0.547	0.584
RU486: fkhRNAi	0.83095	2.29551	0.20347	4.084	4.43e-05 ***

F. Lifespan TiGS> fkh RNAi +/- rapamycin (related to Figure 5C)

Cox Proportional Hazard (CPH) analysis

Coefficient	estimate	exp(coeff)	SE (coeff)	z	p
RU486	0.02691	1.02728	0.02054	1.31	0.19
Rapamycin	-0.24298	0.78429	0.01997	-12.167	< 2e-16 ***
RU486 : Rapamycin	0.22289	1.24968	0.02861	7.792	6.66e-15 ***

Table S4- FKH is required for IIS-induced starvation resistance. Related to Figure 4A-C.

A. Paraquat resistance data for da*GS;UAS-InRDN>GFP RNAi or FKH RNAi* +/-RU486 (related to Figure 4A)

Genotype/Treatment	Med Survival (days)	Max Survival (days)	n Dead	p-value (log rank) vs -RU
Repeat 1				
daGS;InRDN>GFP RNAiRU486-	1.24	2.23	99	
daGS;InRDN>GFP RNAiRU486+	1.24	-	85	5.89E-04
daGS;InRDN>fkh RNAiRU486-	1.24	2.23	103	
daGS;InRDN>fkh RNAiRU486+	2.23	2.23	98	1.47E-07

Genotype/Treatment	Med Survival (days)	Max Survival (days)	n Dead	p-value (log rank) vs -RU
Repeat 2				
daGS;InRDN>GFP RNAiRU486-	0.60	2.13	94	
daGS;InRDN>GFP RNAiRU486+	1.15	7.92	100	4.43244E-05
daGS;InRDN>fkh RNAiRU486-	0.60	1.56	98	
daGS;InRDN>fkh RNAiRU486+	1.04	3.56	97	4.98E-10

B. DDT resistance data for da*GS;UAS-InRDN>GFP RNAi or FKH RNAi* +/-RU486 (related to Figure 4B)

Genotype/Treatment	Med Survival (days)	Max Survival (days)	n Dead	p-value (log rank) vs -RU
Repeat 1				
daGS;InRDN>GFP RNAiRU486-	2.6	3.7	90	
daGS;InRDN>GFP RNAiRU486+	3.7	-	88	1.34E-03
daGS;InRDN>fkh RNAiRU486-	2.6	3.7	96	
daGS;InRDN>fkh RNAiRU486+	2.6	3.7	92	2.80E-04

Genotype/Treatment	Med Survival (days)	Max Survival	n Dead	p-value (log rank) vs -RU
Repeat 2				
daGS;InRDN>GFP RNAiRU486-	3.61	4.65	91	
daGS;InRDN>GFP RNAiRU486+	4.13	6.61	92	3.34E-08
daGS;InRDN>fkh RNAiRU486-	3.19	4.65	100	
daGS;InRDN>fkh RNAiRU486+	4.13	-	104	2.32E-12

C.Starvation resistance data for daGS;UAS-InRDN>GFP RNAi or FKH RNAi +/-RU486 (related to Figure 4C]

Genotype/Treatment	Med Survival (days)	Max Survival (days)	n Dead	p-value (log rank) vs -RU
Repeat 1				
daGS;InRDN>GFP RNAiRU486-	7.64	8.61	102	
daGS;InRDN>GFP RNAiRU486+	8.61	9.51	95	3.67E-05
daGS;InRDN>fkh RNAiRU486-	7.64	8.61	105	
daGS;InRDN>fkh RNAiRU486+	7.64	8.61	101	0.085

Genotype/Treatment	Med Survival (days)	Max Survival (days)	n Dead	p-value (log rank) vs -RU
Repeat 2				
daGS;InRDN>GFP RNAiRU486-	6.99	8.63	90	
daGS;InRDN>GFP RNAiRU486+	7.60	9.54	88	5.58E-04
daGS;InRDN>fkh RNAiRU486-	6.15	7.60	96	
daGS;InRDN>fkh RNAiRU486+	6.21	6.55	92	0.70

Table S5- Intestinal overexpression of FKH extends lifespan. Related to Figures 5 and 7E.

A. Survival data for Ti*GS>UAS-fkh* (related to Figure 5A)

Genotype/Treatment	Med Lifespan (days)	Max Lifespan (days)	n Dead	n Censored	% Median change	% Maximum change	p-value (log rank) vs control
Repeat 1							
RU486-	66	78	219	7			
RU486+	69	82	212	5	4.5	5.13	0.001049071
Repeat 2							
RU486-	74	88	148	3			
RU486+	79	97	147	3	6.7	10.23	0.000153624
Repeat 3							
RU486-	103	114	119	1			
RU486+	108	124	119	2	4.8	8.77	4.16098E-05

B. Survival data for Ti*GS>fkh RNAi +/- rapa* (related to Figure 5C)

Genotype/Treatment	Med Lifespan (days)	Max Lifespan (days)	n Dead	n Censored	% Median change	% Maximum change	p-value (log rank) vs -rapa control
TiGS>fkh RNAiRU486-	80	94	146	1			
TiGS>fkh RNAiRU486+	80	94	146	2			
TiGS>fkh RNAiRU486- Rapa+	89	98	148		11.25	4.255319149	4.60E-10
TiGS>fkh RNAiRU486+ Rapa+	82	91.5	146	1			0.575678595

C. Survival data for Ti*GS>UAS-InRDN* (related to Figure 5D)

Genotype/Treatment	Med Lifespan (days)	Max Lifespan (days)	n Dead	n Censored	% Median change	% Maximum change	p-value (log rank) vs control
Repeat 1							
RU486-	85	104	147	3			0.014845219
RU486+	87.5	116.5	141	9	3	12	
Repeat 2							
RU486-	76	92	131	5			
RU486+	80.5	101.5	142	4	6	10.3	0.000289316

D. Survival data for Ti*GS;UAS-fkh RNAi> UAS-InRDN* (related to Figure 5E)

Genotype/Treatment	Med Lifespan (days)	Max Lifespan (days)	n Dead	n Censored	% Median change	% Maximum change	p-value (log rank) vs control
Repeat 1							
RU486-	83	94.5	126	15			
RU486+	73	87	136	4	-12	-7	8.97448E-07
Repeat 2							
RU486-	78	87.5	134	7			
RU486+	73.5	87.5	145	3	-5.7	0	0.000300082

E. Survival data for Ti*GS>UAS-fkh RNAi* (related to Figure 5E)

Genotype/Treatment	Med Lifespan (days)	Max Lifespan (days)	n Dead	n Censored	% Median change	% Maximum change	p-value (log rank) vs control
Repeat 1							
RU486-	87.5	105	144	1			
RU486+	83	106	141	1			0.08
Repeat 2							
RU486-	78	92	89	3			0.829119039
RU486+	76	94	79	10			

F. Survival data for *GS5966>UAS-fkh* (related to Figure 7E)

Genotype/Treatment	Med Lifespan (days)	Max Lifespan (days)	n Dead	n Censored	% Median change	% Maximum change	p-value (log rank) vs control
RU486-	71	87	139	12			
RU486+	82	92	139	8	15.4929577	5.747126437	2.03614E-07

Table S6- FKH is required for reduced IIS-induced longevity. Related to Figures S2B-D.

A. Survival data for da*GS;UAS-InRDN>GFP RNAi* +/-RU486 (related to Figure S2B)

Genotype/Treatment	Med Lifespan (days)	Max Lifespan (days)	n Dead	n Censored	% Median change (vs -RU)	% Maximum change (vs -RU)	p-value (log rank) vs control
daGS>GFP RNAiRU486-	90.5	87.5	144	4			
daGS>GFP RNAiRU486+	90.5	87.5	143	2	0.00	0.00	0.79
daGS;InRDN>GFP RNAiRU486-	76.0	80.5	131	2			
daGS;InRDN>GFP RNAiRU486+	80.5	92	146	4	5.92	14.29	7.91E-11

B. Survival data for da*GS;UAS-InRDN>fkh RNAi 2* +/-RU486 (related to Figure S2C)

Genotype/Treatment	Med Lifespan (days)	Max lifespan	n Dead	n Censored	% Median change (vs -RU)	% Maximum change (vs -RU)	p-value (log rank)
daGS>fkh RNAi2 RU486-	80.5	92	134	7			
daGS>fkh RNAi 2 RU486+	76	87.5	149	1	-5.590062112	-4.8913043	0.007503122
daGS;InRDN>fkh RNAi 2 RU486-	80.5	92	143	3			
daGS;InRDN>fkh RNAi 2 RU486+	76	90	123	7	-5.590062112	-2.173913	0.019521489
					daGS>fkh RNAiRU486	vs.	**0.623024154**
					daGS;InRDN>fkh RNAiRU486+		

D.Survival data for daGS;chico> UAS-fkh RNAi(related to Figure S2D)

Genotype/Treatment	Med Lifespan (days)	Max Lifespan (days)	n Dead	n Censored	% Median change (vs wt)	% Maximum change (vs wt)	p-value (log rank)
daGS>fkh RNAi2 RU486-	83	101.5	112				
daGS>fkh RNAi2 RU486+	85	106	118	1			
chico;daGS>fkh RNAi2 RU486-	87.5	120	77	3	5.42	18.23	3.15E-04
chico;daGS>fkh RNAi2 RU486+	85	103	78	3		-2.83	0.80

Table S7- GFP or FKH RNAi alone does not affect survival to stress. Related to Figure S4.

A. Paraquat resistance data for da*GS>GFP RNAi or FKH RNAi* +/-RU486 (related to Figure S4A)

Genotype/Treatment	Med Survival (days)	Max Survival (days)	n Dead	-value (log rank) vs -RU
daGS>GFP RNAiRU486-	1.24	2.23	97	
daGS>GFP RNAiRU486+	1.24	2.23	98	7.29E-02
daGS>fkh RNAiRU486-	1.24	2.23	102	
daGS>fkh RNAiRU486+	1.24	2.23	95	7.32E-01

B. DDT resistance data for da*GS>GFP RNAi or FKH RNAi* +/-RU486 (related to Figure S4B)

Genotype/Treatment	Med Survival (days)	Max Survival (days)	n Dead	-value (log rank) vs -RU
daGS>GFP RNAiRU486-	2.55	2.55	99	
daGS>GFP RNAiRU486+	2.55	2.55	100	9.30E-01
daGS>fkh RNAiRU486-	2.55	3.70	101	
daGS>fkh RNAiRU486+	2.55	3.70	99	4.41E-01

C. Starvation resistance data for da*GS>GFP RNAi or FKH RNAi* +/-RU486 (related to Figure S4C)

Genotype/Treatment	Med Survival (days)	Max Survival (days)	n Dead	-value (log rank) vs -RU
daGS>GFP RNAiRU486-	8.23	10.56	99	
daGS>GFP RNAiRU486+	7.60	9.54	94	9.47E-01
daGS>fkh RNAiRU486-	8.23	9.54	96	
daGS>fkh RNAiRU486+	7.60	8.63	94	1.23E-01

Table S8- Survival data Related to Figure S5

A. Survival data for S106>*UAS-fkh* (related to Figure S5A)

Genotype/Treatment	Med Lifespan (days)	Max lifespan (days)	n Dead	n Censored	% Median change	% Maximum change	p-value (log rank) vs control
RU486-	68.5	85	211	6			
RU486+	68.5	85	209	6			0.093464441

B. Survival data for GS5961>*UAS-fkh* (related to Figure S5B)

Genotype/Treatment	Median Lifespan (days)	Maximum lifespan (days)	n Dead	n Censored	% Median change	% Maximum change	p-value (log rank) vs control
RU486-	80	99	134	9			
RU486+	82.5	101	132	6			0.215031421

C. Survival data for UAS-fkh>*+* (related to Figure S5D)

Genotype/Treatment	Median Lifespan (days)	Maximum lifespan (days)	n Dead	n Censored	% Median change	% Maximum change	p-value (log rank) vs control
RU486-	78.5	90	85	7			
RU486+	78.5	92.5	86				0.484519478

D. Survival data for daGS>*+* (related to Figure S5E)

Genotype/Treatment	Median Lifespan (days)	Maximum lifespan (days)	n Dead	n Censored	% Median change	% Maximum change	p-value (log rank) vs control
RU486-	80	93	94	2			
RU486+	80	93	95	2			0.675801839

E. Survival data for TiGS>*+* (related to Figure S5F)

Genotype/Treatment	Median Lifespan (days)	Maximum lifespan (days)	n Dead	n Censored	% Median change	% Maximum change	p-value (log rank) vs control
RU486-	74.5	88	147	3			
RU486+	76	88	144	4			0.83062806

F. Survival data for GS5961>+ (related to Figure S5G)

Genotype/Treatment	Median Lifespan (days)	Maximum lifespan (days)	n Dead	n Censored	% Median change	% Maximum change	p-value (log rank) vs control
RU486-	89	106	150	1			
RU486+	92	106	143	2			0.38072779

G. Survival data for S106>+ (related to Figure S5H)

Genotype/Treatment	Median Lifespan (days)	Maximum lifespan (days)	n Dead	n Censored	% Median change	% Maximum change	p-value (log rank) vs control
RU486-	75	86	135	4			
RU486+	74	87.5	134	4			0.489215204

Supplemental Experimental Procedures

Lifespan Experiments

RU486 (Sigma) dissolved in ethanol was added to a final concentration of 200 μM, unless otherwise indicated. Rapamycin (LC Laboratories) was diluted in ethanol and added to SYA food at 100 μM concentration. Stocks were backcrossed for at least six generations into the wild-type outbred *wDahomey* population. For lifespans, flies were sorted into experimental vials at a density of 10 or 15 flies per vial. Flies were transferred to fresh vials three times a week, and deaths/censors were scored during the transfer.

Fly Stocks

The following stocks were used: *UAS-InR*^{DN} (*K1409A*) and *UAS-GFP-RNAi* stocks were obtained from the Bloomington Stock Centre. *UAS-fkh-RNAi* line was obtained from Vienna stock center (37062). *daGS* was kindly provided by Veronique Monnier (Tricoire et al., 2009). *TiGS* and $S_1$106 drivers were described in Poirier et al. 2008 (Poirier et al., 2008). The alternative *UAS-fkh-RNAi* (*fkh RNAi 2*) stock was kindly provided by Michael Junger (Bulow et al., 2010). d*foxo*$^{\Delta/\Delta}$ line was described in Slack et al., 2011 (Slack et al., 2011) . To create *UAS-FKH lines*, *Fork Head* genomic sequences encoding the full-length wild-type fkh protein sequence open reading frame were cloned into pENTR-D vector, confirmed by sequencing and transferred to the pUASg.attB (Bischof et al., 2007)P-element-based vector using standard Gateway cloning techniques (Invitrogen).

Paraquat, DDT and Fecundity Assays

Paraquat, DDT and fecundity assays were carried out as described by Slack et al., (Slack et al., 2011) with the following modification: the final concentration of DDT used in the xenobiotic assay was increased to 0.06% (w/v) DDT.

Dietary Restriction

The DR protocol was described in detail in Bass et al., (Bass et al., 2007)

S2 cell culture and transfections

S2 cells were routinely cultured in Schneider's insect medium (Sigma) supplemented with 10% FBS. S2 cell transfections were carried out by using Effectene transfection reagent (Qiagen) according to manufacturer's instructions. To express FKH-HA and dAKT-FLAG in S2 cells, full length wild type FKH and dAKT open reading frames were cloned into pAHW and pAFW *Drosophila* Gateway vectors (a gift from Martin Junger) respectively by using standard Gateway cloning techniques (Invitrogen).

Preparation of PhosTag SDS-PAGE Gels

24 hour following transfections, S2 cells were serum starved for 2 hours and stimulated with 1 μM human insulin (Sigma) for the indicated duration. Protein extracts were prepared by lysing the cells in RIPA buffer (NEB labs) containing PhoSTOP (Roche) and Complete Protease inhibitors (Roche). SDS-PAGE gels were prepared at 7.5% acrylamide concentration and contained 50 mM Phos-Tag^{TM} (Wako). Gels were run at 50 V until samples entered the top one third of the resolving gel at which point the voltage was increased to 140 V.

Immunoprecipitation

Prior to immunoprecipitations (IPs), S2 cells were stimulated with 1 μM human insulin (Sigma) for 15 minutes. S2 cells were washed in cold PBS and lysed in cold lysis buffer (50 mM HEPES-KOH pH 8, 100 mM KCL, 5 mM EDTA, 10% glycerol, 0.5% NP-40) containing PhoSTOP (Roche) and Complete

Protease Inhibitors (Roche). IPs were carried out by using either anti-HA coupled agarose beads (Sigma,) or anti-FLAG coupled agarose beads (Sigma, F2426) according to manufacturer's instructions.

Immunoblotting

Protein samples were resolved at 140 V by SDS-PAGE on precast 4–12% Bis-Tris polyacrylamide gels (Novex) and transferred onto nitrocellulose membranes in a Trans-Blot apparatus (Biorad). Membranes were blocked in TBSTw (Tris-buffered saline (150 mM NaCl, 20 mM Tris pH 7.5) + 0.05% Tween 20) and 5% (w/v) semi-skimmed milk for 1 h at room temperature and then incubated for 1 h with primary antibody in TBSTw. After washing three times for 5 min with TBSTw, the membranes were incubated in an appropriate horseradish peroxidase-linked secondary antibody (1 : 10 000) for 1 h in TBSTw at room temperature. Finally, the membranes were washed as above in Tris-buffered saline plus 0.2% Triton X and immune-complexes detected by enhanced chemiluminescence (ECL; GE Healthcare). Primary antibodies and dilutions used were as follows: anti- HA (Sigma, H3663) 1:2000; rabit anti-FKH (Abrams et al., 2006) (gift from Prof Deborah Andrew) 1:1000; anti-FLAG (Sigma, F3165) 1:2000. Secondary antibodies and dilutions used in were as follows: goat anti-rabbit (abcam, ab6721) 1:5000; goat anti-mouse (Abcam, ab6789) 1:5000.

Immunohistochemistry on adult guts

The following antibodies were used in in immunohistochemistry of guts; primary antibodies: rabbit anti-FKH (Abrams et al., 2006) (gift from Prof Deborah Andrew) 1:1000, rabbit anti-PH3 (Cell Signalling 9701) 1:500. Nile Red was diluted in PBS at 1:2000 from the stock solution prepared at 0.5mg/ml in acetone. Secondary antibodies: Alexa Fluor 488 donkey anti-rabbit (A21206) 1:1000. Guts were dissected in ice cold PBS. Insulin stimulation involved incubation of adult guts at room temperature in Schneider's medium (Sigma) containing 1 μM human insulin (Sigma). Guts were immediately fixed in 4% formaldehyde for 15 minutes, serially dehydrated in MeOH, stored at -20°C, and subsequently stained. Guts were washed in 0.2% 331 Triton-X / PBS, blocked in 5% bovine serum albumin / PBS, incubated in primary antibody overnight at 4°c and in secondary for 2 h at RT. 10-15 guts per condition were mounted, scored and imaged as described above.

Immunohistochemistry on S2 cells

The following antibodies were used in in immunohistochemistry of S2 cells; primary antibodies: mouse anti-HA (Sigma, H3663) 1:1000. Secondary antibodies: Alexa Fluor594 phalloidin (A12381) 1:1000; Alexa Fluor 488 donkey anti-rabbit (A21206) 1:1000. S2 cells were cultured on Concanavalin A coated coverslips in six-well plates overnight. Cells were rinsed in PBS for 3 min followed by a 3 min fixation in PBS containing 4 % paraformaldehyde (diluted from 16% ampoules). The fixative was removed by a 2 min wash in PBS. Cells were permeabilised by a 5 min incubation in PBS + 0.5% Triton X-100 followed by a 1 h block in PBS containing 3 per cent BSA. Subsequently, cells were washed in PBS + 0.1% Triton X-100 for 5 min. Primary antibody was diluted in PBS + 0.3% BSA + 0.1% Triton X-100 and incubated on the cells for 1 h at room temperature. Cells were washed twice for 5 min followed by a 1 h secondary antibody incubation diluted in PBS + 0.3% BSA + 0.1% Triton X-100. Subsequently, 45 min washes were performed in PBS + 0.1% Triton X-100. DAPI was included in the penultimate wash at 0.1 μg ml^{-1} concentration.

Imaging

Images were captured using the Zeiss LSM 700 confocal microscope and processed using ImageJ software.

Smurf Assay

Gut barrier efficiency was analyzed by placing flies on blue food (minimum of 200 flies per condition at 10 week of age) prepared using 2.5% (w/v) FD&C blue dye no. 1 (Fastcolors) as previously described (Rera et al., 2012), except flies were kept 24 hours on the blue food before the Smurf phenotype was scored.

Generation of *Irs1* conditional KO and tissue-specific KO mice

Generation of *Irs1loxP/loxP* mice was described in Essers et al., 2016(Essers et al., 2016) For tissue-specific KO of *Irs1*, *Irs1loxP/loxP* mice were crossed with mice expressing Cre-recombinase under the control of the villin promoter (*Vill*Cre mice, The Jackson Laboratory, stock number 004586). Breeding *Irs1loxP/loxP Vill*Cre mice with *Irs1loxP/loxP*, produced mice with intestinal *Irs1* deletion (denoted as *VillCre::Irs1*$^{lox/lox}$) and littermate controls.

RNA isolation and quantitative PCR

RNA was isolated from 14 fly guts per sample or mouse small intestine with TRIzol (Invitrogen) and cDNA was synthesized by using SuperScript II Reverse Transcriptase (Invitrogen) according to manufacturers instructions. qPCR was carried out by using Power SYBR Green PCR Master Mix (ABI) on ABI Prism 7000.
The following primers sequences were used in the analysis.
Fork head (forward-ATTCGACATTCGCTGAGGTT, reverse-TCGAACATATTCCCCGAA)

Drosophila Actin5C (forward-CACACCAAATCTTACAAAATGTGTGA, reverse-AATCCGGCCTTGCACATG)
Zip42C.1 (forward- CTGTGGAACCTTGCTGTACG, reverse-AGGTTAGGCTGTCATCACCC)
Mdr50 (forward-GGCGCCAAACTAGAGGATTC, reverse-CGTACCGAAAGAGCTGGAAG)
Ctr1B (forward-ATCACGGCTCGGATGACAG, reverse-ACAGGAAGGACACCAGGAAG)
Pmp70 (forward-CCTCAGAGGCCCTACATGAC, reverse-CACGTCGATCCAGTCCTCAA)
CG1208 (forward- CCACAACTGGCGGTATTTCC, reverse- GGCGAAACTAAGCGTGATCC)
MtnB (forward-AAGGGTTGTGGAACAACTGC, reverse- GTCCTTGGGCCCATTCTT)
MtnC (forward-AAAGGCTGCGGAACAAACT, reverse-ATCTTTGGGGCCATTCTTG)
Abcd3 (forward-GGGAGAAGCAGACAATCCAC, reverse-CCGAAAGAAAATGAAATTATGTAGG)
Slc39a3 (forward-TGGCGTATTCCTGGCTACAT, reverse-GAAACCCACCATCATGAGCG)
Slc2a8 (forward- TCTTCATTGCTGGCTTTGCG, reverse-TTGGTGAGGACACAGATGCC)
Mouse Actin (forward- AACCGTGAAAAGATGACCCAG, reverse-CACAGCCTGGATGGCTACGTA

Expression and Purification of FKH

Full length, wild-type *Fork Head* coding sequence was cloned into pENTR-D-TOPO vector, transferred into pDEST17 expression vector and confirmed by sequencing. Rosetta™(DE3)pLysS Competent Cells (Novagen) were used to express the recombinant FKH. Bacterial cells were lysed via sonication in PBS+ 500 mM NaCl+ 25 mM immidizole containing Complete protease inhibitors (Roche) and lysates cleared by centrifugation. Purification of the protein was carried out on an Akta purifier system (GE Healthcare) and a Ni-NTA column. FKH was eluted with an imidazole gradient (25mM to 500 mM) in PBS + 500 mM NaCl. The fractions to be used for subsequent essays were determined by SDS-Page and Coomassie staining. Successful purification of recombinant FKH-his protein was confirmed by immunoblotting with an anti-His antibody (Sigma, H1029).

***In vitro* kinase reactions**

In vitro kinase reactions were carried out in a total volume of 30 µl containing 1X Kinase buffer (Cell signaling, 9802)+ 10 µM ATP + 5 µCi [γ-33P]ATP , 3µl of purified FKH and 0.1 µg of recombinant kinase. Active recombinant kinases mTOR kinase (SRP0364) and Akt1 (A8729) were purchased from Sigma and GSK fusion protein was purchased from Cell signaling (9237). Kinase reactions were incubated at 30°C for 20 minutes and reactions were stopped by adding sample buffer containing 10 mM DTT. Samples were subsequently boiled at 80°C for 10 minutes and run on 4–15% Mini-PROTEAN® TGX™ Precast Protein Gels (BioRad). The gel was dried on a Whatman 3.0 paper, and exposed to autoradiogram for 24 hours by using Amersham Hyperfilm (GE Healthcare).

Library preparation for sequencing

Fly guts were micro-dissected in ice-cold RNAlater (Qiagen) solution and RNA was extracted using the QIAGEN total RNA isolation kit and quantified on an Agilent 2100 bioanalyser. Sample concentration and purity of RNA was measured on a NanoDrop spectrophotometer, and RNA integrity was assessed on an Agilent 2100 Bioanalyzer. Samples were processed using Illumina's TruSeq Stranded mRNA LT sample preparation kit (p/n RS-122-2101) according to manufacturer's instructions. Deviations from the protocol were as follows:
250ng total RNA was used as starting material. Fragmentation was carried out for 10mins instead of 8mins, and 14 cycles of PCR were used.
Briefly, mRNA was isolated from total RNA using Oligo dT beads to pull down Poly-Adenylated transcripts. The purified mRNA was fragmented using chemical fragmentation (heat and divalent metal cation) and primed with random hexamers. Strand-specific first strand cDNA was generated using Reverse Transcriptase and Actinomycin D. This allows for RNA dependent synthesis while preventing spurious DNA-dependent synthesis. The second cDNA strand was synthesised using dUTP in place of dTTP, to mark the second strand.
The resultant cDNA is then "A-tailed" at the 3' end to prevent self-ligation and adapter dimerisation. Full length TruSeq adaptors, containing a T overhang are ligated to the A-Tailed cDNA. These adaptors contain sequences that allow the libraries to be uniquely identified by way of a 6bp Index sequence. Successfully ligated fragments were enriched with 14 cycles of PCR. The polymerase is

unable to read through uracil, so only the first strand is amplified, thus making the library strand-specific.

Sequencing

Libraries to be multiplexed in the same run were pooled in equimolar quantities, calculated from Qubit and Bioanalyser fragment analysis.

Samples were sequenced on the NextSeq 500 instrument (Illumina, San Diego, US) using a 43bp paired end run resulting in >15million reads per sample. Sequencing was carried out by UCL Genomics at the UCL GOS Institute of Child Health.

Alignment and differential expression of RNA-seq data

Initial quality control of the raw files (fastq) was performed using FastQC (v0.11.4, http://www.bioinformatics.babraham.ac.uk/projects/fastqc/). The raw RNA-seq data comprised paired-end reads with read length of 43bp. Reads were aligned to the *Drosophila* reference genome (Ensembl BDGP6 release 84 and *Wolbachia* NCBI NC_002978.6) using tophat2(Trapnell et al., 2009) (--library fr-firststrand --no-coverage-search --keep-fasta-order --segment-length 20 --segment-mismatches 1). Additionally, we estimated the tophat's insert distance (--mate-inner-dist) and standard deviation (--mate-std-dev) by aligning the first 1M reads. Aligned reads were filtered by removing non-primary alignments, unmapped reads and mates, reads not mapped in proper pairs and alignments with MAPQ <20, using samtools(Li et al., 2009) (v1.3.1; -F 0x100 -F 0x004 -F 0x008 -q 20 -f 0x002). Read counts per gene identifier were computed using htseq-count(Anders et al., 2015) (v0.6.0; --stranded=reverse -t exon --idattr=gene_id --mode=intersection-strict). Differentially expressed genes were determined using DESeq2(Love et al., 2014) (v1.10.1) with default parameters. Genes were deemed differentially expressed if p-value ≤ 0.05 following a Benjamini and Hochberg correction for multiple hypothesis testing (default parameter in DESeq2).

Functional annotation and gene-set enrichment analysis

The Wilcoxon rank sum test, as implemented in Catmap (Breslin et al., 2004), was used to perform functional analysis, ie significant enrichment of Gene Ontology (GO) categories. FlyBase's (http://flybase.org) gene identifiers were mapped to Gene Ontology identifiers (FlyBase version FB2016_04). Ranks of genes were based on the p-value derived from the DESeq2 analysis for differential expression. GO categories were deemed statistically significant if the p-value derived from the Wilcoxon rank sum test was ≤ 0.05 after Benjamini and Hochberg correction for multiple hypothesis testing. Gene lists were sorted by log-fold change and p-value. For all experiments three sets of lists were derived; a gene list comprising most differentially up-regulated (log-fold change > 0) genes at the top of the list and most differentially down-regulated genes (log-fold change < 0) at the bottom of the list (termed up-to-down) and vice versa (termed down-to-up). Additionally, a list comprising genes sorted by p-value only was also generated. These ranked lists of genes were used to generate significance of GO categories. If a GO category was found to be statistically significant in the up-to-down list, this GO was referred to as up-regulated, i.e. a large enough proportion of the genes that are part of this GO category were found to be up-regulated or at the top of the list. If a GO category was found to be statistically significant in the down-to-up list, this GO was referred to as down-regulated, i.e. a large enough proportion of the genes that are part of this GO category were found to be down-regulated or at the top of the list. GO categories were deemed to be statistically significant if the p-value was ≤ 0.05 after Benjamini and Hochberg correction for multiple hypothesis testing.

Statistical significance of differential expression gene sets

Statistical significance of overlaps of genes in two expression experiments was determined using Fisher's exact test. To account for multiple hypotheses testing, a p-value cut-off of ≤1.0x10-05 was used. Statistical significance of GO term enrichment in genes that overlapped between two experiments was determined using a hypergeometric test in R.

***Ecc15* infection**

Erwinia carotovora carotovora15 (Ecc15) was grown in LB overnight at 30 °C and overnight cultures were spun down and pelleted and resuspended in a very small volume of fresh LB. OD was measured at 600 nm and cultures were resuspended so that the final OD_{600} = 200. Flies were pricked with a needle dipped in the bacterial solution in the thorax just above the right wing. As a control same number of flies were pricked with a needle dipped in LB alone and survival was monitored over the following 7-8 days.

SUPPLEMENTAL REFERENCES

Abrams, E.W., Mihoulides, W.K., and Andrew, D.J. (2006). Fork head and Sage maintain a uniform and patent salivary gland lumen through regulation of two downstream target genes, PH4alphaSG1 and PH4alphaSG2. Development *133*, 3517-3527.

Anders, S., Pyl, P.T., and Huber, W. (2015). HTSeq--a Python framework to work with high-throughput sequencing data. Bioinformatics *31*, 166-169.

Bass, T.M., Grandison, R.C., Wong, R., Martinez, P., Partridge, L., and Piper, M.D. (2007). Optimization of dietary restriction protocols in Drosophila. J Gerontol A Biol Sci Med Sci *62*, 1071-1081.

Bischof, J., Maeda, R.K., Hediger, M., Karch, F., and Basler, K. (2007). An optimized transgenesis system for Drosophila using germ-line-specific phiC31 integrases. Proceedings of the National Academy of Sciences of the United States of America *104*, 3312-3317.

Breslin, T., Eden, P., and Krogh, M. (2004). Comparing functional annotation analyses with Catmap. BMC Bioinformatics *5*, 193.

Bulow, M.H., Aebersold, R., Pankratz, M.J., and Junger, M.A. (2010). The Drosophila FoxA ortholog Fork head regulates growth and gene expression downstream of Target of rapamycin. PloS one *5*, e15171.

Essers, P., Tain, L.S., Nespital, T., Goncalves, J., Froehlich, J., and Partridge, L. (2016). Reduced insulin/insulin-like growth factor signaling decreases translation in Drosophila and mice. Sci Rep *6*, 30290.

Li, H., Handsaker, B., Wysoker, A., Fennell, T., Ruan, J., Homer, N., Marth, G., Abecasis, G., Durbin, R., and Genome Project Data Processing, S. (2009). The Sequence Alignment/Map format and SAMtools. Bioinformatics *25*, 2078-2079.

Love, M.I., Huber, W., and Anders, S. (2014). Moderated estimation of fold change and dispersion for RNA-seq data with DESeq2. Genome Biol *15*, 550.

Poirier, L., Shane, A., Zheng, J., and Seroude, L. (2008). Characterization of the Drosophila gene-switch system in aging studies: a cautionary tale. Aging cell *7*, 758-770.

Rera, M., Clark, R.I., and Walker, D.W. (2012). Intestinal barrier dysfunction links metabolic and inflammatory markers of aging to death in Drosophila. Proceedings of the National Academy of Sciences of the United States of America *109*, 21528-21533.

Slack, C., Giannakou, M.E., Foley, A., Goss, M., and Partridge, L. (2011). dFOXO-independent effects of reduced insulin-like signaling in Drosophila. Aging cell *10*, 735-748.

Trapnell, C., Pachter, L., and Salzberg, S.L. (2009). TopHat: discovering splice junctions with RNA-Seq. Bioinformatics *25*, 1105-1111.

Tricoire, H., Battisti, V., Trannoy, S., Lasbleiz, C., Pret, A.M., and Monnier, V. (2009). The steroid hormone receptor EcR finely modulates Drosophila lifespan during adulthood in a sex-specific manner. Mechanisms of ageing and development *130*, 547-552.
